# Supplementary material for: The gut microbiome and metabolome associate with Schistosoma mansoni infection and cardiovascular disease risk in Uganda
Source: Nat Commun. 2026 Feb 4;17:2351. doi: 10.1038/s41467-026-68983-3 (PMC12979582; doi:10.1038/s41467-026-68983-3)
Supplement: Supplementary file 1 — Supplementary Information [file 41467_2026_68983_MOESM1_ESM.pdf]

Supplementary figures: The gut microbiome and metabolome associate with *Schistosoma mansoni* infection and cardiovascular disease risk in Uganda.

Diet	Fish lovers	Meat lovers	Mixed diet	Fruits & Vegetables
Sm +ve	49	4	10	2
Sm -ve	26	0	23	5

Fisher's exact test, *p*=**0.6451**

Diet distribution(%) in the rural setting

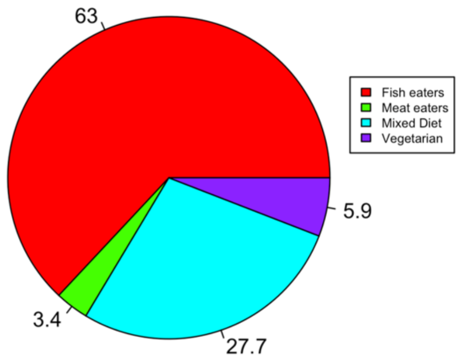

Diet type	Threshold (meal Days per week)
Fish lovers	>5 days (a)
Meat lovers	>3 days (b)
Fruits and Vegetables	> 3 days & 0 meat days (c)
Mixed Diet	<a & <b & < c

Supplementary Fig. 1: Diet distribution for the rural population

(A) *Distribution table showing the number of* S. mansoni-*infected and uninfected participants predominantly consuming each dietary pattern, including fish-based, meat-based, mixed, and fruit/vegetable-rich diets.*

(B) *Pie chart depicting the overall distribution of dietary patterns across all participants, presented as percentages.*

(C) *Schematic illustrating the algorithm used to classify participants into dietary categories, based on self-reported frequency (number of days per week) of consuming specific food types*

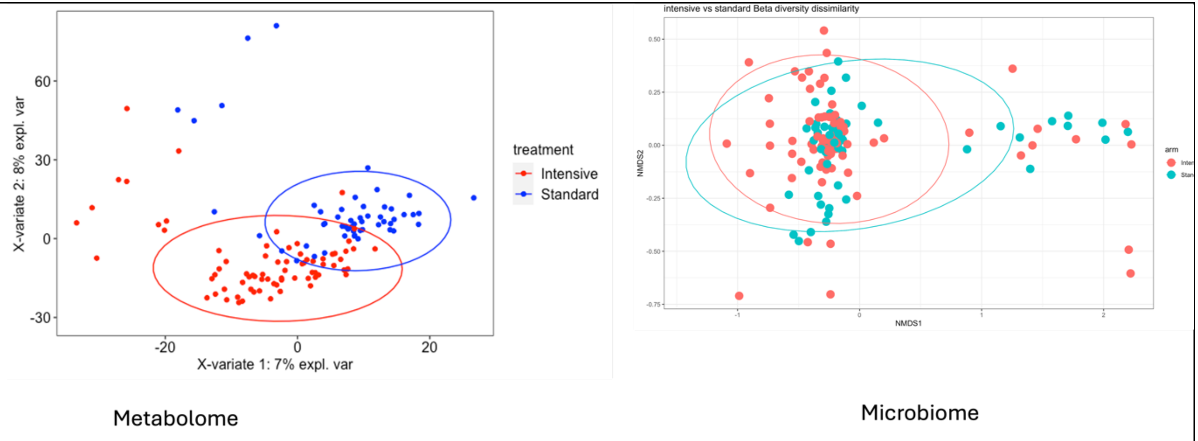

Supplementary Fig. 2: Comparison of metabolome and microbiome of participants living in rural community. *(**A)** PLSDA plot comparison of metabolomes between intensive and standard anthelminthic treatment. **(B)** PcoA plot showing beta diversity (using Bray-Curtis distance) comparison between intensive and standard anthelminthic treatment.*

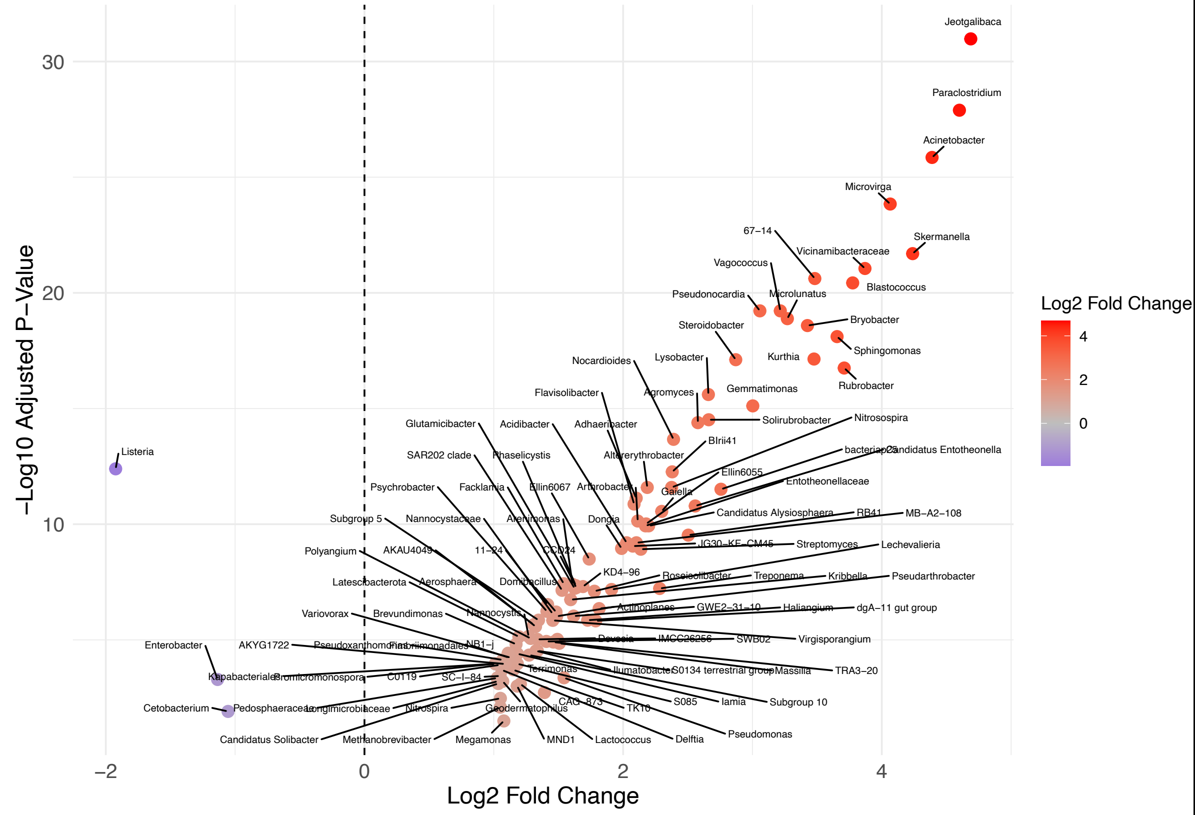

Supplementary Fig. 3: Differential abundance analysis. *Volcano plot showing the* log_2 *fold change in abundance (x-axis) versus the* $-log_{10}$ *adjusted* p-value *(y-axis) for microbial taxa differentially abundant between S. mansoni-infected and -*

uninfected individuals. Each point represents a microbial taxon. Taxa to the right of the vertical dashed line are enriched in S. mansoni-infected individuals, while those to the left are depleted. Points are colored by their log_2 fold change, with red indicating higher abundance and blue indicating lower abundance in S. mansoni-infected. Labels highlight All taxa with statistically significant differences (FDR-adjusted p < *0.05). Also, coloured taxa denoted by triangular dots are both significantly enriched in S. mansoni participants and associated with CVD risk. Those taxa shown by circular dots are significantly impacted by S. mansoni infection but are not associated with CVD risk.*

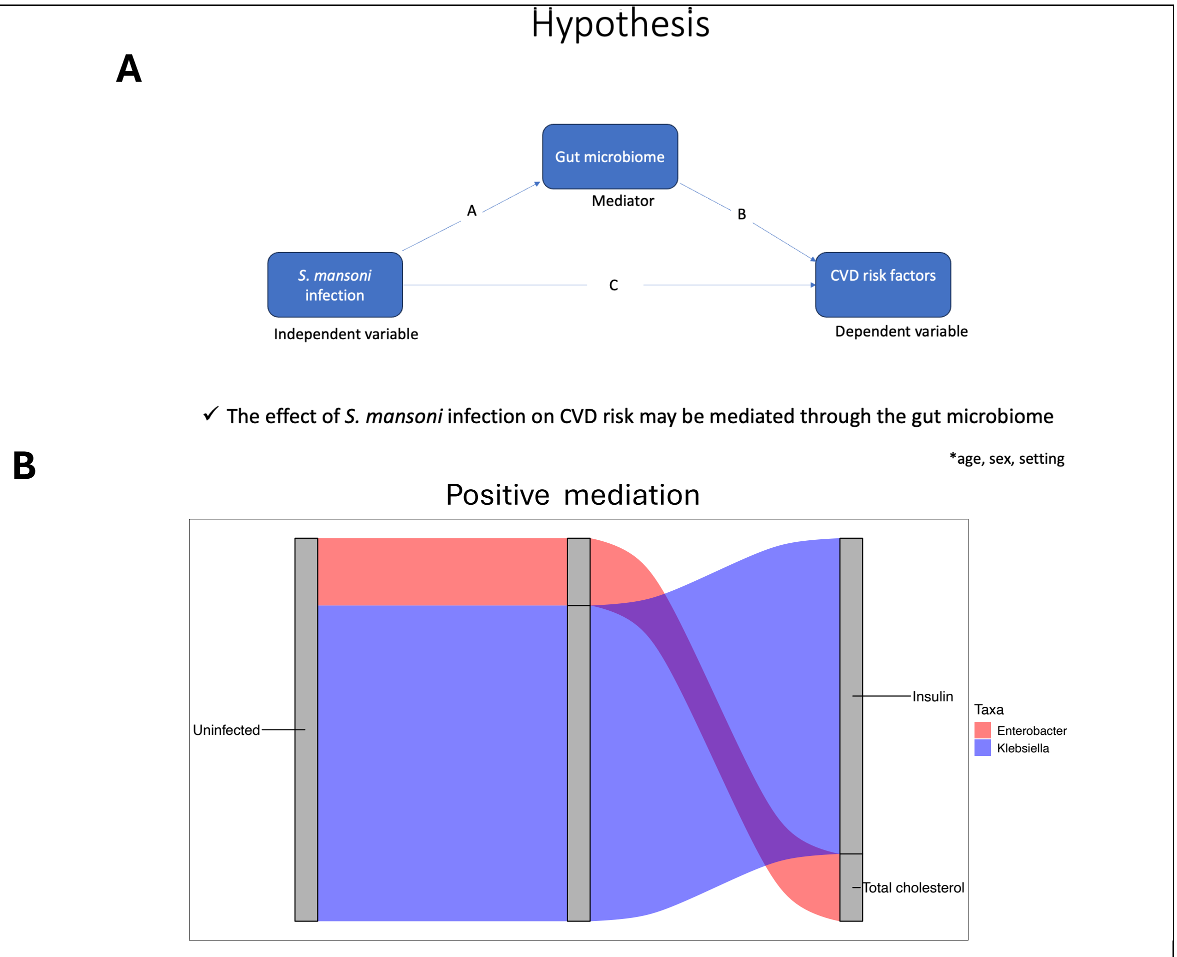

Supplementary Fig. 4: Positive mediation by microbes

(A) *Visualisation of the mediation analysis model showing hypothesis underlying the link between microbes, S. mansoni infection and CVD risk.* ***(B)*** *Mediation analysis illustrating the mediatory role of microbes in S.mansoni-driven CVD risk resolution. An alluvial plot showing microbes through which helminths may alter one's CVD risk.*

Results from mediation analysis using bias-correct non-parametric bootstrap method, and adjusting for age, sex and setting, using the mediation analysis function in pingouin python library. Microbial taxa shown were differentially abundant (p=0.05) in either the S.m+ and S.m- groups (shown the Y-axis). All the microbes above had a significant positive mediation effect (p<0.05, 97.5% CI) on CVD risk factors shown.

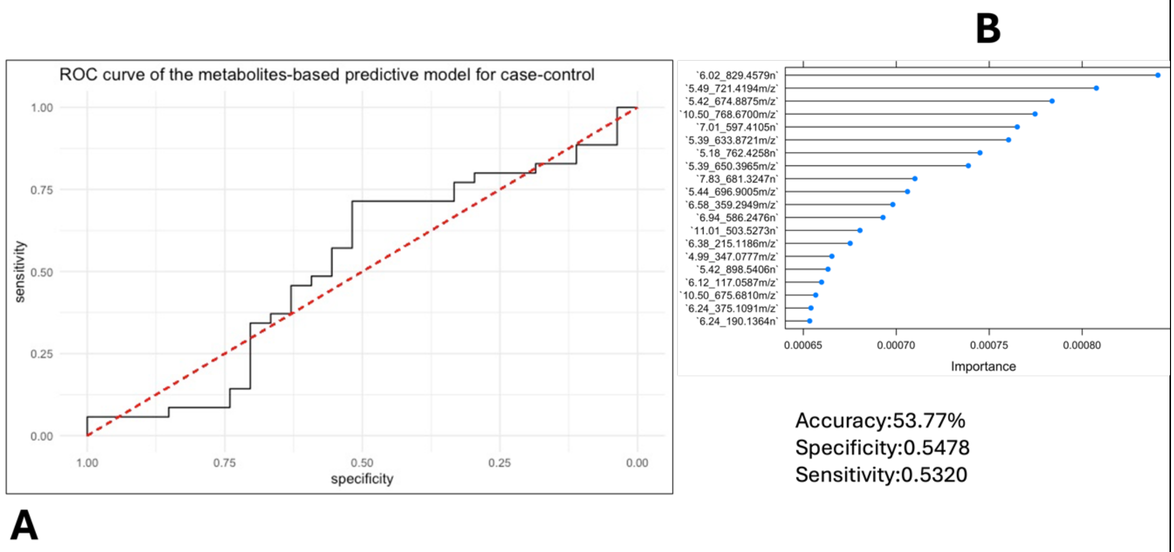

Supplementary Fig. 5: Prediction of infection status using the metabolite profile

(A) *Receiver operating characteristic (ROC) curve illustrating the performance of a metabolite-based classification model generated using partial least squares discriminant analysis (PLS-DA) to distinguish between groups. The model achieved an accuracy of 53.77%, with a specificity of 54.78% and a sensitivity of 53.20%.*

(B) *Variable importance in projection (VIP) plot highlighting the top contributing metabolites (based on m/z ratios) driving the model's classification. Metabolites with the highest VIP scores were considered the most influential in separating the groups and may reflect biologically relevant signatures.*

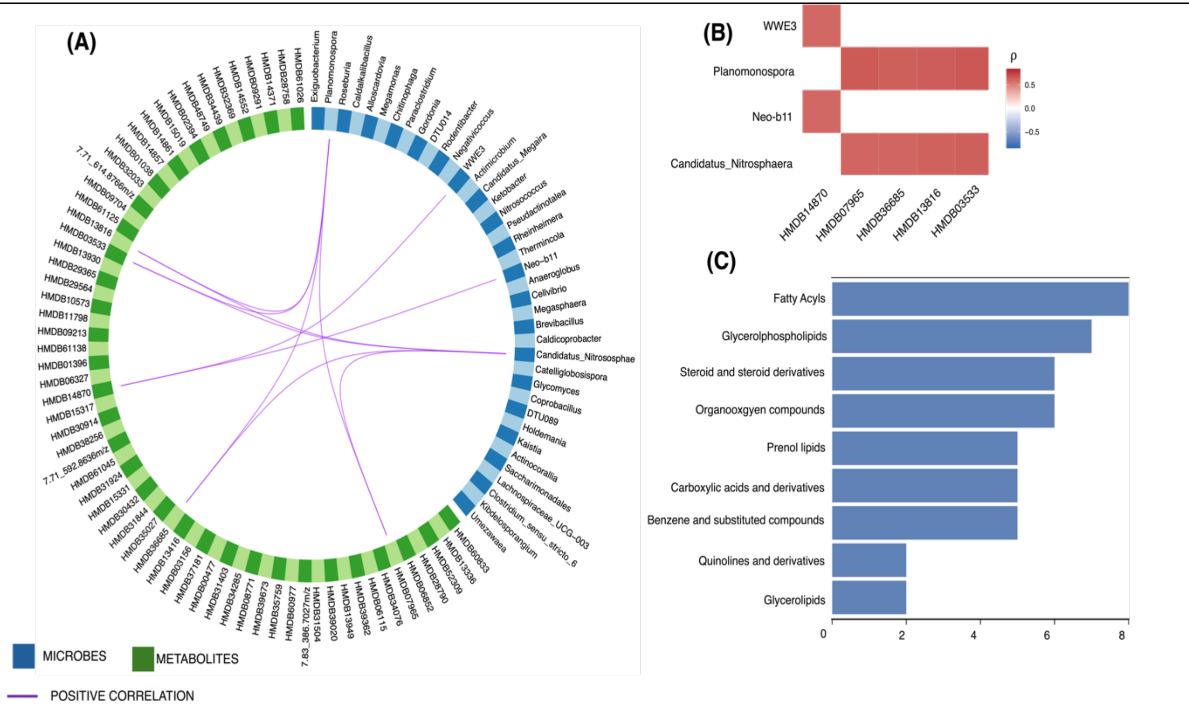

Supplementary Fig. 6: Insulin_associated microbes interaction with metabolites

***A** Circos plot plot showing the top most correlations between variables of microbiome (blue blocks) and metabolome (green blocks) data that were significantly associated with insulin resistance following association analysis described in Figure 3 where factors such as age, sex, BMI were adjusted for , and setting included as interaction term in our model. The correlation cut-off for this circos plot is r ≥ 0.65. **B** After extracting only those microbes and metabolites shown to interact in A, a heatmap was used to clearly show the most significant correlations between the microbiota and metabolites that were significantly associated withinsulin resistance. Correlation strength (spearmans correlation coefficient, r, value) is shown by the depth of the red colour (the deepest red indicates the strongest correlation positive correlation, and blue shows negative correlation). **C** Annotation of metabolites into classes. Going beyond individual metabolites, this annotation was done using the CLASSYFIRE to show the classes they fall under. The blue horizontal bars represent the number of metabolites matching with x-axes. The vertical axis represents the annotated classes of metabolites, and the graph shows the number of metabolites annotated to a class.*

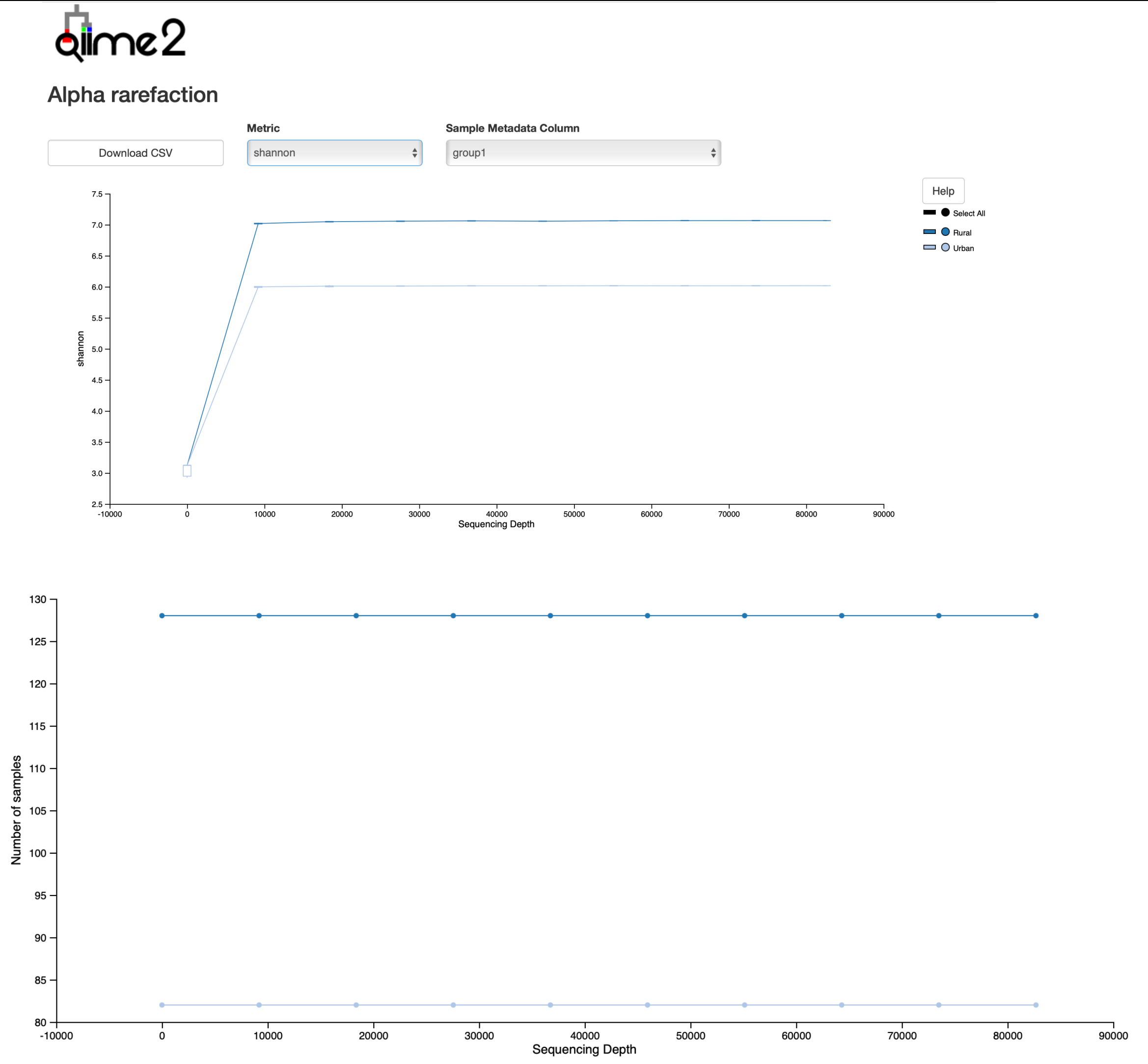

Supplementary Fig. 7. Rarefaction and species accumulation curves confirming adequate sequencing depth and sampling effort. (A) *Rarefaction curves showing the number of observed amplicon sequence variants (ASVs) as a function of sequencing depth for all samples. Curves approached a plateau, indicating that the rarefaction cutoff of 82,695 reads was sufficient to capture within-sample (alpha) diversity and that additional sequencing would yield minimal gain in new ASVs.* ***(B)*** *Species accumulation curve based on >10 randomly permuted samples, demonstrating a stable asymptote in species richness. This confirms that the overall sampling effort was sufficient to characterise between-sample (gamma) diversity and that the dataset adequately captured the microbial community structure across participants.*

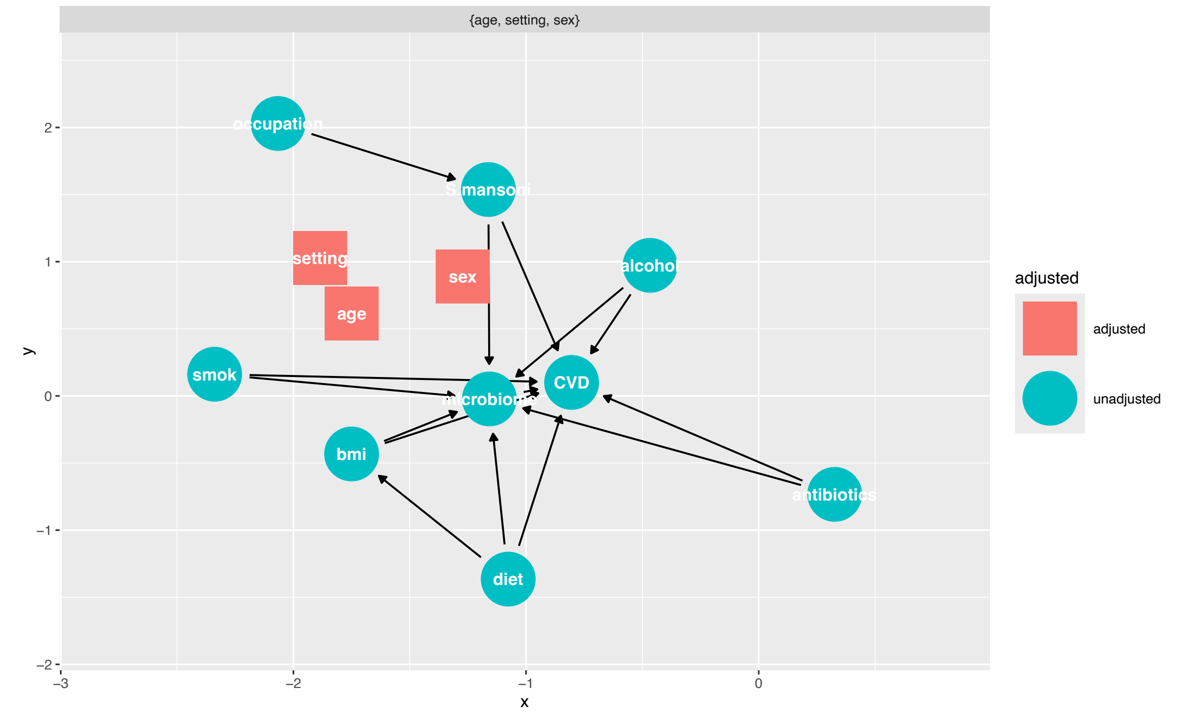

Supplementary Fig. 8: Directed acyclic graph (DAG) depicting the assumed causal structure linking S. mansoni infection, gut microbiome, and cardiometabolic risk.

Nodes represent measured variables, with arrows indicating presumed causal directions based on prior epidemiological and biological knowledge. S. mansoni infection acts as the primary exposure, influencing the gut microbiome and cardiometabolic risk factors (CVD), as well as intermediate lifestyle variables including body mass index (BMI), occupation, physical activity (exc), smoking, alcohol use, diet, antibiotic use. Age, *setting, and* sex *are treated as root causes with no incoming arrows and appear in red to denote the* ***minimal sufficient adjustment set*** *derived from the DAG using the adjustmentSets() function in* dagitty*. All other variables (in turquoise) are descendants of S. mansoni infection and therefore do not qualify as confounders of the exposure–mediator or exposure–outcome relationships. The DAG demonstrates that adjusting for age, setting and sex is sufficient to block all backdoor paths between S. mansoni infection, the microbiome, and cardiometabolic outcomes, forming the basis for the causal-identification strategy in the primary mediation analyses.*

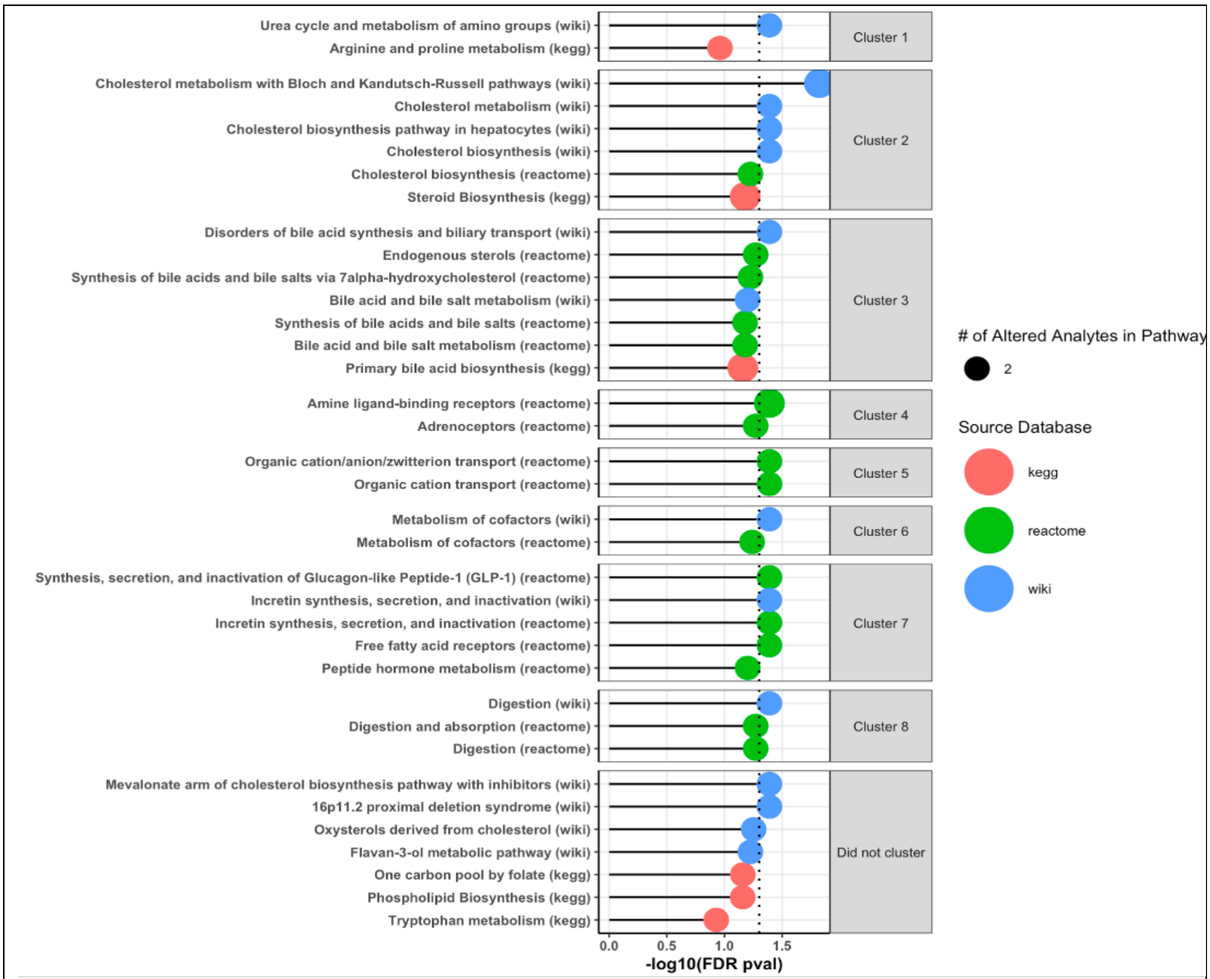

Supplementary Fig. 9: Total cholesterol-associated metabolic pathways.

Lollipop plot summarizing pathway enrichment results for metabolites associated

with total cholesterol. A pathway redundancy clustering algorithm, implemented via the RaMP *R package, was used to group highly overlapping pathways and identify functional redundancies. The vertical axis displays the names of enriched pathways, along with their assigned clusters (if applicable), the source database, the number of altered analytes per pathway, and the Fisher's exact test p-values corrected for multiple testing. Among 36 enriched pathways, 27 were grouped into 8 distinct clusters, reflecting shared biological functions or metabolite overlap.*

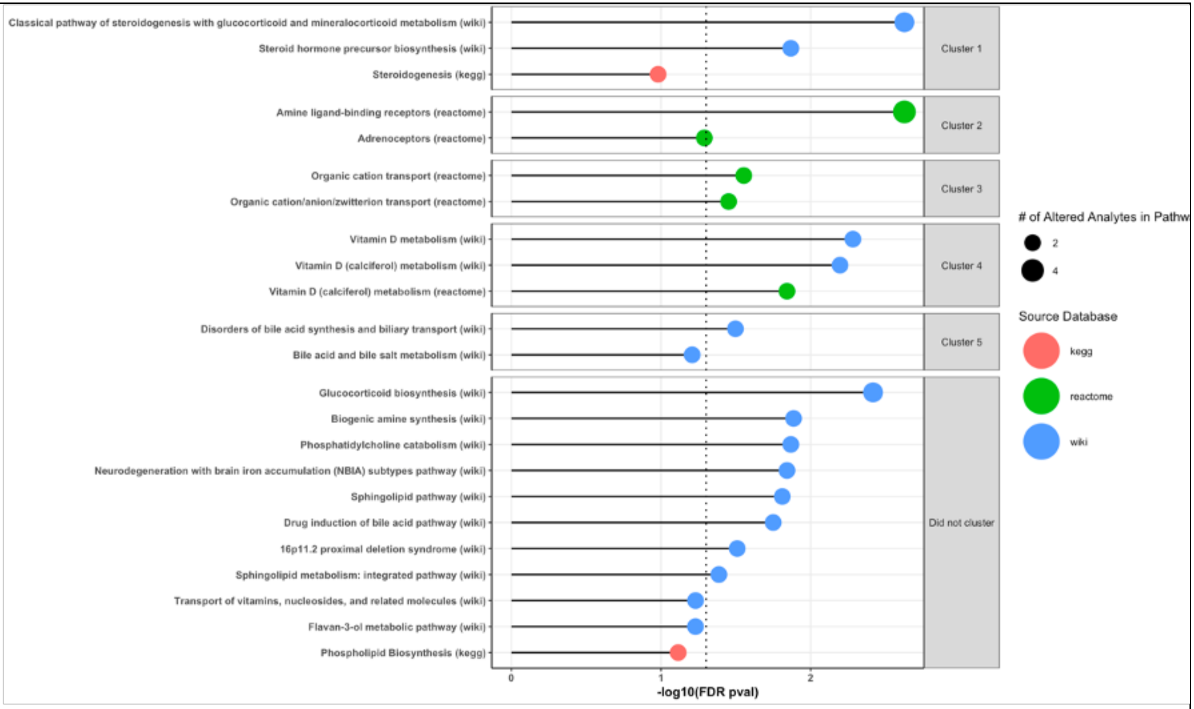

Supplementary Fig. 10: *Diastolic blood pressure-associated metabolic pathways.*

Lollipop plot displaying enriched metabolic pathways associated with diastolic blood pressure. A pathway redundancy clustering algorithm, implemented in the RaMP-DB package, was used to group highly overlapping pathways and identify functional redundancies within the enrichment results. The vertical axis indicates the names of enriched pathways, the cluster to which each pathway was assigned (if applicable), the source database, the number of altered analytes contributing to enrichment, and the Fisher's exact test p-values adjusted for multiple testing. Of

the pathways identified, 12 were grouped into 4 distinct clusters, while the remaining pathways could not be clustered due to lack of overlap.

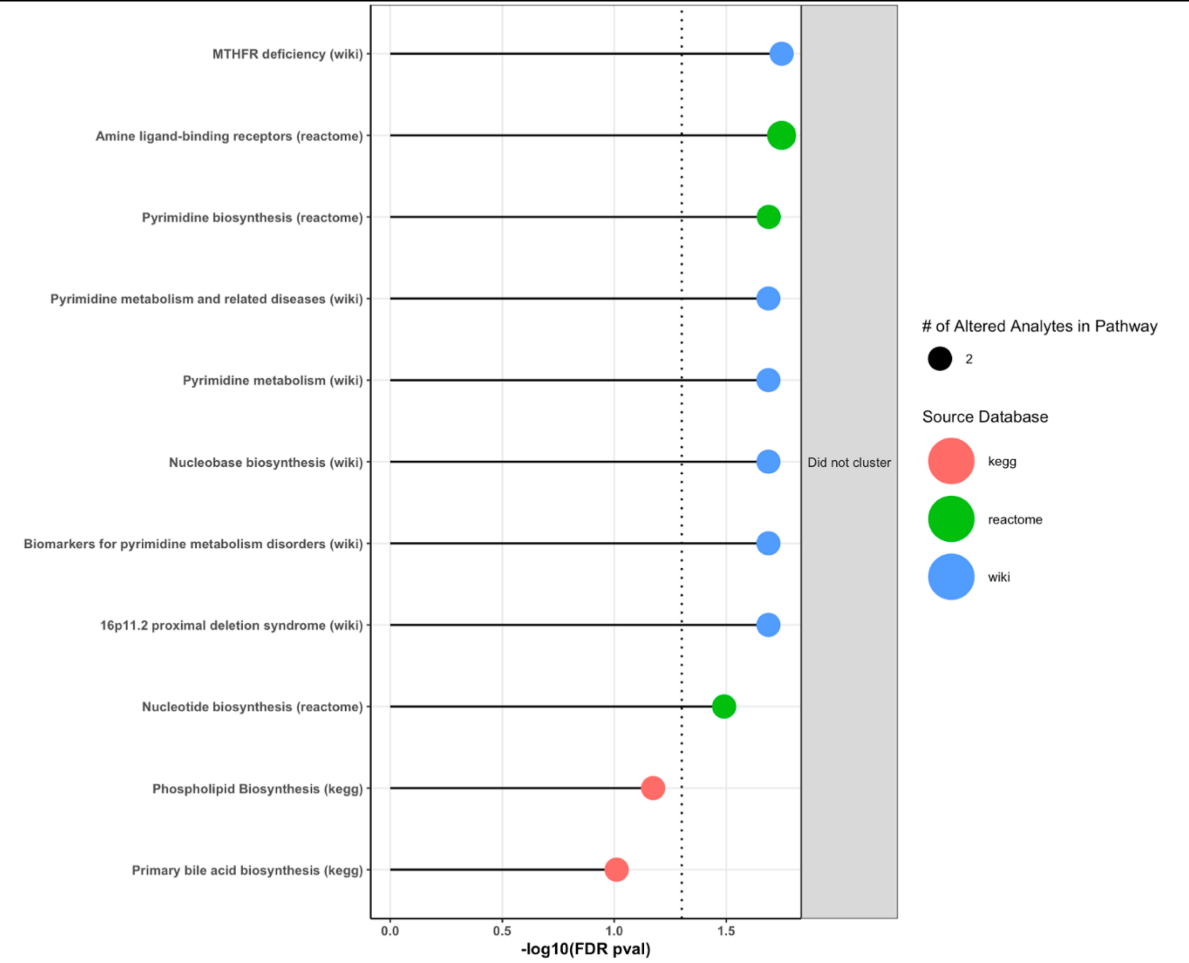

Supplementary Fig. 11: Insulin resistance-associated metabolic pathways. *Lollipop plot illustrating metabolic pathways enriched by metabolites associated with insulin resistance. A pathway redundancy clustering algorithm, implemented in the RaMP-DB package, was applied to group highly overlapping pathways and identify functional redundancies in the enrichment results. The vertical axis displays the names of enriched pathways, their corresponding clusters (if assigned), the source database, the number of altered analytes per pathway, and the Fisher's exact test p-values adjusted for multiple comparisons. In this analysis, 11 pathways were identified, none of which clustered due to limited overlap, indicating distinct biological processes.*

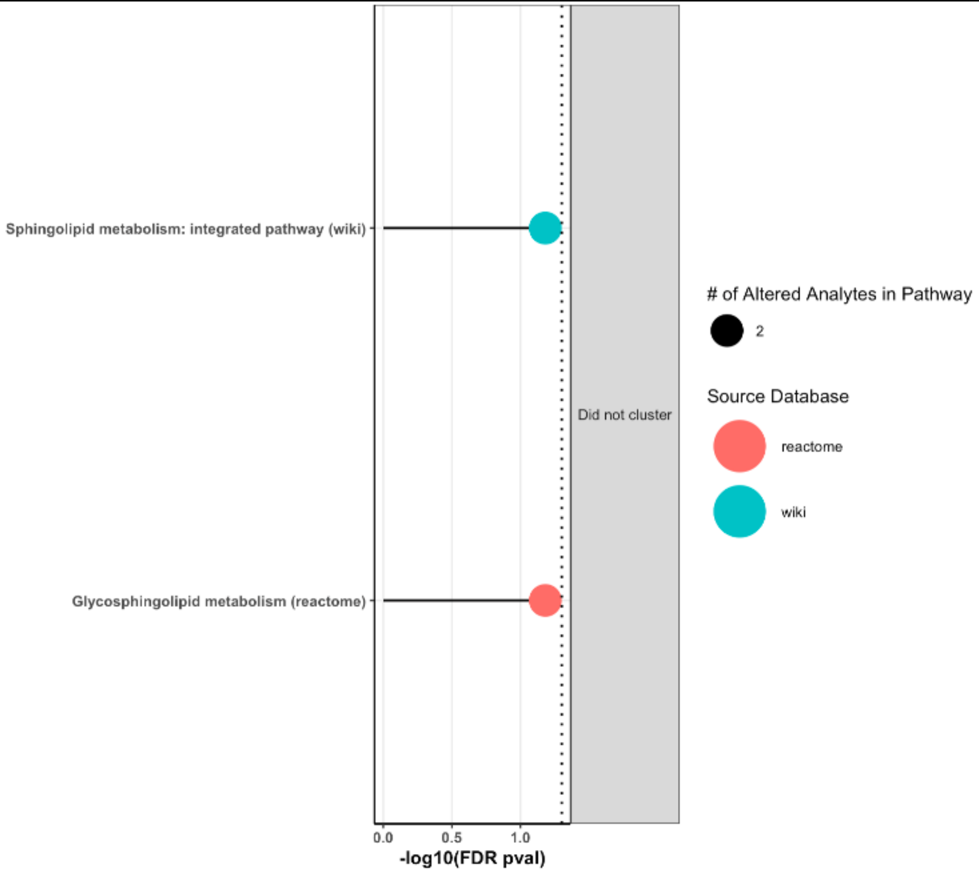

Supplementary Fig. 12: Systolic blood pressure-associated metabolic pathways.

Lollipop plot showing metabolic pathways enriched by metabolites associated with systolic blood pressure. A pathway redundancy clustering algorithm, implemented in the RaMP *R package, was used to group highly overlapping pathways and identify functional redundancies in the enrichment results. The vertical axis presents the names of enriched pathways, their assigned clusters (if any), the source database, the number of altered analytes contributing to each pathway, and Fisher's exact test p-values corrected for multiple testing. The two enriched pathways identified did not cluster, suggesting limited overlap and distinct biological roles*

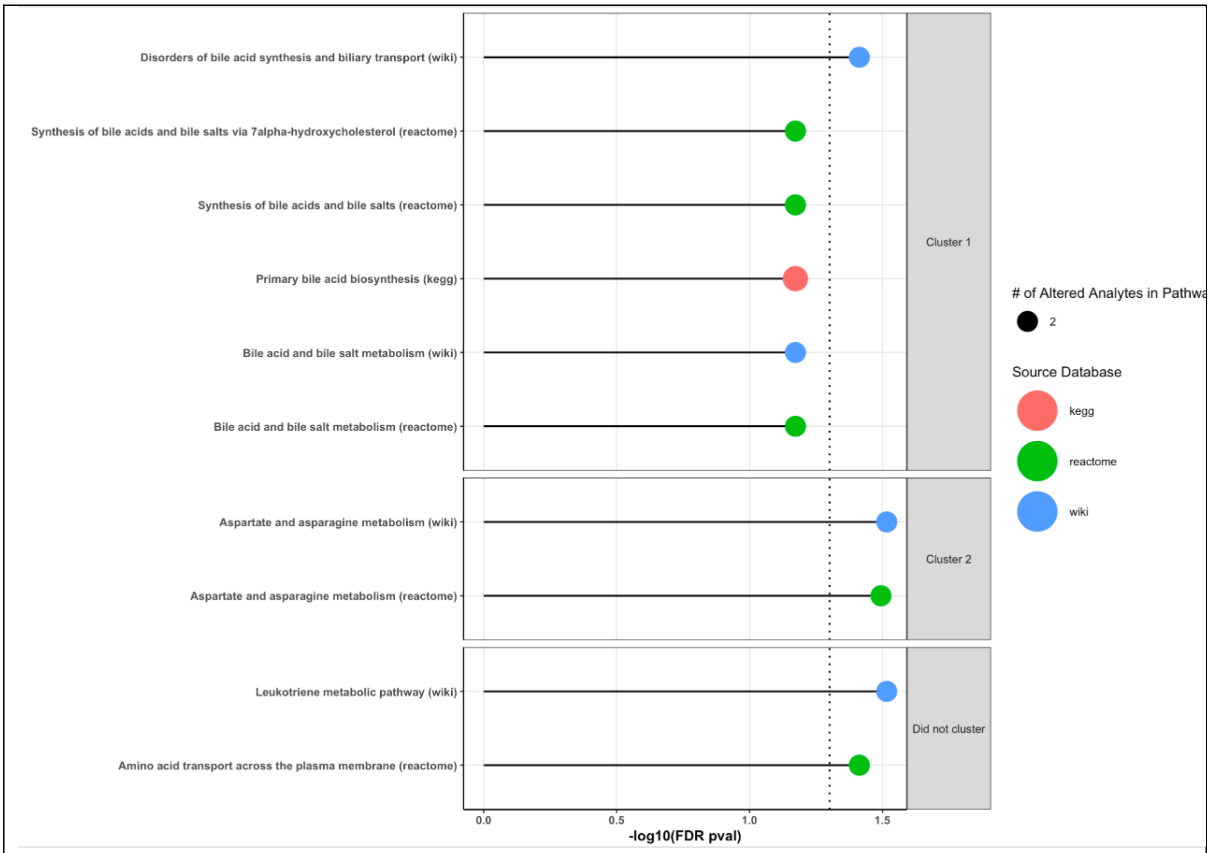

Supplementary Fig. 13: Glucose intolerance-associated metabolic pathways. *Lollipop plot illustrating metabolic pathways enriched by metabolites associated with glucose intolerance. A pathway redundancy clustering algorithm, implemented in the RaMP-DB package, was used to group highly overlapping pathways and identify functional redundancies in the enrichment results. The vertical axis displays the names of enriched pathways, their corresponding clusters (if assigned), the source database, the number of altered analytes per pathway, and the Fisher's exact test p-values adjusted for multiple testing. Of the 10 enriched pathways, 8 were grouped into two distinct clusters, while the remaining two pathways did not cluster due to limited overlap.*

Medication Category	Number of participants
Allergy medications	2
Herbal medicines	0
Antihypertensives	1
Antidiabetics	0
Antiretrovirals (ARVs)	11
Antibiotics	6
Other medications	2

Supplementary Table 1. Self-reported medication at enrolment.

Medication use was documented only in the rural questionnaire module; the most reported treatments were antiretrovirals (n=11) and recent antibiotics (n=6). Very few participants reported antihypertensive (n=1), allergy-related (n=2), or other medications (n=2). The small number of individuals reporting chronic disease or relevant medications suggests limited potential for confounding of the associations between rural–urban setting, the gut microbiome, and cardiometabolic traits

Supplementart table 2: Associations between gut microbial taxa and cardiovascular disease (CVD) risk factors in the rural cohort. Associations were assessed using ordinary least squares linear regression models fitted separately for each microbial taxon and CVD trait. Unstandardised regression coefficients (β) and standardised coefficients (β_std) are shown with 95% confidence intervals and exact two-sided *P* values. β coefficients and confidence intervals were derived from the *t* statistic of the regression model. Models were adjusted for age, sex (categorical), and body mass index (BMI). Standardised coefficients were obtained by z-scaling continuous variables to facilitate comparison of effect sizes across outcomes. Analyses were conducted separately by *Schistosoma mansoni* infection status, as indicated in the group column. No adjustment was made for multiple comparisons. Positive coefficients indicate higher levels of the CVD risk factor with increasing microbial abundance, while negative coefficients indicate inverse associations.

microbe	cvdrisk	beta	beta_lci	beta_uci	beta_std	beta_std_lci	beta_std_uci	pvalue	group	Significance
Prevotella	Insulin	2.36966943673939e-05	-0.0001391	0.00018652	0.03094421	-0.1816729	0.24356127	0.77274329	Infected	
Prevotella	Diastolic BP	7.14718432806814e-06	-0.0002727	0.00028694	0.00449291	-0.1713949	0.18038076	0.95956489	Infected	
Prevotella	Total Chol	1.5484858311992e-06	-2.75E-05	3.06007653429102e-05	0.01214484	-0.2157134	0.2400031	0.9157522	Infected	
Prevotella	LDL Chol	-5.23E-06	-3.09E-05	2.04321117805494e-05	-0.0455004	-0.2687508	0.17775	0.68598934	Infected	
Prevotella	Glucose	-3.86E-06	-1.93E-05	1.16079451310945e-05	-0.0557989	-0.2795076	0.16790984	0.62083576	Infected	
Prevotella	Systolic BP	0.00021947	-0.0001228	0.00056178	0.11099858	-0.062122	0.28411918	0.20553837	Infected	
Blautia	Insulin	-0.0001442	-0.0004853	0.00019688	-0.0857993	-0.2887322	0.11713362	0.40245395	Infected	
Blautia	Diastolic BP	9.39186583128415e-05	-0.0004942	0.00068206	0.02689896	-0.1415489	0.19534683	0.75136264	Infected	
Blautia	Total Chol	-5.05E-05	-0.0001105	9.56916992152567e-06	-0.1803138	-0.3948216	0.03419401	0.09822087	Infected	
Blautia	LDL Chol	-2.51E-05	-7.88E-05	2.86613903388414e-05	-0.0993801	-0.3123619	0.11360169	0.35571741	Infected	
Blautia	Glucose	4.69501496849543e-06	-2.79E-05	3.72592203738049e-05	0.03094204	-0.1836692	0.24555328	0.77481196	Infected	
Blautia	Systolic BP	-0.000291	-0.0010156	0.00043355	-0.0670588	-0.2340173	0.0998997	0.42629518	Infected	
Faecalibacterium	Insulin	0.00011684	-0.000267	0.00050073	0.06371898	-0.1456243	0.27306223	0.5462381	Infected	
Faecalibacterium	Diastolic BP	0.00018086	-0.0004788	0.00084049	0.04747984	-0.1256858	0.22064549	0.58666021	Infected	
Faecalibacterium	Total Chol	-5.48E-05	-0.0001223	1.26561029822328e-05	-0.1795999	-0.4006524	0.04145265	0.10978517	Infected	
Faecalibacterium	LDL Chol	-4.09E-05	-0.0001008	1.90922067900907e-05	-0.1484993	-0.3663605	0.06936178	0.17865528	Infected	
Faecalibacterium	Glucose	1.09273966808455e-05	-2.56E-05	4.74319362326052e-05	0.06600931	-0.1545043	0.28652291	0.55287733	Infected	
Faecalibacterium	Systolic BP	0.00035053	-0.0004626	0.00116371	0.07403383	-0.0977124	0.24578008	0.39335756	Infected	
Bifidobacterium	Insulin	2.87244765830847e-05	-0.0001158	0.00017329	0.04092091	-0.1650215	0.2468633	0.69344721	Infected	
Bifidobacterium	Diastolic BP	-4.16E-05	-0.0002899	0.00020678	-0.0285178	-0.1988441	0.1418086	0.73974166	Infected	

Bifidobacterium	Total Chol	-1.47E-05	-4.03E-05	1.0864214877903e-05	-0.1260085	-0.3449744	0.09295751	0.25538196	Infected	
Bifidobacterium	LDL Chol	-7.35E-06	-3.01E-05	1.5407317334782e-05	-0.0697693	-0.2857648	0.14622612	0.52200467	Infected	
Bifidobacterium	Glucose	-4.37E-06	-1.81E-05	9.35347694620732e-06	-0.0689669	-0.285537	0.14760308	0.52788388	Infected	
Bifidobacterium	Systolic BP	8.44351329025134e-05	-0.0002222	0.0003911	0.04658626	-0.122615	0.21578748	0.58510421	Infected	
Clostridium_sensu_stricto_1	Insulin	-8.88E-05	-0.0003335	0.00015584	-0.0746782	-0.2803432	0.1309868	0.47184812	Infected	
Clostridium_sensu_stricto_1	Diastolic BP	6.0666903342089e-05	-0.0003608	0.00048214	0.02454665	-0.1459856	0.19507886	0.77517005	Infected	
Clostridium_sensu_stricto_1	Total Chol	9.3903194841116e-06	-3.43E-05	5.3126807850614e-05	0.04740378	-0.1733848	0.26819233	0.67018903	Infected	
Clostridium_sensu_stricto_1	LDL Chol	-8.09E-07	-3.95E-05	3.79083790666253e-05	-0.0045278	-0.2213213	0.21226561	0.9669345	Infected	
Clostridium_sensu_stricto_1	Glucose	2.98580803584413e-06	-2.03E-05	2.63214864300758e-05	0.0277991	-0.1894657	0.24506385	0.79957097	Infected	
Clostridium_sensu_stricto_1	Systolic BP	0.00016526	-0.0003547	0.00068524	0.05379637	-0.1154681	0.22306085	0.52869416	Infected	
Subdoligranulum	Insulin	-0.0001775	-0.0006746	0.0003197	-0.0730862	-0.2778336	0.13166122	0.47935951	Infected	
Subdoligranulum	Diastolic BP	0.00033208	-0.0005213	0.00118546	0.06583586	-0.1033485	0.23502021	0.44078927	Infected	
Subdoligranulum	Total Chol	6.34143321790424e-06	-8.26E-05	9.52885946705299e-05	0.01568551	-0.204325	0.23569604	0.88747824	Infected	
Subdoligranulum	LDL Chol	3.38994742357261e-06	-7.53E-05	8.20430780306748e-05	0.00930071	-0.2064932	0.22509461	0.93182989	Infected	
Subdoligranulum	Glucose	-1.40E-05	-6.13E-05	3.3330586905771e-05	-0.0638253	-0.2797021	0.15205144	0.5577676	Infected	
Subdoligranulum	Systolic BP	0.0002287	-0.0008291	0.00128654	0.03647782	-0.1322471	0.20520276	0.66803225	Infected	
Romboutsia	Insulin	-0.0002079	-0.0005585	0.00014272	-0.1199129	-0.3221428	0.08231703	0.24134808	Infected	
Romboutsia	Diastolic BP	0.00020259	-0.0004033	0.00080849	0.05625057	-0.1119794	0.22448058	0.50752239	Infected	
Romboutsia	Total Chol	4.79437762720136e-05	-1.42E-05	0.0001101	0.16608456	-0.0492181	0.38138725	0.12862423	Infected	
Romboutsia	LDL Chol	1.66246923146128e-05	-3.90E-05	7.2286729826946e-05	0.06387963	-0.1499992	0.27775852	0.55376633	Infected	
Romboutsia	Glucose	-1.62E-05	-4.97E-05	1.72030815805152e-05	-0.1037205	-0.3173515	0.10991058	0.33668167	Infected	
Romboutsia	Systolic BP	6.69474340379628e-05	-0.0006841	0.000818	0.01495476	-0.1528158	0.18272528	0.85958428	Infected	
Collinsella	Insulin	-9.51E-05	-0.0003991	0.00020899	-0.0635516	-0.2668309	0.13972776	0.53543307	Infected	
Collinsella	Diastolic BP	3.59715960565001e-05	-0.0004875	0.00055943	0.01157751	-0.1568988	0.18005385	0.89151693	Infected	
Collinsella	Total Chol	-7.68E-06	-6.20E-05	4.66564413104365e-05	-0.0308311	-0.2490146	0.18735241	0.77917332	Infected	
Collinsella	LDL Chol	1.35464565035573e-05	-3.44E-05	6.15148709012313e-05	0.0603372	-0.1533187	0.27399307	0.57551953	Infected	
Collinsella	Glucose	1.00112055370122e-06	-2.80E-05	2.99831488459687e-05	0.0074143	-0.2072267	0.22205529	0.94534025	Infected	
Collinsella	Systolic BP	0.00041557	-0.0002247	0.00105588	0.10760682	-0.0581939	0.27340757	0.20010096	Infected	

Agathobacter	Insulin	7.63322473951934e-05	-0.0002462	0.00039882	0.05063638	-0.1632925	0.26456523	0.63874172	Infected	
Agathobacter	Diastolic BP	-0.0003428	-0.000892	0.00020641	-0.1094701	-0.2848541	0.06591386	0.2176801	Infected	
Agathobacter	Total Chol	-2.65E-05	-8.38E-05	3.07983041015209e-05	-0.105525	-0.3337585	0.12270841	0.36009934	Infected	
Agathobacter	LDL Chol	-2.59E-05	-7.65E-05	2.46623624101359e-05	-0.1145708	-0.3381334	0.10899184	0.31069982	Infected	
Agathobacter	Glucose	2.31023859292962e-05	-7.15E-06	5.33597362829581e-05	0.16976204	-0.0525764	0.39210052	0.13251095	Infected	
Agathobacter	Systolic BP	-0.0008208	-0.0014808	-0.0001608	-0.210878	-0.3804351	-0.0413209	0.01546071	Infected	*
Catenibacterium	Insulin	5.55618624535283e-05	-0.0002905	0.00040166	0.03321631	-0.1736906	0.24012319	0.7500835	Infected	
Catenibacterium	Diastolic BP	-0.0001934	-0.0007866	0.00039978	-0.0556673	-0.2263877	0.11505318	0.51808067	Infected	
Catenibacterium	Total Chol	-2.17E-05	-8.33E-05	3.98757050561941e-05	-0.0778977	-0.2989733	0.14317787	0.48502279	Infected	
Catenibacterium	LDL Chol	-3.03E-05	-8.45E-05	2.38853676232769e-05	-0.1206538	-0.3364363	0.09512863	0.26899978	Infected	
Catenibacterium	Glucose	-1.93E-05	-5.20E-05	1.33021647322135e-05	-0.1280406	-0.3441711	0.08808989	0.24176565	Infected	
Catenibacterium	Systolic BP	0.00020338	-0.0005306	0.0009373	0.04708871	-0.1228408	0.21701818	0.58268933	Infected	
UCG.002	Insulin	-2.06E-06	-0.0005211	0.00051695	-0.0008065	-0.2039695	0.20235651	0.99371364	Infected	
UCG.002	Diastolic BP	-0.0002008	-0.001091	0.00068947	-0.0378361	-0.2055947	0.12992262	0.6546176	Infected	
UCG.002	Total Chol	2.98153021508861e-05	-6.25E-05	0.00012213	0.07009747	-0.1469442	0.28713919	0.52206273	Infected	
UCG.002	LDL Chol	4.72668200261424e-05	-3.39E-05	0.00012841	0.1232626	-0.0883318	0.33485704	0.24963749	Infected	
UCG.002	Glucose	8.33185509991327e-06	-4.10E-05	5.76457163901475e-05	0.03612776	-0.1777021	0.24995764	0.73745903	Infected	
UCG.002	Systolic BP	-0.0007321	-0.0018215	0.00035736	-0.1109871	-0.2761514	0.0541771	0.18480731	Infected	
Streptococcus	Insulin	5.79880809289777e-05	-0.0001814	0.00029736	0.0500978	-0.1567064	0.256902	0.63090704	Infected	
Streptococcus	Diastolic BP	3.92883787452971e-05	-0.0003724	0.00045095	0.01633973	-0.1548671	0.18754654	0.84977604	Infected	
Streptococcus	Total Chol	4.70481618012366e-05	5.64594178604866e-06	8.84503818164246e-05	0.24412684	0.02929606	0.45895761	0.02646633	Infected	*
Streptococcus	LDL Chol	2.83546013192902e-05	-8.90E-06	6.56081981165966e-05	0.16319551	-0.0512184	0.37760938	0.13371736	Infected	
Streptococcus	Glucose	1.03796709187014e-05	-1.23E-05	3.30531001139243e-05	0.0993328	-0.1176505	0.31631609	0.36483679	Infected	
Streptococcus	Systolic BP	-0.0001776	-0.000685	0.00032988	-0.0594146	-0.2292047	0.11037551	0.48802695	Infected	
Holdemanella	Insulin	4.88025736787232e-05	-0.0004874	0.00058501	0.0190517	-0.1902738	0.22837715	0.85666054	Infected	
Holdemanella	Diastolic BP	0.0003857	-0.0005313	0.00130268	0.07248368	-0.0998434	0.24481079	0.40487285	Infected	
Holdemanella	Total Chol	1.4437421651005e-06	-9.42E-05	9.70948311331923e-05	0.00338512	-0.2208862	0.22765647	0.97610042	Infected	
Holdemanella	LDL Chol	7.43037167277111e-06	-7.71E-05	9.19882352475494e-05	0.01932441	-0.200588	0.23923679	0.86155626	Infected	

Holdemanella	Glucose	2.566699547567 09e-07	-5.07E-05	5.125275607384 28e-05	0.00110993	-0.2194146	0.22163448	0.99202946	Infected	
Holdemanella	Systolic BP	0.00039348	-0.0007418	0.00152876	0.0594908	-0.112157	0.23113863	0.49218007	Infected	
Dorea	Insulin	0.00065629	-0.0002267	0.00153929	0.14964179	-0.0516938	0.35097733	0.14295558	Infected	
Dorea	Diastolic BP	0.00054584	-0.0009872	0.00207885	0.05991314	-0.1083568	0.22818311	0.48046972	Infected	
Dorea	Total Chol	-8.29E-05	-0.0002415	7.564622883831 73e-05	-0.1135928	-0.3307806	0.10359509	0.30092189	Infected	
Dorea	LDL Chol	-9.16E-06	-0.0001504	0.00013204	-0.0139095	-0.2283889	0.20056996	0.89758583	Infected	
Dorea	Glucose	2.601735402567 13e-05	-5.89E-05	0.00011096	0.06571299	-0.1488265	0.28025253	0.54371264	Infected	
Dorea	Systolic BP	-0.0006485	-0.0025442	0.00124723	-0.0572648	-0.2246703	0.11014058	0.49781754	Infected	
Bacteroides	Insulin	-0.0001022	-0.0005026	0.00029805	-0.0518797	-0.2549892	0.15122983	0.6124726	Infected	
Bacteroides	Diastolic BP	-0.0003238	-0.0010085	0.000361	-0.0790789	-0.2463349	0.08817698	0.34940884	Infected	
Bacteroides	Total Chol	4.440036626182 59e-05	-2.64E-05	0.0001152	0.13530743	-0.0804504	0.35106523	0.2155331	Infected	
Bacteroides	LDL Chol	4.750066207663 16e-05	-1.48E-05	0.00010981	0.1605634	-0.0500452	0.37117201	0.13308731	Infected	
Bacteroides	Glucose	4.789136744684 51e-06	-3.33E-05	4.290039798895 12e-05	0.02691712	-0.1872854	0.24111968	0.80307979	Infected	
Bacteroides	Systolic BP	-0.0002676	-0.0011169	0.00058163	-0.0525884	-0.219472	0.11429526	0.53219831	Infected	
Escherichia.Shig ella	Insulin	-0.0004718	-0.00108	0.00013636	-0.1564993	-0.3582298	0.04523118	0.12649886	Infected	
Escherichia.Shig ella	Diastolic BP	0.00024055	-0.0008187	0.00129976	0.03841091	-0.1307233	0.20754511	0.65238182	Infected	
Escherichia.Shig ella	Total Chol	-4.27E-05	-0.0001525	6.695772263250 61e-05	-0.0851641	-0.3037235	0.13339538	0.44017651	Infected	
Escherichia.Shig ella	LDL Chol	-1.59E-05	-0.0001133	8.136654993750 1e-05	-0.0352372	-0.2502778	0.17980336	0.74508779	Infected	
Escherichia.Shig ella	Glucose	-1.38E-05	-7.24E-05	4.487740794818 53e-05	-0.0505466	-0.265987	0.16489371	0.64168458	Infected	
Escherichia.Shig ella	Systolic BP	0.00087053	-0.0004257	0.00216678	0.1118336	-0.0546902	0.27835738	0.18506786	Infected	
Christensenellac eae_R.7_group	Insulin	-7.72E-05	-0.0010906	0.00093629	-0.0161454	-0.228171	0.19588019	0.87987473	Infected	
Christensenellac eae_R.7_group	Diastolic BP	-0.0004526	-0.0021905	0.00128526	-0.0455864	-0.220613	0.12944027	0.60550599	Infected	
Christensenellac eae_R.7_group	Total Chol	-2.79E-05	-0.0002085	0.0001528	-0.0350114	-0.2620235	0.19200074	0.75959254	Infected	
Christensenellac eae_R.7_group	LDL Chol	9.765621030046 49e-05	-6.06E-05	0.00025595	0.13610808	-0.0845196	0.35673576	0.22302683	Infected	
Christensenellac eae_R.7_group	Glucose	-2.04E-06	-9.84E-05	9.433604416758 55e-05	-0.0047343	-0.2280868	0.2186181	0.96644218	Infected	
Christensenellac eae_R.7_group	Systolic BP	-0.0014562	-0.003583	0.00067053	-0.1179902	-0.2903101	0.05432982	0.17671626	Infected	
Eubacterium_hal lii_group	Insulin	-0.0002337	-0.0016389	0.00117146	-0.0345938	-0.2425772	0.17338952	0.74138928	Infected	
Eubacterium_hal lii_group	Diastolic BP	0.00054153	-0.0018705	0.00295359	0.03858494	-0.1332784	0.21044824	0.65608994	Infected	

Eubacterium_hallii_group	Total Chol	-0.0001463	-0.0003948	0.00010233	-0.130014	-0.3509991	0.09097115	0.24499788	Infected	
Eubacterium_hallii_group	LDL Chol	-8.84E-05	-0.0003092	0.00013247	-0.0871381	-0.304897	0.13062083	0.42800724	Infected	
Eubacterium_hallii_group	Glucose	2.42642973524652e-05	-0.0001093	0.00015786	0.03978246	-0.1792517	0.25881658	0.71859284	Infected	
Eubacterium_hallii_group	Systolic BP	-0.0014953	-0.0044618	0.00147121	-0.0857148	-0.2557653	0.08433564	0.31866595	Infected	
Muribaculaceae	Insulin	-4.00E-05	-0.0005079	0.00042797	-0.0183161	-0.232768	0.19613578	0.86540042	Infected	
Muribaculaceae	Diastolic BP	0.00037907	-0.0004202	0.0011783	0.08363118	-0.0926958	0.25995814	0.3478969	Infected	
Muribaculaceae	Total Chol	1.67287280896093e-06	-8.18E-05	8.51428952083606e-05	0.0046047	-0.2251522	0.23436164	0.96826983	Infected	
Muribaculaceae	LDL Chol	1.34062031608668e-05	-6.03E-05	8.71479843195501e-05	0.04093131	-0.1842143	0.26607695	0.71833719	Infected	
Muribaculaceae	Glucose	-1.93E-05	-6.35E-05	2.5034112060575e-05	-0.0977397	-0.322568	0.12708858	0.3893664	Infected	
Muribaculaceae	Systolic BP	0.00010523	-0.0008883	0.00109872	0.01867812	-0.157661	0.19501727	0.83350939	Infected	
Dialister	Insulin	0.00014985	-0.0003478	0.00064751	0.06160707	-0.1429919	0.26620607	0.55054033	Infected	
Dialister	Diastolic BP	0.00043909	-0.0004118	0.00129002	0.08690092	-0.0815084	0.25531025	0.30739856	Infected	
Dialister	Total Chol	3.19993305224378e-05	-5.67E-05	0.00012067	0.07901396	-0.1399261	0.29795399	0.47454398	Infected	
Dialister	LDL Chol	-1.78E-05	-9.64E-05	6.07444246294755e-05	-0.048788	-0.2639483	0.16637234	0.65288374	Infected	
Dialister	Glucose	3.77531551373079e-06	-4.36E-05	5.11981582491779e-05	0.01719303	-0.1987736	0.2331597	0.87445969	Infected	
Dialister	Systolic BP	0.0008397	-0.0002022	0.00188159	0.13370073	-0.0321944	0.29959581	0.11262684	Infected	
uncultured	Insulin	0.00012508	-0.0009685	0.0012187	0.02393578	-0.1853428	0.23321433	0.82044787	Infected	
uncultured	Diastolic BP	0.00065572	-0.0012174	0.00252879	0.06040529	-0.112144	0.23295458	0.48784612	Infected	
uncultured	Total Chol	2.96618531816844e-05	-0.0001653	0.00022466	0.03409173	-0.1900249	0.25820835	0.76278158	Infected	
uncultured	LDL Chol	4.82516063128208e-05	-0.0001239	0.00022042	0.06151405	-0.1579765	0.28100459	0.57841862	Infected	
uncultured	Glucose	2.68404534900872e-05	-7.70E-05	0.00013068	0.05689536	-0.1632288	0.27701957	0.60825212	Infected	
uncultured	Systolic BP	0.00164991	-0.0006426	0.00394246	0.1222811	-0.0476285	0.29219072	0.155885	Infected	
Succinivibrio	Insulin	6.6661689770677e-05	-0.0003849	0.00051823	0.0301848	-0.1742884	0.234658	0.76958435	Infected	
Succinivibrio	Diastolic BP	0.00086012	0.00010904	0.00161119	0.18748652	0.02376798	0.35120506	0.0253548	Infected	*
Succinivibrio	Total Chol	7.56626467584619e-05	-3.07E-06	0.00015439	0.20577208	-0.0083456	0.41988972	0.05938253	Infected	
Succinivibrio	LDL Chol	8.71685050420783e-06	-6.25E-05	7.9939342085894e-05	0.02629519	-0.1885541	0.24114446	0.80810538	Infected	
Succinivibrio	Glucose	-1.73E-05	-6.01E-05	2.55087735876846e-05	-0.0866417	-0.3012306	0.12794718	0.42388229	Infected	
Succinivibrio	Systolic BP	0.00081804	-0.0001232	0.00175929	0.14345977	-0.0216058	0.30852536	0.08752639	Infected	

UCG.005	Insulin	0.00019824	-0.001273	0.00166951	0.02766452	-0.1776479	0.23297697	0.78917929	Infected	
UCG.005	Diastolic BP	-0.0005956	-0.0031201	0.00192892	-0.0400118	-0.2096036	0.12958007	0.63982833	Infected	
UCG.005	Total Chol	0.00020612	-5.22E-05	0.00046444	0.17276108	-0.0437472	0.38926934	0.11617956	Infected	
UCG.005	LDL Chol	0.00026386	3.959643617302 51e-05	0.00048812	0.24530257	0.0368116	0.45379353	0.02172162	Infected	*
UCG.005	Glucose	9.553002292083 42e-05	-4.27E-05	0.0002338	0.14767022	-0.0660708	0.36141129	0.1728954	Infected	
UCG.005	Systolic BP	-0.001594	-0.0046985	0.00151044	-0.0861513	-0.2539362	0.0816335	0.30977621	Infected	
Clostridia_UCG. 014	Insulin	-0.0003919	-0.0013553	0.00057145	-0.0829497	-0.286853	0.1209536	0.42040202	Infected	
Clostridia_UCG. 014	Diastolic BP	0.00023072	-0.0014301	0.00189153	0.02350839	-0.1457147	0.19273145	0.7828099	Infected	
Clostridia_UCG. 014	Total Chol	-6.00E-06	-0.0001785	0.00016654	-0.0076266	-0.2269652	0.21171203	0.94497989	Infected	
Clostridia_UCG. 014	LDL Chol	8.130963627677 67e-05	-7.01E-05	0.00023275	0.11465288	-0.0988917	0.32819749	0.28835948	Infected	
Clostridia_UCG. 014	Glucose	-2.59E-06	-9.46E-05	8.939763045021 46e-05	-0.0060744	-0.2217498	0.20960101	0.95542103	Infected	
Clostridia_UCG. 014	Systolic BP	0.00168874	-0.0003294	0.00370691	0.13843366	-0.0270055	0.30387277	0.0997345	Infected	
Intestinibacter	Insulin	-0.0001262	-0.0010024	0.00075002	-0.0298428	-0.2370604	0.17737469	0.77505534	Infected	
Intestinibacter	Diastolic BP	0.00024881	-0.0012558	0.00175348	0.02832685	-0.1429746	0.1996283	0.74283887	Infected	
Intestinibacter	Total Chol	8.248954266684 21e-05	-7.27E-05	0.00023772	0.11716908	-0.103319	0.33765711	0.29328711	Infected	
Intestinibacter	LDL Chol	2.235808793309 92e-05	-0.0001158	0.00016051	0.03522578	-0.1824384	0.25288997	0.74813247	Infected	
Intestinibacter	Glucose	3.768188793444 45e-05	-4.52E-05	0.0001206	0.09871496	-0.118507	0.31593696	0.36833517	Infected	
Intestinibacter	Systolic BP	0.00013907	-0.0017221	0.00200029	0.01273828	-0.1577359	0.18321246	0.88210693	Infected	
Ruminococcus	Insulin	0.00014855	-0.0008303	0.00112744	0.03168136	-0.1770908	0.24045348	0.7633336	Infected	
Ruminococcus	Diastolic BP	0.00078126	-0.0008917	0.00245418	0.08021137	-0.0915451	0.2519678	0.35531429	Infected	
Ruminococcus	Total Chol	-4.79E-05	-0.0002222	0.00012649	-0.0613076	-0.2846394	0.16202421	0.58621623	Infected	
Ruminococcus	LDL Chol	-4.13E-05	-0.0001954	0.0001129	-0.0586426	-0.2776967	0.16041141	0.59551633	Infected	
Ruminococcus	Glucose	8.461876005821 81e-05	-6.51E-06	0.00017575	0.19990934	-0.0153885	0.4152072	0.06830648	Infected	
Ruminococcus	Systolic BP	-0.0003276	-0.002406	0.00175086	-0.0270575	-0.1987357	0.14462061	0.75449585	Infected	
Sarcina	Insulin	0.00076735	-0.0001178	0.00165254	0.17719729	-0.0272105	0.38160507	0.08832541	Infected	
Sarcina	Diastolic BP	0.00062206	-0.000921	0.0021651	0.06915003	-0.1023802	0.24068021	0.42459197	Infected	
Sarcina	Total Chol	6.288612160001 98e-05	-9.74E-05	0.00022315	0.08721854	-0.1350555	0.30949259	0.43699125	Infected	
Sarcina	LDL Chol	3.153651728752 31e-05	-0.0001105	0.00017362	0.04851539	-0.170066	0.26709676	0.65974951	Infected	

Sarcina	Glucose	3.851938419322 29e-07	-8.54E-05	8.616678511992 99e-05	0.0009853	-0.2184388	0.22040941	0.99288891	Infected	
Sarcina	Systolic BP	0.00085968	-0.0010459	0.00276531	0.07688452	-0.0935434	0.24731248	0.37182233	Infected	
Eubacterium_co prostanoligenes_ group	Insulin	0.00035825	-0.0012073	0.00192376	0.04766766	-0.1606371	0.25597246	0.64990911	Infected	
Eubacterium_co prostanoligenes_ group	Diastolic BP	-0.0004174	-0.0031082	0.00227338	-0.0267365	-0.1990914	0.14561827	0.75823686	Infected	
Eubacterium_co prostanoligenes_ group	Total Chol	3.508776719433 44e-05	-0.0002444	0.00031456	0.02804088	-0.1953007	0.25138245	0.80324924	Infected	
Eubacterium_co prostanoligenes_ group	LDL Chol	0.00020217	-4.07E-05	0.00044508	0.17921219	-0.0361129	0.39453726	0.1015292	Infected	
Eubacterium_co prostanoligenes_ group	Glucose	-1.07E-08	-0.0001491	0.00014905	-1.58E-05	-0.2197141	0.21968244	0.99988586	Infected	
Eubacterium_co prostanoligenes_ group	Systolic BP	0.0008693	-0.0024535	0.00419205	0.04479733	-0.1264338	0.21602848	0.60389655	Infected	
Lactobacillus	Insulin	0.00048781	-0.0001771	0.00115267	0.14736189	-0.0534876	0.34821139	0.14809257	Infected	
Lactobacillus	Diastolic BP	-0.0012887	-0.0024088	-0.0001686	-0.1874097	-0.3503009	-0.0245186	0.02470049	Infected	*
Lactobacillus	Total Chol	-0.0001114	-0.0002289	6.107996249998 23e-06	-0.2021464	-0.4153751	0.01108228	0.0628256	Infected	
Lactobacillus	LDL Chol	-8.82E-05	-0.0001926	1.615056659053 14e-05	-0.1775759	-0.3876553	0.03250349	0.09639329	Infected	
Lactobacillus	Glucose	4.623515298979 25e-06	-5.95E-05	6.870401249794 78e-05	0.01547173	-0.1989617	0.22990518	0.88613455	Infected	
Lactobacillus	Systolic BP	-0.0008879	-0.0023048	0.00052907	-0.1038771	-0.2696541	0.06189984	0.2159092	Infected	
Methanobreviba cter	Insulin	4.909840166930 5e-06	-0.000389	0.00039882	0.002567	-0.2033801	0.20851407	0.98026294	Infected	
Methanobreviba cter	Diastolic BP	-0.00018	-0.0008554	0.00049529	-0.0453129	-0.2152829	0.12465716	0.59704704	Infected	
Methanobreviba cter	Total Chol	-3.04E-05	-0.0001003	3.952922760427 95e-05	-0.0954136	-0.3149554	0.12412819	0.38950436	Infected	
Methanobreviba cter	LDL Chol	-9.94E-06	-7.20E-05	5.213735787302 24e-05	-0.0346194	-0.2508376	0.18159877	0.75072135	Infected	
Methanobreviba cter	Glucose	-2.51E-05	-6.22E-05	1.188006186119 75e-05	-0.1455867	-0.3599763	0.06880294	0.1802659	Infected	
Methanobreviba cter	Systolic BP	6.348225907330 18e-05	-0.0007728	0.00089977	0.01285443	-0.1564841	0.18219292	0.88024834	Infected	
Roseburia	Insulin	0.00034684	-0.0007614	0.00145511	0.0655196	-0.1438399	0.27487913	0.53501553	Infected	
Roseburia	Diastolic BP	-0.0005964	-0.0024999	0.00130707	-0.0542379	-0.2273384	0.11886267	0.53452235	Infected	
Roseburia	Total Chol	-3.29E-05	-0.0002309	0.00016516	-0.0372814	-0.2619484	0.18738549	0.74197203	Infected	
Roseburia	LDL Chol	-8.57E-05	-0.0002598	8.842596856137 15e-05	-0.1078528	-0.3269892	0.11128367	0.330136	Infected	
Roseburia	Glucose	5.177935096579 96e-05	-5.32E-05	0.00015677	0.10835093	-0.1113464	0.3280483	0.32914984	Infected	

Roseburia	Systolic BP	-0.0009715	-0.0033203	0.00137737	-0.0710759	-0.2429235	0.10077164	0.41272172	Infected	
Ruminococcus_torques_group	Insulin	0.00049932	-0.001284	0.00228259	0.05741533	-0.1476402	0.26247083	0.57876959	Infected	
Ruminococcus_torques_group	Diastolic BP	-0.001726	-0.0047699	0.00131797	-0.09554	-0.2640357	0.07295577	0.26237135	Infected	
Ruminococcus_torques_group	Total Chol	-9.28E-05	-0.0004108	0.00022515	-0.0641185	-0.283731	0.15549393	0.5626886	Infected	
Ruminococcus_torques_group	LDL Chol	-6.37E-05	-0.0003451	0.00021767	-0.0488254	-0.2643986	0.16674782	0.65325614	Infected	
Ruminococcus_torques_group	Glucose	9.94501441548313e-05	-6.89E-05	0.00026785	0.12667404	-0.0878242	0.34117227	0.24323594	Infected	
Ruminococcus_torques_group	Systolic BP	-0.0011249	-0.0049104	0.0026607	-0.0500945	-0.2186815	0.11849248	0.55579284	Infected	
Treponema	Insulin	-5.30E-05	-0.0003814	0.0002754	-0.0338081	-0.2432759	0.17565981	0.74878722	Infected	
Treponema	Diastolic BP	-0.0001948	-0.0007575	0.00036791	-0.0598096	-0.2325847	0.11296536	0.49269777	Infected	
Treponema	Total Chol	1.56229960055772e-05	-4.29E-05	7.41254302859083e-05	0.05985006	-0.1642666	0.28396676	0.59642188	Infected	
Treponema	LDL Chol	2.90241006467884e-05	-2.24E-05	8.04262915678644e-05	0.1233306	-0.0950901	0.34175125	0.26435489	Infected	
Treponema	Glucose	-2.58E-05	-5.65E-05	4.93559207486916e-06	-0.182006	-0.398884	0.03487191	0.09876579	Infected	
Treponema	Systolic BP	-0.0001905	-0.0008869	0.00050599	-0.047048	-0.2190908	0.12499486	0.58764234	Infected	
Terrisporobacter	Insulin	-0.0005427	-0.0015257	0.00044022	-0.1120671	-0.3150342	0.09089998	0.27499326	Infected	
Terrisporobacter	Diastolic BP	0.00051043	-0.0011871	0.00220795	0.05073764	-0.1179984	0.21947364	0.55109364	Infected	
Terrisporobacter	Total Chol	-7.45E-05	-0.0002504	0.00010133	-0.0924404	-0.3105478	0.12566701	0.4013101	Infected	
Terrisporobacter	LDL Chol	-5.46E-05	-0.0002103	0.00010112	-0.075117	-0.2893364	0.13910248	0.48712927	Infected	
Terrisporobacter	Glucose	1.17481147273636e-05	-8.24E-05	0.00010591	0.02687138	-0.1884944	0.24223717	0.80444728	Infected	
Terrisporobacter	Systolic BP	-0.0008687	-0.0029629	0.00122548	-0.0694735	-0.2369505	0.09800351	0.4113447	Infected	
Senegalimassilia	Insulin	-0.0004612	-0.0017148	0.00079238	-0.0788965	-0.2933467	0.13555368	0.46603571	Infected	
Senegalimassilia	Diastolic BP	0.00109425	-0.001052	0.00324049	0.09011389	-0.0866346	0.26686234	0.31317746	Infected	
Senegalimassilia	Total Chol	6.67214662828077e-05	-0.0001571	0.00029056	0.06855431	-0.1614337	0.29854228	0.5545536	Infected	
Senegalimassilia	LDL Chol	0.00014084	-5.49E-05	0.00033661	0.16050772	-0.0626158	0.38363119	0.15606435	Infected	
Senegalimassilia	Glucose	1.57817464073014e-05	-0.0001038	0.00013534	0.02990615	-0.1966535	0.25646577	0.79336975	Infected	
Senegalimassilia	Systolic BP	0.00196242	-0.0006712	0.00459607	0.13001954	-0.0444727	0.30451176	0.14195853	Infected	
Weissella	Insulin	0.00017256	-0.0003164	0.00066154	0.07233669	-0.1326435	0.2773169	0.48435825	Infected	
Weissella	Diastolic BP	-2.71E-06	-0.0008452	0.00083983	-0.0005467	-0.1705699	0.16947653	0.99490818	Infected	
Weissella	Total Chol	-1.72E-05	-0.0001046	7.02037320638831e-05	-0.0432996	-0.2633535	0.17675441	0.69627717	Infected	

Weissella	LDL Chol	-1.42E-05	-9.15E-05	6.304760613605 59e-05	-0.039773	-0.255618	0.17607206	0.7146843	Infected	
Weissella	Glucose	-5.53E-06	-5.22E-05	4.109816702953 04e-05	-0.025674	-0.2421878	0.1908397	0.81396541	Infected	
Weissella	Systolic BP	0.00054721	-0.000487	0.00158139	0.08884079	-0.0790607	0.25674225	0.29535124	Infected	
CAG.352	Insulin	-0.0001355	-0.0007485	0.00047759	-0.0459669	-0.2539775	0.16204375	0.66114529	Infected	
CAG.352	Diastolic BP	-0.000177	-0.0012305	0.00087658	-0.0289025	-0.2009799	0.14317495	0.73894534	Infected	
CAG.352	Total Chol	-5.20E-07	-0.00011	0.00010896	-0.0010603	-0.2241566	0.22203596	0.99247361	Infected	
CAG.352	LDL Chol	-1.07E-05	-0.0001075	8.604880136914 87e-05	-0.0242307	-0.2429641	0.19450264	0.82599859	Infected	
CAG.352	Glucose	1.306489729843 91e-05	-4.52E-05	7.135668101387 35e-05	0.04910335	-0.1699816	0.26818825	0.65663424	Infected	
CAG.352	Systolic BP	-0.0008821	-0.00217	0.00040584	-0.115913	-0.285156	0.05333003	0.17660678	Infected	
Coprococcus	Insulin	0.0012393	-0.0008812	0.00335984	0.11896746	-0.0845959	0.32253085	0.24812356	Infected	
Coprococcus	Diastolic BP	0.0013925	-0.0022679	0.0050529	0.06434986	-0.1048039	0.23350364	0.45105197	Infected	
Coprococcus	Total Chol	-8.99E-05	-0.0004709	0.00029101	-0.0518616	-0.2715084	0.16778518	0.63956787	Infected	
Coprococcus	LDL Chol	-0.0001837	-0.0005184	0.00015109	-0.1174503	-0.3315258	0.09662526	0.27802821	Infected	
Coprococcus	Glucose	0.00018466	-1.44E-05	0.00038369	0.19635977	-0.0152864	0.40800598	0.06852528	Infected	
Coprococcus	Systolic BP	-3.01E-05	-0.0045721	0.00451202	-0.0011175	-0.1699857	0.16775073	0.98952051	Infected	
Enterococcus	Insulin	-0.0002583	-0.0008191	0.00030243	-0.0979656	-0.3106187	0.11468753	0.36183082	Infected	
Enterococcus	Diastolic BP	0.00015082	-0.000817	0.0011186	0.02753389	-0.1491386	0.20420637	0.75714751	Infected	
Enterococcus	Total Chol	-5.41E-05	-0.0001539	4.569376764577 76e-05	-0.1232425	-0.3505593	0.10407432	0.28369841	Infected	
Enterococcus	LDL Chol	-3.06E-05	-0.0001192	5.806416386436 11e-05	-0.0772455	-0.3011831	0.14669205	0.49422958	Infected	
Enterococcus	Glucose	-9.79E-06	-6.34E-05	4.377793671324 96e-05	-0.0411114	-0.2661214	0.18389848	0.71698874	Infected	
Enterococcus	Systolic BP	-0.0001432	-0.00134	0.00105352	-0.0210342	-0.1968001	0.15473177	0.81228438	Infected	
Akkermansia	Insulin	-0.0002527	-0.0007035	0.00019814	-0.1154732	-0.3214901	0.09054376	0.26784824	Infected	
Akkermansia	Diastolic BP	-7.99E-05	-0.0008603	0.00070048	-0.0175732	-0.1892371	0.15409069	0.83901287	Infected	
Akkermansia	Total Chol	-3.19E-05	-0.0001126	4.881932587786 09e-05	-0.0875823	-0.3091515	0.13398689	0.43363534	Infected	
Akkermansia	LDL Chol	-2.18E-05	-9.33E-05	4.967264307885 8e-05	-0.0664389	-0.2840945	0.15121664	0.54509121	Infected	
Akkermansia	Glucose	-4.22E-05	-8.43E-05	-2.02E-08	-0.2134147	-0.4267274	-0.0001021	0.04989319	Infected	*
Akkermansia	Systolic BP	-0.0001629	-0.0011272	0.00080136	-0.0288361	-0.199496	0.14182381	0.73744011	Infected	
Rikenellaceae_R C9_gut_group	Insulin	0.00032395	-0.0006087	0.00125665	0.07415548	-0.1393484	0.28765941	0.49125951	Infected	

Rikenellaceae_RC9_gut_group	Diastolic BP	0.00046269	-0.0011408	0.00206618	0.0509865	-0.1257108	0.22768378	0.56724897	Infected	
Rikenellaceae_RC9_gut_group	Total Chol	7.33467245690743e-05	-9.27E-05	0.00023938	0.10084109	-0.1274248	0.32910694	0.3817714	Infected	
Rikenellaceae_RC9_gut_group	LDL Chol	8.74999294640604e-05	-5.87E-05	0.0002337	0.133437	-0.0895107	0.35638466	0.23700352	Infected	
Rikenellaceae_RC9_gut_group	Glucose	-3.42E-05	-0.0001228	5.44174608417327e-05	-0.0867311	-0.3114471	0.13798481	0.4445158	Infected	
Rikenellaceae_RC9_gut_group	Systolic BP	0.00041256	-0.0015718	0.00239694	0.03657586	-0.1393504	0.21250208	0.6800317	Infected	
Lachnospiraceae_NK4A136_group	Insulin	-0.0001703	-0.0009364	0.00059577	-0.0454481	-0.2498838	0.15898759	0.65924123	Infected	
Lachnospiraceae_NK4A136_group	Diastolic BP	-0.0007001	-0.002008	0.00060772	-0.0899404	-0.2579507	0.0780699	0.2897675	Infected	
Lachnospiraceae_NK4A136_group	Total Chol	-1.55E-05	-0.0001523	0.00012125	-0.0248566	-0.2440496	0.1943364	0.82194814	Infected	
Lachnospiraceae_NK4A136_group	LDL Chol	6.30105125008276e-06	-0.0001147	0.00012725	0.01120208	-0.2038282	0.22623241	0.91764926	Infected	
Lachnospiraceae_NK4A136_group	Glucose	5.34956378002814e-05	-1.84E-05	0.00012541	0.15813523	-0.0544587	0.37072917	0.14264233	Infected	
Lachnospiraceae_NK4A136_group	Systolic BP	-0.000934	-0.0025488	0.00068093	-0.096527	-0.2634296	0.07037565	0.25303721	Infected	
Haemophilus	Insulin	-4.65E-05	-0.0009018	0.00080887	-0.0111976	-0.2172686	0.19487345	0.91411729	Infected	
Haemophilus	Diastolic BP	-0.0009043	-0.0023592	0.00055046	-0.1048837	-0.2736088	0.06384143	0.21954655	Infected	
Haemophilus	Total Chol	8.04049073096219e-05	-7.11E-05	0.00023187	0.1163453	-0.1028258	0.33551639	0.29379744	Infected	
Haemophilus	LDL Chol	9.31186115940958e-05	-4.01E-05	0.00022635	0.14945641	-0.0643774	0.36329021	0.1680019	Infected	
Haemophilus	Glucose	2.49550869941355e-05	-5.62E-05	0.0001061	0.06659806	-0.1499416	0.28313769	0.54206284	Infected	
Haemophilus	Systolic BP	-0.0016441	-0.0034217	0.00013353	-0.1534048	-0.3192689	0.01245919	0.06937306	Infected	
Ruminococcus_gauvreauii_group	Insulin	0.0008443	-0.0013482	0.00303683	0.07807423	-0.1246754	0.28082386	0.44555314	Infected	
Ruminococcus_gauvreauii_group	Diastolic BP	0.00293149	-0.0007896	0.00665262	0.13049729	-0.0351518	0.29614637	0.12081901	Infected	
Ruminococcus_gauvreauii_group	Total Chol	-7.94E-05	-0.0004715	0.00031269	-0.0441099	-0.2618885	0.17366878	0.68783227	Infected	
Ruminococcus_gauvreauii_group	LDL Chol	5.07131818922676e-05	-0.0002962	0.00039759	0.03124198	-0.182453	0.24493698	0.77174285	Infected	
Ruminococcus_gauvreauii_group	Glucose	3.58896909975777e-06	-0.0002057	0.00021286	0.00367631	-0.2106874	0.21803997	0.97284611	Infected	

Ruminococcus_gauvreauii_group	Systolic BP	0.0027814	-0.001849	0.00741184	0.0996133	-0.066222	0.26544862	0.2353283	Infected	
Lachnospiraceae_NK3A20_group	Insulin	-0.0005095	-0.0019377	0.00091861	-0.0727731	-0.2767432	0.13119706	0.47957403	Infected	
Lachnospiraceae_NK3A20_group	Diastolic BP	-0.0004407	-0.0028996	0.00201824	-0.0302974	-0.199355	0.13876027	0.72217326	Infected	
Lachnospiraceae_NK3A20_group	Total Chol	9.63392608190172e-05	-0.0001583	0.00035094	0.08263971	-0.1357599	0.30103937	0.45346777	Infected	
Lachnospiraceae_NK3A20_group	LDL Chol	0.00011587	-0.0001085	0.00034028	0.1102452	-0.1032785	0.32376893	0.3071161	Infected	
Lachnospiraceae_NK3A20_group	Glucose	-4.36E-05	-0.0001795	9.22530692555662e-05	-0.0690208	-0.283991	0.14594947	0.52450286	Infected	
Lachnospiraceae_NK3A20_group	Systolic BP	-0.0005743	-0.0036139	0.00246525	-0.031769	-0.1999006	0.13636254	0.70776268	Infected	
Turicibacter	Insulin	-0.0011612	-0.0030531	0.00073065	-0.1237013	-0.3252378	0.07783531	0.22535518	Infected	
Turicibacter	Diastolic BP	-0.0012614	-0.0045296	0.00200684	-0.0646856	-0.2322861	0.10291481	0.44452456	Infected	
Turicibacter	Total Chol	0.00038592	5.6703136739824e-05	0.00071514	0.24692017	0.03627977	0.45756058	0.02219332	Infected	*
Turicibacter	LDL Chol	0.00029108	-2.80E-06	0.00058497	0.20657968	-0.0019874	0.41514678	0.05216835	Infected	
Turicibacter	Glucose	5.06265407029406e-05	-0.0001306	0.00023189	0.05974092	-0.1541529	0.27363474	0.57971352	Infected	
Turicibacter	Systolic BP	-0.0029293	-0.0069303	0.00107173	-0.1208558	-0.285929	0.04421732	0.14894391	Infected	
Alloprevotella	Insulin	-0.0004128	-0.0018008	0.00097528	-0.0614792	-0.2682247	0.14526637	0.5554969	Infected	
Alloprevotella	Diastolic BP	0.00094353	-0.0014363	0.00332335	0.06765428	-0.1029855	0.23829409	0.43225534	Infected	
Alloprevotella	Total Chol	0.00024468	2.85409879083404e-06	0.0004865	0.21888914	0.00255328	0.43522499	0.04742152	Infected	*
Alloprevotella	LDL Chol	0.00012507	-9.25E-05	0.00034261	0.12410584	-0.0917576	0.33996927	0.25582687	Infected	
Alloprevotella	Glucose	7.32268660967958e-05	-5.80E-05	0.00020446	0.12081939	-0.0957073	0.33734606	0.26998403	Infected	
Alloprevotella	Systolic BP	0.00267074	-0.0002204	0.00556188	0.15406717	-0.012714	0.32084836	0.06970238	Infected	
Fusicatenibacter	Insulin	-0.0020061	-0.0047908	0.00077852	-0.1451443	-0.346615	0.05632636	0.15546514	Infected	
Fusicatenibacter	Diastolic BP	-0.0022094	-0.0070296	0.00261088	-0.0769503	-0.2448349	0.09093439	0.36425164	Infected	
Fusicatenibacter	Total Chol	6.9273218646244e-05	-0.0004337	0.00057226	0.03010221	-0.188467	0.24867141	0.78463199	Infected	
Fusicatenibacter	LDL Chol	-9.40E-05	-0.0005384	0.00035044	-0.0453076	-0.2595281	0.16891288	0.6748188	Infected	
Fusicatenibacter	Glucose	3.37477270362763e-05	-0.0002344	0.00030193	0.02704667	-0.1878872	0.24198053	0.80281105	Infected	
Fusicatenibacter	Systolic BP	-0.0075457	-0.0132871	-0.0018043	-0.2114358	-0.3723147	-0.050557	0.01067303	Infected	*
Butyricicoccus	Insulin	-0.0001578	-0.003211	0.00289535	-0.010571	-0.2150443	0.1939023	0.91827401	Infected	
Butyricicoccus	Diastolic BP	-0.0007276	-0.0059694	0.00451415	-0.0234572	-0.1924466	0.14553215	0.78297835	Infected	
Butyricicoccus	Total Chol	-0.0001943	-0.0007371	0.00034847	-0.0781564	-0.2964796	0.14016681	0.47810013	Infected	

Butyricicoccus	LDL Chol	-7.78E-05	-0.000559	0.00040341	-0.034698	-0.2493801	0.17998418	0.74844881	Infected	
Butyricicoccus	Glucose	0.0001635	-0.0001245	0.00045146	0.12129265	-0.0923233	0.33490856	0.26171249	Infected	
Butyricicoccus	Systolic BP	0.00058966	-0.0058924	0.00707171	0.01529405	-0.1528327	0.18342077	0.85673425	Infected	
UCG.010	Insulin	-0.0011365	-0.0035841	0.0013111	-0.0947183	-0.2987072	0.10927055	0.35806225	Infected	
UCG.010	Diastolic BP	0.00088217	-0.0033402	0.00510455	0.03539322	-0.1340112	0.20479765	0.67854714	Infected	
UCG.010	Total Chol	0.00047118	4.546366814986 34e-05	0.0008969	0.2358571	0.0227575	0.4489567	0.03051882	Infected	*
UCG.010	LDL Chol	0.00052988	0.00016087	0.00089889	0.29420368	0.08931737	0.49908999	0.0054605	Infected	**
UCG.010	Glucose	0.00016442	-6.66E-05	0.00039545	0.15179675	-0.0614857	0.36507919	0.16045528	Infected	
UCG.010	Systolic BP	-0.0015141	-0.0067286	0.0037005	-0.048871	-0.2171872	0.11944531	0.56484012	Infected	
Prevotella	Insulin	9.762807592962 33e-05	-0.0001955	0.0003908	0.09148093	-0.1832329	0.3661948	0.50245256	UnInfected	
Prevotella	Diastolic BP	0.00025953	-0.0001003	0.0006194	0.23717859	-0.0916932	0.56605037	0.15159228	UnInfected	
Prevotella	Total Chol	-1.37E-05	-6.23E-05	3.501022437859 14e-05	-0.089665	-0.4091232	0.22979334	0.5715044	UnInfected	
Prevotella	LDL Chol	-1.02E-05	-5.71E-05	3.660060405538 34e-05	-0.0725269	-0.4042644	0.25921053	0.65907975	UnInfected	
Prevotella	Glucose	1.763259249109 85e-05	-1.05E-05	4.572453546899 13e-05	0.20851799	-0.1236893	0.54072526	0.21025778	UnInfected	
Prevotella	Systolic BP	0.00053102	8.854874370881 86e-05	0.00097349	0.35491192	0.05918235	0.65064148	0.02019197	UnInfected	*
Blautia	Insulin	-0.0001644	-0.0005337	0.00020494	-0.1253633	-0.4070349	0.1563082	0.37140715	UnInfected	
Blautia	Diastolic BP	-0.0001491	-0.000617	0.00031881	-0.1108959	-0.4589343	0.23714248	0.52094985	UnInfected	
Blautia	Total Chol	-3.01E-06	-6.50E-05	5.894110275783 26e-05	-0.0161062	-0.3471027	0.31489041	0.9216642	UnInfected	
Blautia	LDL Chol	3.361871713712 35e-06	-5.61E-05	6.286672800124 38e-05	0.01937957	-0.3236373	0.3623964	0.90909962	UnInfected	
Blautia	Glucose	-2.32E-05	-5.87E-05	1.230288162268 25e-05	-0.2233936	-0.5652094	0.11842212	0.19251781	UnInfected	
Blautia	Systolic BP	0.00032594	-0.0002732	0.00092512	0.17731363	-0.148649	0.50327623	0.27610831	UnInfected	
Faecalibacterium	Insulin	-0.0004824	-0.0009969	3.203762106145 08e-05	-0.2542448	-0.5253736	0.01688404	0.06511955	UnInfected	
Faecalibacterium	Diastolic BP	6.525423127631 45e-05	-0.0006181	0.0007486	0.0335391	-0.3176829	0.38476106	0.84700457	UnInfected	
Faecalibacterium	Total Chol	-6.15E-05	-0.0001487	2.570383140551 51e-05	-0.2269929	-0.5488715	0.09488568	0.16057359	UnInfected	
Faecalibacterium	LDL Chol	-5.83E-05	-0.0001421	2.551788292805 2e-05	-0.2322195	-0.5660801	0.10164109	0.16620215	UnInfected	
Faecalibacterium	Glucose	-1.78E-05	-7.03E-05	3.480643013661 57e-05	-0.1181858	-0.4678695	0.23149793	0.49613627	UnInfected	
Faecalibacterium	Systolic BP	5.240646362794 02e-06	-0.0008812	0.00089167	0.00196995	-0.3312365	0.33517638	0.99046644	UnInfected	
Bifidobacterium	Insulin	-0.0001543	-0.00048	0.00017149	-0.1293163	-0.4023844	0.14375188	0.34196593	UnInfected	

Bifidobacterium	Diastolic BP	-6.05E-05	-0.000476	0.00035504	-0.049464	-0.3891803	0.2902524	0.76869985	UnInfected	
Bifidobacterium	Total Chol	7.401916049285 39e-06	-4.73E-05	6.208554087116 98e-05	0.04346176	-0.2776236	0.36454707	0.78453811	UnInfected	
Bifidobacterium	LDL Chol	1.193462372463 96e-05	-4.05E-05	6.434420812500 32e-05	0.07561252	-0.2564315	0.40765656	0.64589726	UnInfected	
Bifidobacterium	Glucose	-2.78E-05	-5.84E-05	2.854420654712 36e-06	-0.2939496	-0.6180964	0.03019715	0.07398604	UnInfected	
Bifidobacterium	Systolic BP	-0.0002796	-0.0008096	0.00025036	-0.1671823	-0.4840585	0.14969385	0.29055657	UnInfected	
Clostridium_sen su_stricto_1	Insulin	1.948501602238 44e-05	-0.0002409	0.00027984	0.02084334	-0.2576561	0.29934282	0.87979131	UnInfected	
Clostridium_sen su_stricto_1	Diastolic BP	-2.56E-05	-0.0003534	0.00030223	-0.0266924	-0.3686938	0.31530907	0.8746857	UnInfected	
Clostridium_sen su_stricto_1	Total Chol	4.044790892854 3e-06	-3.91E-05	4.716905195611 77e-05	0.03030745	-0.2928208	0.35343571	0.849692	UnInfected	
Clostridium_sen su_stricto_1	LDL Chol	1.017043636554 77e-05	-3.11E-05	5.145239499825 07e-05	0.08222705	-0.2515338	0.41598791	0.619223	UnInfected	
Clostridium_sen su_stricto_1	Glucose	2.652243299244 5e-07	-2.51E-05	2.566839335009 17e-05	0.00358056	-0.3393656	0.34652676	0.98316488	UnInfected	
Clostridium_sen su_stricto_1	Systolic BP	9.675506237766 61e-06	-0.0004155	0.00043482	0.00738234	-0.3170027	0.33176743	0.96331424	UnInfected	
Subdoligranulum	Insulin	-0.0007059	-0.0014972	8.535913166622 04e-05	-0.2467429	-0.5233218	0.02983605	0.07856231	UnInfected	
Subdoligranulum	Diastolic BP	0.00047308	-0.0005595	0.00150564	0.16126969	-0.190721	0.51326036	0.35767926	UnInfected	
Subdoligranulum	Total Chol	-4.88E-05	-0.0001854	8.772344050409 62e-05	-0.1195797	-0.4539394	0.21477989	0.47161462	UnInfected	
Subdoligranulum	LDL Chol	-1.51E-05	-0.0001473	0.00011702	-0.0399468	-0.3890326	0.30913906	0.81717495	UnInfected	
Subdoligranulum	Glucose	-3.30E-05	-0.0001132	4.722573698382 21e-05	-0.1454161	-0.4991572	0.20832505	0.40860988	UnInfected	
Subdoligranulum	Systolic BP	0.00068096	-0.0006534	0.00201535	0.16977249	-0.1629079	0.50245293	0.30637153	UnInfected	
Romboutsia	Insulin	-3.61E-06	-0.0004552	0.00044798	-0.002215	-0.2791541	0.27472404	0.98710268	UnInfected	
Romboutsia	Diastolic BP	-0.0004551	-0.0009996	8.940266190217 66e-05	-0.2721977	-0.5978657	0.05347018	0.0983589	UnInfected	
Romboutsia	Total Chol	-1.75E-05	-9.20E-05	5.708096149764 37e-05	-0.0750519	-0.3952993	0.24519551	0.63634488	UnInfected	
Romboutsia	LDL Chol	1.606856231519 95e-05	-5.56E-05	8.769712771979 85e-05	0.07447664	-0.2575167	0.40646994	0.65079477	UnInfected	
Romboutsia	Glucose	6.503317330150 51e-06	-3.75E-05	5.048910967675 41e-05	0.05033163	-0.290091	0.39075429	0.7652397	UnInfected	
Romboutsia	Systolic BP	-0.0004404	-0.0011604	0.00027955	-0.1926354	-0.5075493	0.12227839	0.22180645	UnInfected	
Collinsella	Insulin	0.00028142	-0.0001993	0.00076217	0.16543852	-0.1171799	0.44805693	0.2418814	UnInfected	
Collinsella	Diastolic BP	-0.0004047	-0.0010059	0.00019657	-0.2320152	-0.5767328	0.11270242	0.17992193	UnInfected	
Collinsella	Total Chol	-8.70E-06	-9.00E-05	7.263786611132 95e-05	-0.0358246	-0.3707582	0.29910903	0.82891311	UnInfected	
Collinsella	LDL Chol	1.057076058157 73e-05	-6.75E-05	8.866016745239 77e-05	0.04696718	-0.2999936	0.39392797	0.78452494	UnInfected	
Collinsella	Glucose	2.533906653060 75e-05	-2.17E-05	7.238455683791 8e-05	0.18799308	-0.1610421	0.53702829	0.28078164	UnInfected	

Collinsella	Systolic BP	-0.0001229	-0.0009237	0.00067794	-0.0515285	-0.3873215	0.28426441	0.75663221	UnInfected	
Agathobacter	Insulin	-8.75E-05	-0.0008562	0.00068115	-0.0312273	-0.3055165	0.24306197	0.81809186	UnInfected	
Agathobacter	Diastolic BP	0.00016705	-0.0007998	0.00113388	0.05813764	-0.2783365	0.39461182	0.72718208	UnInfected	
Agathobacter	Total Chol	-3.32E-05	-0.0001601	9.364723878693 79e-05	-0.0830913	-0.4002598	0.23407719	0.59728335	UnInfected	
Agathobacter	LDL Chol	-6.06E-05	-0.0001811	5.983701662831 1e-05	-0.1634903	-0.4883629	0.16138229	0.31301463	UnInfected	
Agathobacter	Glucose	2.777955636452 6e-05	-4.66E-05	0.00010215	0.12510487	-0.2098041	0.46001383	0.45229106	UnInfected	
Agathobacter	Systolic BP	-0.0006684	-0.001901	0.00056417	-0.1701235	-0.4838428	0.14359578	0.27757521	UnInfected	
Catenibacterium	Insulin	-2.66E-05	-0.0007635	0.00071041	-0.0099557	-0.2861555	0.26624418	0.94192773	UnInfected	
Catenibacterium	Diastolic BP	0.00044128	-0.0004731	0.00135564	0.16129056	-0.1729174	0.49549854	0.33296477	UnInfected	
Catenibacterium	Total Chol	-4.70E-05	-0.0001679	7.394734193875 43e-05	-0.1233368	-0.4407988	0.19412525	0.43455701	UnInfected	
Catenibacterium	LDL Chol	-5.62E-05	-0.0001717	5.933918207210 54e-05	-0.1591495	-0.4863816	0.16808263	0.32928214	UnInfected	
Catenibacterium	Glucose	1.858373473899 97e-05	-5.30E-05	9.016066636063 68e-05	0.08789765	-0.2506481	0.42644338	0.60055535	UnInfected	
Catenibacterium	Systolic BP	0.0002526	-0.0009471	0.00145232	0.06752359	-0.2531837	0.38823092	0.67089287	UnInfected	
UCG.002	Insulin	7.261367687971 49e-05	-0.0009332	0.00107839	0.02024993	-0.2602332	0.30073302	0.88400754	UnInfected	
UCG.002	Diastolic BP	-2.17E-05	-0.0012886	0.00124512	-0.0059132	-0.3504713	0.33864483	0.97233087	UnInfected	
UCG.002	Total Chol	-6.22E-05	-0.0002274	0.00010297	-0.1215084	-0.4441625	0.20114564	0.44865119	UnInfected	
UCG.002	LDL Chol	-2.73E-05	-0.0001871	0.00013253	-0.0574838	-0.394298	0.27933043	0.73038568	UnInfected	
UCG.002	Glucose	1.915350683967 65e-05	-7.87E-05	0.00011705	0.06741009	-0.2771185	0.41193865	0.69287604	UnInfected	
UCG.002	Systolic BP	-0.0014237	-0.0029841	0.00013666	-0.2831925	-0.5935687	0.02718357	0.07231264	UnInfected	
Streptococcus	Insulin	2.621023986743 74e-05	-0.0003756	0.00042806	0.01813738	-0.2599408	0.29621555	0.89513841	UnInfected	
Streptococcus	Diastolic BP	0.00019254	-0.0003088	0.00069391	0.12994368	-0.2084256	0.46831292	0.43982269	UnInfected	
Streptococcus	Total Chol	6.670985118650 04e-06	-5.99E-05	7.322191608657 46e-05	0.03233555	-0.2902496	0.35492072	0.83950608	UnInfected	
Streptococcus	LDL Chol	1.218660100987 65e-05	-5.16E-05	7.599909202128 39e-05	0.06373745	-0.2700099	0.39748477	0.69984998	UnInfected	
Streptococcus	Glucose	-8.85E-06	-4.79E-05	3.022972581728 54e-05	-0.0772645	-0.4185322	0.26400325	0.64779564	UnInfected	
Streptococcus	Systolic BP	-6.99E-05	-0.0007256	0.00058583	-0.0344826	-0.3581215	0.2891563	0.82956429	UnInfected	
Holdemanella	Insulin	1.816815992102 6e-05	-0.0013051	0.00134148	0.00386022	-0.2773072	0.28502759	0.97786328	UnInfected	
Holdemanella	Diastolic BP	0.00021399	-0.0014505	0.00187854	0.04434328	-0.3005802	0.38926672	0.79510175	UnInfected	
Holdemanella	Total Chol	-4.69E-05	-0.0002655	0.00017168	-0.0698147	-0.39514	0.2555107	0.66495663	UnInfected	

Holdemanella	LDL Chol	-2.58E-05	-0.0002362	0.00018458	-0.0414303	-0.3792639	0.29640323	0.80433871	UnInfected	
Holdemanella	Glucose	8.452299245536 55e-05	-4.09E-05	0.00020996	0.22664574	-0.1097075	0.56299893	0.17943582	UnInfected	
Holdemanella	Systolic BP	0.00073598	-0.001408	0.00287994	0.1115382	-0.2133776	0.43645401	0.48945052	UnInfected	
Dorea	Insulin	0.00014065	-0.0018374	0.00211872	0.01961483	-0.2562367	0.29546639	0.8857463	UnInfected	
Dorea	Diastolic BP	0.00085021	-0.0016225	0.0033229	0.11563421	-0.2206695	0.45193794	0.4887536	UnInfected	
Dorea	Total Chol	-0.0001482	-0.0004717	0.0001752	-0.1448092	-0.4607626	0.17114415	0.35751359	UnInfected	
Dorea	LDL Chol	-0.0002255	-0.0005297	7.876086083489 9e-05	-0.2376384	-0.5582913	0.08301454	0.14096059	UnInfected	
Dorea	Glucose	8.938465885245 35e-05	-0.0001009	0.00027968	0.15731483	-0.1776045	0.49223413	0.34585769	UnInfected	
Dorea	Systolic BP	-0.001147	-0.0043506	0.00205662	-0.1140922	-0.4327553	0.20457079	0.47112793	UnInfected	
Bacteroides	Insulin	0.00019762	-0.000148	0.0005432	0.15768944	-0.1180643	0.43344319	0.25270304	UnInfected	
Bacteroides	Diastolic BP	-0.0001985	-0.000637	0.00024001	-0.1544634	-0.4957054	0.18677854	0.36341819	UnInfected	
Bacteroides	Total Chol	-5.99E-07	-5.91E-05	5.785563301092 21e-05	-0.0033492	-0.3300695	0.32337111	0.98347059	UnInfected	
Bacteroides	LDL Chol	-4.82E-06	-6.09E-05	5.130076293233 81e-05	-0.0290576	-0.3675015	0.30938641	0.86227289	UnInfected	
Bacteroides	Glucose	3.933047371861 37e-06	-3.05E-05	3.831869758247 03e-05	0.03960678	-0.3066654	0.38587898	0.81725659	UnInfected	
Bacteroides	Systolic BP	-0.0003791	-0.0009387	0.00018049	-0.2157529	-0.5342317	0.10272585	0.17717809	UnInfected	
Escherichia.Shig ella	Insulin	-7.31E-05	-0.0003779	0.00023176	-0.0700281	-0.3621849	0.22212875	0.6287091	UnInfected	
Escherichia.Shig ella	Diastolic BP	-2.68E-06	-0.0003879	0.00038258	-0.0025042	-0.3626212	0.35761281	0.98878671	UnInfected	
Escherichia.Shig ella	Total Chol	2.060928775994 88e-05	-2.95E-05	7.075270696592 23e-05	0.13836057	-0.1982776	0.47499871	0.40869296	UnInfected	
Escherichia.Shig ella	LDL Chol	1.143145916122 16e-05	-3.71E-05	5.994403283933 27e-05	0.08280811	-0.2686111	0.43422734	0.63450553	UnInfected	
Escherichia.Shig ella	Glucose	-1.02E-05	-3.99E-05	1.937200799359 22e-05	-0.1238862	-0.4820922	0.23431971	0.48623127	UnInfected	
Escherichia.Shig ella	Systolic BP	8.698447448826 83e-06	-0.0004908	0.00050815	0.00594646	-0.3354921	0.34738501	0.97192134	UnInfected	
Christensenellac eae_R.7_group	Insulin	0.00025792	-0.001673	0.00218886	0.0403416	-0.2616752	0.3423584	0.78730733	UnInfected	
Christensenellac eae_R.7_group	Diastolic BP	-0.0003742	-0.0028047	0.00205632	-0.057077	-0.4278294	0.31367532	0.7558718	UnInfected	
Christensenellac eae_R.7_group	Total Chol	-9.24E-05	-0.0004109	0.00022614	-0.1012345	-0.4502278	0.2477588	0.55876151	UnInfected	
Christensenellac eae_R.7_group	LDL Chol	-3.74E-05	-0.0003448	0.00026988	-0.044269	-0.4075736	0.31903551	0.80556375	UnInfected	
Christensenellac eae_R.7_group	Glucose	4.554457799615 e-05	-0.0001423	0.00023339	0.08990257	-0.28089	0.46069512	0.62476686	UnInfected	
Christensenellac eae_R.7_group	Systolic BP	-0.0027546	-0.0057505	0.00024122	-0.3073141	-0.6415393	0.02691104	0.07023917	UnInfected	
Eubacterium_hal lii_group	Insulin	-0.0004048	-0.0018038	0.00099425	-0.0814391	-0.3629113	0.200033	0.55976353	UnInfected	

Eubacterium_hallii_group	Diastolic BP	-2.14E-05	-0.0017926	0.00174977	-0.0041982	-0.3517287	0.34333224	0.98052158	UnInfected	
Eubacterium_hallii_group	Total Chol	-7.09E-05	-0.0003025	0.00016076	-0.0998771	-0.4263149	0.22656062	0.53755591	UnInfected	
Eubacterium_hallii_group	LDL Chol	-2.71E-05	-0.0002507	0.00019649	-0.041257	-0.3812901	0.29877605	0.80637396	UnInfected	
Eubacterium_hallii_group	Glucose	-5.59E-05	-0.0001917	7.97631836530651e-05	-0.1420565	-0.4866392	0.20252632	0.40728511	UnInfected	
Eubacterium_hallii_group	Systolic BP	0.00137941	-0.0008624	0.00362126	0.1979501	-0.1237617	0.51966192	0.21916604	UnInfected	
Muribaculaceae	Insulin	-0.0001271	-0.0012152	0.00096109	-0.0333751	-0.3192077	0.25245751	0.81352037	UnInfected	
Muribaculaceae	Diastolic BP	-0.0008654	-0.0022009	0.00047016	-0.2216908	-0.5638296	0.12044795	0.19625208	UnInfected	
Muribaculaceae	Total Chol	-0.0001563	-0.0003277	1.51284881832536e-05	-0.2875977	-0.6030313	0.02783599	0.0725102	UnInfected	
Muribaculaceae	LDL Chol	-8.93E-05	-0.0002596	8.09918142891593e-05	-0.1773026	-0.5154008	0.16079556	0.29342643	UnInfected	
Muribaculaceae	Glucose	-3.66E-05	-0.0001421	6.87612623262979e-05	-0.1214843	-0.4709188	0.22795008	0.48397263	UnInfected	
Muribaculaceae	Systolic BP	-0.0015637	-0.0032501	0.00012271	-0.2929735	-0.6089382	0.0229912	0.06802288	UnInfected	
Dialister	Insulin	-0.0002696	-0.0011564	0.00061727	-0.0868109	-0.3724138	0.19879201	0.54020774	UnInfected	
Dialister	Diastolic BP	-0.0003768	-0.001492	0.00073832	-0.118353	-0.4686	0.23189396	0.49621984	UnInfected	
Dialister	Total Chol	7.62864778859187e-05	-6.89E-05	0.00022151	0.17208974	-0.1555192	0.49969869	0.29263254	UnInfected	
Dialister	LDL Chol	8.09583954729586e-05	-5.80E-05	0.0002199	0.19705667	-0.141124	0.53523733	0.24399852	UnInfected	
Dialister	Glucose	-4.33E-05	-0.0001289	4.22709857771162e-05	-0.1761302	-0.524065	0.17180452	0.31021124	UnInfected	
Dialister	Systolic BP	7.34426616404046e-05	-0.0013827	0.00152956	0.01687031	-0.3176097	0.35135035	0.91881231	UnInfected	
uncultured	Insulin	0.00169528	-0.0005745	0.00396511	0.19857876	-0.0673001	0.46445757	0.13799599	UnInfected	
uncultured	Diastolic BP	0.00107618	-0.001858	0.00401039	0.12294241	-0.2122622	0.458147	0.46046826	UnInfected	
uncultured	Total Chol	6.53792249829253e-06	-0.0003829	0.00039594	0.00536436	-0.3141423	0.32487102	0.97293083	UnInfected	
uncultured	LDL Chol	-9.19E-06	-0.0003832	0.00036482	-0.008134	-0.3392544	0.32298648	0.96040378	UnInfected	
uncultured	Glucose	7.66899741017703e-05	-0.0001509	0.00030428	0.11337083	-0.2230795	0.44982111	0.4974245	UnInfected	
uncultured	Systolic BP	0.00016169	-0.0036749	0.00399828	0.01350933	-0.307037	0.33405567	0.93212376	UnInfected	
Succinivibrio	Insulin	-0.0003652	-0.0012974	0.00056706	-0.1089028	-0.3869233	0.16911773	0.43082005	UnInfected	
Succinivibrio	Diastolic BP	-0.0005254	-0.0016957	0.00064483	-0.1528272	-0.4932111	0.1875566	0.36726679	UnInfected	
Succinivibrio	Total Chol	6.77517914592206e-05	-8.63E-05	0.0002218	0.141536	-0.1802864	0.46335843	0.37703127	UnInfected	
Succinivibrio	LDL Chol	6.92739406144789e-05	-7.84E-05	0.00021699	0.15614852	-0.1768212	0.48911826	0.34662025	UnInfected	
Succinivibrio	Glucose	-3.25E-05	-0.0001236	5.85620616570003e-05	-0.122383	-0.4651842	0.22041812	0.4723886	UnInfected	

Succinivibrio	Systolic BP	0.00022493	-0.0013098	0.00175965	0.04784711	-0.2786234	0.37431762	0.76722836	UnInfected	
UCG.005	Insulin	-0.000435	-0.0031102	0.00224024	-0.0442737	-0.3165687	0.22802118	0.74265653	UnInfected	
UCG.005	Diastolic BP	-5.98E-05	-0.0034341	0.00331454	-0.0059321	-0.3408907	0.32902647	0.97144746	UnInfected	
UCG.005	Total Chol	-8.96E-05	-0.0005324	0.00035315	-0.0639146	-0.3796128	0.25178364	0.68280591	UnInfected	
UCG.005	LDL Chol	2.864630244058 92e-05	-0.0003977	0.00045495	0.02203729	-0.3059153	0.34998991	0.8919872	UnInfected	
UCG.005	Glucose	-2.20E-05	-0.0002833	0.00023922	-0.0283162	-0.3639219	0.30728949	0.86462756	UnInfected	
UCG.005	Systolic BP	-0.0026524	-0.0069215	0.00161665	-0.1925662	-0.5025012	0.11736874	0.21480429	UnInfected	
Clostridia_UCG. 014	Insulin	-0.0013001	-0.0031947	0.00059457	-0.1921267	-0.4721194	0.0878659	0.17181439	UnInfected	
Clostridia_UCG. 014	Diastolic BP	-0.0006426	-0.0030891	0.00180396	-0.0926123	-0.4452253	0.26000064	0.5963538	UnInfected	
Clostridia_UCG. 014	Total Chol	-4.11E-05	-0.0003641	0.00028194	-0.0425007	-0.3768501	0.29184865	0.79735199	UnInfected	
Clostridia_UCG. 014	LDL Chol	-3.00E-05	-0.0003404	0.00028038	-0.0334941	-0.3801571	0.31316885	0.84522418	UnInfected	
Clostridia_UCG. 014	Glucose	-0.0001825	-0.0003611	-3.80E-06	-0.340297	-0.6735078	-0.0070862	0.04559328	UnInfected	*
Clostridia_UCG. 014	Systolic BP	-0.0008471	-0.0040184	0.00232413	-0.0892964	-0.4235746	0.24498183	0.5901237	UnInfected	
Intestinibacter	Insulin	-0.0001256	-0.0013314	0.0010802	-0.0285884	-0.3030286	0.24585188	0.8333071	UnInfected	
Intestinibacter	Diastolic BP	-0.0006423	-0.002144	0.00085944	-0.1425645	-0.4758991	0.1907701	0.39014514	UnInfected	
Intestinibacter	Total Chol	2.674823117015 98e-05	-0.0001729	0.00022643	0.04264328	-0.275695	0.36098161	0.78671468	UnInfected	
Intestinibacter	LDL Chol	8.113883965649 04e-05	-0.0001086	0.00027092	0.13957453	-0.1868795	0.46602859	0.39030359	UnInfected	
Intestinibacter	Glucose	-8.65E-06	-0.0001263	0.00010901	-0.0248414	-0.362788	0.31310518	0.88191845	UnInfected	
Intestinibacter	Systolic BP	-0.0002876	-0.0022547	0.0016795	-0.0466854	-0.3660197	0.27264896	0.76778735	UnInfected	
Ruminococcus	Insulin	-0.0008055	-0.0019777	0.00036666	-0.1847873	-0.4536883	0.08411365	0.17120275	UnInfected	
Ruminococcus	Diastolic BP	0.00074379	-0.0007529	0.00224053	0.16640919	-0.1684563	0.50127473	0.31901611	UnInfected	
Ruminococcus	Total Chol	5.120891194324 79e-05	-0.000148	0.00025041	0.08228676	-0.2378127	0.40238622	0.60414595	UnInfected	
Ruminococcus	LDL Chol	1.897181158806 2e-05	-0.0001731	0.000211	0.03289388	-0.3000554	0.36584316	0.84178483	UnInfected	
Ruminococcus	Glucose	-5.08E-05	-0.0001671	6.557636153339 6e-05	-0.1470057	-0.483864	0.1898526	0.3806787	UnInfected	
Ruminococcus	Systolic BP	0.00026893	-0.0016999	0.00223779	0.04400426	-0.2781515	0.36616001	0.78262491	UnInfected	
Sarcina	Insulin	0.00042919	-5.47E-05	0.00091309	0.24814218	-0.0316304	0.5279147	0.08023018	UnInfected	
Sarcina	Diastolic BP	-0.0001139	-0.0007522	0.00052447	-0.0641981	-0.424129	0.29573269	0.71876065	UnInfected	
Sarcina	Total Chol	-3.60E-05	-0.0001192	4.715181539355 62e-05	-0.1458039	-0.4825646	0.19095674	0.38440176	UnInfected	

Sarcina	LDL Chol	-3.03E-05	-0.0001104	4.981281319083 34e-05	-0.1323259	-0.4823222	0.21767045	0.44687747	UnInfected	
Sarcina	Glucose	7.171019687472 01e-06	-4.23E-05	5.665113385070 41e-05	0.05232416	-0.3087131	0.41336146	0.76974252	UnInfected	
Sarcina	Systolic BP	-2.30E-05	-0.0008522	0.00080623	-0.0094748	-0.3514286	0.33247895	0.95534281	UnInfected	
Eubacterium_co prostanoligenes_ group	Insulin	0.00068953	-0.0033065	0.00468556	0.04916498	-0.235759	0.33408896	0.72753031	UnInfected	
Eubacterium_co prostanoligenes_ group	Diastolic BP	-0.0003505	-0.0053904	0.00468938	-0.024373	-0.3748398	0.3260938	0.88823871	UnInfected	
Eubacterium_co prostanoligenes_ group	Total Chol	-5.52E-05	-0.0007182	0.00060779	-0.0275803	-0.3587198	0.30355919	0.86635008	UnInfected	
Eubacterium_co prostanoligenes_ group	LDL Chol	-1.96E-05	-0.0006566	0.00061751	-0.010542	-0.3538586	0.33277454	0.95051633	UnInfected	
Eubacterium_co prostanoligenes_ group	Glucose	-4.53E-05	-0.0004355	0.00034486	-0.0407771	-0.3918791	0.31032488	0.81449806	UnInfected	
Eubacterium_co prostanoligenes_ group	Systolic BP	-0.0016874	-0.008195	0.00482019	-0.0858163	-0.4167754	0.24514286	0.6010267	UnInfected	
Lactobacillus	Insulin	0.00103186	-0.0005172	0.00258095	0.17912961	-0.0897887	0.44804792	0.18433179	UnInfected	
Lactobacillus	Diastolic BP	4.756190773066 06e-05	-0.0019583	0.0020534	0.0080525	-0.331548	0.34765299	0.96177803	UnInfected	
Lactobacillus	Total Chol	-1.50E-05	-0.0002788	0.00024891	-0.0181867	-0.3390476	0.30267429	0.90880597	UnInfected	
Lactobacillus	LDL Chol	-0.0001169	-0.0003669	0.00013306	-0.1534047	-0.4813857	0.17457636	0.34787187	UnInfected	
Lactobacillus	Glucose	-9.84E-06	-0.0001652	0.00014551	-0.0215473	-0.3618779	0.3187833	0.89819322	UnInfected	
Lactobacillus	Systolic BP	-0.001455	-0.0040022	0.00109222	-0.1801594	-0.4955602	0.13524144	0.25322443	UnInfected	
Methanobreviba cter	Insulin	1.475556713555 25e-05	-0.0023313	0.00236081	0.00177634	-0.2806516	0.28420423	0.98985783	UnInfected	
Methanobreviba cter	Diastolic BP	-0.0009225	-0.0038579	0.00201286	-0.1083125	-0.4529502	0.23632517	0.52662215	UnInfected	
Methanobreviba cter	Total Chol	-0.0002989	-0.0006724	7.456192583682 2e-05	-0.2520625	-0.5669995	0.06287444	0.11284832	UnInfected	
Methanobreviba cter	LDL Chol	-0.0003188	-0.000674	3.646372172657 78e-05	-0.2900438	-0.6132649	0.03317721	0.07690913	UnInfected	
Methanobreviba cter	Glucose	-1.52E-05	-0.000244	0.00021356	-0.0231006	-0.3706619	0.32446065	0.89315588	UnInfected	
Methanobreviba cter	Systolic BP	0.00079363	-0.0030255	0.00461277	0.06814664	-0.2597898	0.3960831	0.67491966	UnInfected	
Roseburia	Insulin	-0.0006156	-0.0023599	0.00112874	-0.105969	-0.4062433	0.19430525	0.47745068	UnInfected	
Roseburia	Diastolic BP	-0.0017702	-0.0038906	0.00035015	-0.2971947	-0.6531756	0.05878623	0.0987268	UnInfected	
Roseburia	Total Chol	6.328094535357 48e-05	-0.0002271	0.0003537	0.0763046	-0.273886	0.42649522	0.66014761	UnInfected	
Roseburia	LDL Chol	8.365713591070 56e-05	-0.0001945	0.00036183	0.10884352	-0.2530833	0.47077031	0.54448696	UnInfected	

Roseburia	Glucose	-7.96E-05	-0.0002487	8.951866847640 37e-05	-0.172883	-0.5402466	0.19448055	0.34495219	UnInfected	
Roseburia	Systolic BP	-0.0002686	-0.0031374	0.00260015	-0.0329852	-0.385229	0.31925861	0.84993059	UnInfected	
Ruminococcus_t orques_group	Insulin	-5.40E-05	-0.0034354	0.0033274	-0.004376	-0.2782989	0.26954688	0.97424287	UnInfected	
Ruminococcus_t orques_group	Diastolic BP	0.00320998	-0.000888	0.007308	0.25360516	-0.0701605	0.57737084	0.12042468	UnInfected	
Ruminococcus_t orques_group	Total Chol	-0.0001126	-0.0006713	0.00044616	-0.0638893	-0.3809452	0.25316649	0.68420488	UnInfected	
Ruminococcus_t orques_group	LDL Chol	-7.56E-05	-0.000613	0.0004618	-0.0462846	-0.375314	0.28274481	0.77631501	UnInfected	
Ruminococcus_t orques_group	Glucose	5.416289802728 83e-05	-0.0002751	0.00038341	0.05537362	-0.2812325	0.3919797	0.73974612	UnInfected	
Ruminococcus_t orques_group	Systolic BP	0.00085495	-0.0046565	0.00636644	0.04939994	-0.2690583	0.36785821	0.75407438	UnInfected	
Treponema	Insulin	0.00038185	-0.0006602	0.00142388	0.10689985	-0.1848151	0.39861481	0.46085047	UnInfected	
Treponema	Diastolic BP	-0.0003533	-0.0016707	0.00096403	-0.0964668	-0.4561403	0.26320669	0.5886359	UnInfected	
Treponema	Total Chol	0.00016895	5.786209086837 39e-06	0.00033211	0.33129645	0.01134645	0.65124645	0.04284561	UnInfected	*
Treponema	LDL Chol	0.00018705	3.395845071786 65e-05	0.00034014	0.39577346	0.07185143	0.71969549	0.01821208	UnInfected	*
Treponema	Glucose	4.779856595294 45e-05	-5.33E-05	0.00014886	0.168875	-0.1881888	0.52593876	0.34258229	UnInfected	
Treponema	Systolic BP	-9.22E-05	-0.0018076	0.00162332	-0.0184038	-0.3609501	0.32414245	0.91353905	UnInfected	
Terrisporobacter	Insulin	0.0008142	-0.0014751	0.00310346	0.09945794	-0.1801862	0.37910206	0.47405122	UnInfected	
Terrisporobacter	Diastolic BP	-0.0026713	-0.0054136	7.106101022181 72e-05	-0.3182427	-0.6449513	0.0084658	0.05587137	UnInfected	
Terrisporobacter	Total Chol	-0.0001298	-0.0005093	0.00024965	-0.111103	-0.4358218	0.21361574	0.49087282	UnInfected	
Terrisporobacter	LDL Chol	7.401924479752 27e-05	-0.0002923	0.00044031	0.06833817	-0.2698412	0.40651756	0.68336443	UnInfected	
Terrisporobacter	Glucose	0.00012534	-9.52E-05	0.00034588	0.1932289	-0.1467693	0.53322713	0.25558025	UnInfected	
Terrisporobacter	Systolic BP	-0.0023504	-0.0060217	0.00132092	-0.2047897	-0.5246704	0.11509106	0.20151681	UnInfected	
Senegalimassilia	Insulin	-0.0014752	-0.0040153	0.00106493	-0.1562596	-0.4253225	0.11280329	0.24554004	UnInfected	
Senegalimassilia	Diastolic BP	-0.0007477	-0.0040043	0.0025088	-0.0772471	-0.4136704	0.25917622	0.64316464	UnInfected	
Senegalimassilia	Total Chol	0.00020333	-0.0002203	0.00062698	0.15086227	-0.1634768	0.4652013	0.33560764	UnInfected	
Senegalimassilia	LDL Chol	0.00031212	-8.52E-05	0.00070948	0.24987987	-0.068241	0.56800069	0.11942987	UnInfected	
Senegalimassilia	Glucose	-0.0001503	-0.0003976	9.692862990654 34e-05	-0.2009678	-0.5315117	0.12957618	0.22456244	UnInfected	
Senegalimassilia	Systolic BP	0.00107322	-0.0031454	0.00529185	0.08108595	-0.237647	0.39981886	0.60788503	UnInfected	
Weissella	Insulin	-8.11E-05	-0.0004805	0.00031834	-0.0560386	-0.3321039	0.22002672	0.68201458	UnInfected	
Weissella	Diastolic BP	1.412680183954 28e-05	-0.0004901	0.00051838	0.0095227	-0.3303874	0.34943281	0.95484795	UnInfected	

Weissella	Total Chol	-4.79E-06	-7.11E-05	6.15386442825725e-05	-0.0231797	-0.3442962	0.29793685	0.8840266	UnInfected	
Weissella	LDL Chol	-1.50E-05	-7.85E-05	4.8474055421214e-05	-0.078481	-0.4101876	0.25322553	0.63313281	UnInfected	
Weissella	Glucose	-5.45E-06	-4.45E-05	3.35587268463059e-05	-0.0475739	-0.3878773	0.29272956	0.77766282	UnInfected	
Weissella	Systolic BP	0.00065593	4.63269207706187e-05	0.00126554	0.32337164	0.022839	0.62390428	0.03579129	UnInfected	*
CAG.352	Insulin	-0.0006869	-0.0023391	0.00096533	-0.1132259	-0.3855771	0.15912529	0.40338191	UnInfected	
CAG.352	Diastolic BP	0.00219371	0.00024402	0.00414341	0.35267008	0.03922939	0.66611076	0.0286391	UnInfected	*
CAG.352	Total Chol	4.18549714515738e-05	-0.0002345	0.00031822	0.04832763	-0.2707802	0.36743544	0.75971219	UnInfected	
CAG.352	LDL Chol	1.83161976610018e-05	-0.0002474	0.00028408	0.02281947	-0.3082839	0.35392289	0.88923625	UnInfected	
CAG.352	Glucose	6.12306034180087e-05	-0.0001002	0.00022268	0.12738017	-0.2084939	0.46325428	0.44548299	UnInfected	
CAG.352	Systolic BP	0.00035349	-0.0023708	0.00307773	0.04156111	-0.2787424	0.36186461	0.79324121	UnInfected	
Coprococcus	Insulin	0.00272269	-0.0008617	0.00630709	0.20095691	-0.0636008	0.46551458	0.13163787	UnInfected	
Coprococcus	Diastolic BP	-0.0025066	-0.0070979	0.00208473	-0.1804335	-0.5109326	0.15006549	0.27440219	UnInfected	
Coprococcus	Total Chol	-0.0001736	-0.000786	0.00043892	-0.0897257	-0.4063746	0.22692319	0.56785274	UnInfected	
Coprococcus	LDL Chol	-0.0001106	-0.0007006	0.00047939	-0.0616958	-0.3908163	0.26742479	0.70510684	UnInfected	
Coprococcus	Glucose	9.30919380266846e-06	-0.0003531	0.00037175	0.00867137	-0.3289384	0.34628111	0.9586007	UnInfected	
Coprococcus	Systolic BP	-0.0013965	-0.0074418	0.00464894	-0.0735163	-0.3917769	0.24474421	0.64117513	UnInfected	
Enterococcus	Insulin	3.87716701571712e-06	-0.0008394	0.00084716	0.00127352	-0.2757148	0.27826186	0.99258586	UnInfected	
Enterococcus	Diastolic BP	0.00127557	0.0003182	0.00223294	0.40862189	0.10193422	0.71530957	0.01061903	UnInfected	*
Enterococcus	Total Chol	9.28583554656159e-05	-4.28E-05	0.00022851	0.21364736	-0.098453	0.52574767	0.17281013	UnInfected	
Enterococcus	LDL Chol	8.53836190481829e-05	-4.52E-05	0.000216	0.21196928	-0.112302	0.53624061	0.19243238	UnInfected	
Enterococcus	Glucose	7.90448594983811e-06	-7.43E-05	9.01074716403444e-05	0.03276687	-0.3079934	0.37352713	0.84595222	UnInfected	
Enterococcus	Systolic BP	0.00112002	-0.0001962	0.00243623	0.26240426	-0.0459637	0.57077221	0.0926666	UnInfected	
Akkermansia	Insulin	0.00074232	-0.0002888	0.00177346	0.19299434	-0.0750893	0.46107792	0.15229958	UnInfected	
Akkermansia	Diastolic BP	-0.0002654	-0.0016033	0.00107255	-0.0672824	-0.4065209	0.27195618	0.68890576	UnInfected	
Akkermansia	Total Chol	1.21367362006951e-05	-0.0001643	0.00018856	0.02210255	-0.2991894	0.34339451	0.88943858	UnInfected	
Akkermansia	LDL Chol	-1.48E-05	-0.0001843	0.00015458	-0.0291719	-0.3620859	0.30374216	0.85946493	UnInfected	
Akkermansia	Glucose	4.07112504676082e-05	-6.21E-05	0.00014357	0.13357947	-0.2039201	0.47107909	0.42607809	UnInfected	
Akkermansia	Systolic BP	-0.0011343	-0.0028246	0.00055598	-0.2103443	-0.5237906	0.10310199	0.18117603	UnInfected	

Rikenellaceae_RC9_gut_group	Insulin	0.0025331	-0.0005796	0.00564577	0.21686721	-0.0496194	0.48335386	0.10715941	UnInfected	
Rikenellaceae_RC9_gut_group	Diastolic BP	0.00014906	-0.0039348	0.00423296	0.0124462	-0.3285458	0.35343817	0.94119631	UnInfected	
Rikenellaceae_RC9_gut_group	Total Chol	0.0001451	-0.0003897	0.00067993	0.08701622	-0.2337157	0.40774818	0.58435647	UnInfected	
Rikenellaceae_RC9_gut_group	LDL Chol	0.0002461	-0.0002624	0.00075457	0.15924087	-0.1697797	0.48826141	0.33160531	UnInfected	
Rikenellaceae_RC9_gut_group	Glucose	0.0004092	0.00012923	0.00068916	0.44212483	0.13962966	0.74461999	0.00550598	UnInfected	**
Rikenellaceae_RC9_gut_group	Systolic BP	-0.0010757	-0.0063564	0.00420509	-0.0656863	-0.3881588	0.2567862	0.68097296	UnInfected	
Lachnospiraceae_NK4A136_group	Insulin	0.00055441	-0.002559	0.00366782	0.04982771	-0.2299912	0.32964664	0.71919946	UnInfected	
Lachnospiraceae_NK4A136_group	Diastolic BP	-0.001041	-0.0049515	0.00286949	-0.0912474	-0.4340145	0.25151978	0.59140412	UnInfected	
Lachnospiraceae_NK4A136_group	Total Chol	-0.0002439	-0.0007533	0.00026544	-0.1535517	-0.474213	0.16710951	0.33667576	UnInfected	
Lachnospiraceae_NK4A136_group	LDL Chol	-0.0001505	-0.000644	0.00034295	-0.1022508	-0.4374628	0.23296124	0.53878236	UnInfected	
Lachnospiraceae_NK4A136_group	Glucose	2.98051937829903e-05	-0.0002743	0.00033392	0.03380677	-0.3111342	0.37874776	0.84302959	UnInfected	
Lachnospiraceae_NK4A136_group	Systolic BP	-0.0021903	-0.0072218	0.00284118	-0.14041	-0.4629551	0.18213513	0.38184785	UnInfected	
Haemophilus	Insulin	-0.0003563	-0.0013575	0.00064499	-0.1016091	-0.3871812	0.18396287	0.47386605	UnInfected	
Haemophilus	Diastolic BP	0.00066788	-0.0005802	0.00191598	0.18578135	-0.1613967	0.5329594	0.28385325	UnInfected	
Haemophilus	Total Chol	-4.39E-05	-0.0002104	0.00012257	-0.087737	-0.4203516	0.24487769	0.59477625	UnInfected	
Haemophilus	LDL Chol	-3.97E-05	-0.0001996	0.00012034	-0.0854829	-0.4303756	0.25940978	0.61711556	UnInfected	
Haemophilus	Glucose	6.8613666229711e-06	-9.16E-05	0.00010529	0.02469785	-0.3295942	0.37898989	0.8879738	UnInfected	
Haemophilus	Systolic BP	0.00020948	-0.0014366	0.00185559	0.04261665	-0.2922632	0.37749656	0.79712651	UnInfected	
Ruminococcus_gauvreauii_group	Insulin	-0.0009718	-0.0039914	0.00204794	-0.0911709	-0.3744816	0.19213988	0.51683383	UnInfected	
Ruminococcus_gauvreauii_group	Diastolic BP	0.00080471	-0.0030121	0.00462152	0.07363175	-0.2756121	0.42287562	0.6704732	UnInfected	
Ruminococcus_gauvreauii_group	Total Chol	-0.000452	-0.0009286	2.45947132896986e-05	-0.2970546	-0.6102726	0.01616329	0.06227758	UnInfected	
Ruminococcus_gauvreauii_group	LDL Chol	-0.0003011	-0.0007725	0.00017031	-0.2135191	-0.547808	0.12076985	0.20253456	UnInfected	

Ruminococcus_gauvreauii_group	Glucose	-6.64E-05	-0.000362	0.0002291	-0.078667	-0.4286026	0.27126864	0.65011063	UnInfected	
Ruminococcus_gauvreauii_group	Systolic BP	0.00080466	-0.0041493	0.00575866	0.05384747	-0.2776737	0.38536865	0.74291465	UnInfected	
Lachnospiraceae_NK3A20_group	Insulin	0.00113282	-4.90E-05	0.00231459	0.25461037	-0.0110047	0.52022544	0.05966849	UnInfected	
Lachnospiraceae_NK3A20_group	Diastolic BP	-0.0010308	-0.0025607	0.00049901	-0.2259609	-0.561305	0.10938319	0.17944492	UnInfected	
Lachnospiraceae_NK3A20_group	Total Chol	-0.0001701	-0.000368	2.77438834831351e-05	-0.2678226	-0.5793241	0.0436788	0.08947309	UnInfected	
Lachnospiraceae_NK3A20_group	LDL Chol	-0.0001074	-0.0003025	8.77967879324536e-05	-0.1823638	-0.5138709	0.14914336	0.27082847	UnInfected	
Lachnospiraceae_NK3A20_group	Glucose	4.48693366797906e-05	-7.60E-05	0.00016574	0.12727348	-0.2155699	0.47011683	0.45507877	UnInfected	
Lachnospiraceae_NK3A20_group	Systolic BP	-0.0020791	-0.0039777	-0.0001805	-0.3333137	-0.637686	-0.0289415	0.03284309	UnInfected	*
Turicibacter	Insulin	-2.04E-05	-0.0017356	0.00169475	-0.0033174	-0.2820485	0.27541375	0.98080935	UnInfected	
Turicibacter	Diastolic BP	-0.0010164	-0.0031449	0.0011121	-0.1610907	-0.4984399	0.17625861	0.3380108	UnInfected	
Turicibacter	Total Chol	9.06157039152775e-05	-0.0001917	0.0003729	0.1031503	-0.2181771	0.4244777	0.5178594	UnInfected	
Turicibacter	LDL Chol	0.00013547	-0.0001331	0.00040401	0.16638797	-0.1634463	0.49622226	0.31186649	UnInfected	
Turicibacter	Glucose	0.0001105	-5.20E-05	0.00027299	0.22662747	-0.1066395	0.55989441	0.17558718	UnInfected	
Turicibacter	Systolic BP	-0.0013102	-0.0040701	0.0014497	-0.1518706	-0.4717811	0.16803998	0.34080105	UnInfected	
Alloprevotella	Insulin	0.00261653	-0.0025103	0.00774336	0.14641326	-0.1404693	0.43329578	0.3063318	UnInfected	
Alloprevotella	Diastolic BP	0.0009249	-0.0056304	0.0074802	0.0504751	-0.3072703	0.40822046	0.77566248	UnInfected	
Alloprevotella	Total Chol	9.87745843660959e-05	-0.0007641	0.00096165	0.03871566	-0.2994958	0.3769271	0.81711346	UnInfected	
Alloprevotella	LDL Chol	0.00010483	-0.0007238	0.00093346	0.04433529	-0.3061113	0.39478192	0.79829301	UnInfected	
Alloprevotella	Glucose	0.00025537	-0.0002447	0.00075542	0.18034012	-0.172796	0.53347625	0.30603177	UnInfected	
Alloprevotella	Systolic BP	-0.0002239	-0.0087329	0.0082852	-0.0089347	-0.3485525	0.33068307	0.95759674	UnInfected	
Fusicatenibacter	Insulin	-0.0015573	-0.0055751	0.00246042	-0.110603	-0.3959466	0.17474056	0.4356063	UnInfected	
Fusicatenibacter	Diastolic BP	-0.0017581	-0.0068268	0.00331071	-0.1217717	-0.4728581	0.22931474	0.48499316	UnInfected	
Fusicatenibacter	Total Chol	-0.0006001	-0.0012365	3.62544123946312e-05	-0.298557	-0.6151496	0.01803566	0.06369271	UnInfected	
Fusicatenibacter	LDL Chol	-0.0004596	-0.0010835	0.00016441	-0.2466774	-0.5816073	0.08825243	0.14336257	UnInfected	
Fusicatenibacter	Glucose	-6.52E-05	-0.0004602	0.0003298	-0.0584271	-0.4124564	0.29560211	0.73894104	UnInfected	
Fusicatenibacter	Systolic BP	-0.0023756	-0.0089425	0.00419127	-0.1203411	-0.4529986	0.21231645	0.4665674	UnInfected	
Butyricicoccus	Insulin	-0.0041469	-0.0097988	0.00150501	-0.20361	-0.4811152	0.07389526	0.14483383	UnInfected	

Butyricicoccus	Diastolic BP	0.00096729	-0.0063855	0.00832004	0.04631914	-0.3057704	0.39840873	0.7904432	UnInfected	
Butyricicoccus	Total Chol	-0.0005729	-0.0015192	0.00037336	-0.1970388	-0.5224859	0.12840819	0.22646809	UnInfected	
Butyricicoccus	LDL Chol	-0.0003116	-0.001235	0.00061188	-0.1156197	-0.4583059	0.22706651	0.49687896	UnInfected	
Butyricicoccus	Glucose	-2.03E-05	-0.0005904	0.00054987	-0.0125631	-0.3658553	0.34072909	0.94270783	UnInfected	
Butyricicoccus	Systolic BP	0.00378621	-0.0056589	0.01323133	0.13259749	-0.1981817	0.46337669	0.42023251	UnInfected	
UCG.010	Insulin	-0.0030598	-0.0105498	0.0044302	-0.1142637	-0.3939664	0.16543905	0.41150582	UnInfected	
UCG.010	Diastolic BP	0.01105401	0.00239194	0.01971607	0.4025882	0.08711472	0.71806168	0.01401014	UnInfected	*
UCG.010	Total Chol	0.00053391	-0.0007055	0.00177334	0.139659	-0.1845485	0.4638665	0.38678234	UnInfected	
UCG.010	LDL Chol	0.00041276	-0.0007827	0.00160825	0.11649866	-0.2209209	0.45391823	0.48696776	UnInfected	
UCG.010	Glucose	-6.26E-05	-0.0008007	0.00067548	-0.0295036	-0.3773519	0.31834464	0.86392217	UnInfected	
UCG.010	Systolic BP	0.00027824	-0.01208	0.01263645	0.00741111	-0.3217621	0.33658429	0.96370671	UnInfected	

Supplementary table 3: Associations between gut microbial taxa and cardiovascular disease (CVD) risk factors in the urban cohort. Associations were assessed using ordinary least squares linear regression models fitted separately for each microbial taxon and CVD trait. Unstandardised regression coefficients (β) and standardised coefficients (β_std) are shown with 95% confidence intervals and exact two-sided *P* values.β coefficients and confidence intervals were derived from the *t* statistic of the regression model. Models were adjusted for age, sex (categorical), and body mass index (BMI). Standardised coefficients were obtained by z-scaling continuous variables to facilitate comparison of effect sizes across outcomes.Analyses were conducted separately by *Schistosoma mansoni* infection status, as indicated in the group column. No adjustment was made for multiple comparisons. Positive coefficients indicate higher levels of the CVD risk factor with increasing microbial abundance, while negative coefficients indicate inverse associations.

microbe	cvdrisk	beta	beta_lci	beta_uci	beta_std	beta_std_lci	beta_std_uci	pvalue	group
Romboutsia	BP_Sys	-5.03E-05	-0.000401	0.00030037	-0.0415179	-0.331	0.24796417	0.77327877	Infected
Romboutsia	BP_Dia	5.12220920786206e-05	-0.0002118	0.00031421	0.05777044	-0.238833	0.35437384	0.69575185	Infected
Romboutsia	Chol_T	6.31231207770275e-06	-2.59E-05	3.85459591461656e-05	0.06277605	-0.2577881	0.38334023	0.69418763	Infected
Romboutsia	Chol_L	-3.20E-06	-2.99E-05	2.34457140678539e-05	-0.0382112	-0.355992	0.27956956	0.80911259	Infected
Romboutsia	insulin	3.86098094150695e-05	-0.0001572	0.00023441	0.06561455	-0.2671324	0.39836152	0.69217527	Infected
Romboutsia	glucose	-3.39E-06	-2.04E-05	1.35896498415212e-05	-0.0662138	-0.3974825	0.26505488	0.68820501	Infected
Clostridium_sensu_stricto_1	BP_Sys	9.34128687357808e-05	-0.0003551	0.00054195	0.06398946	-0.2432666	0.3712455	0.67588688	Infected
Clostridium_sensu_stricto_1	BP_Dia	0.00012782	-0.0002071	0.00046273	0.11962088	-0.1938102	0.43305199	0.44479132	Infected
Clostridium_sensu_stricto_1	Chol_T	3.94212303352634e-05	7.8335943835245e-08	7.87641247266916e-05	0.32531386	0.00064645	0.64998127	0.04956744	Infected
Clostridium_sensu_stricto_1	Chol_L	3.07099115199601e-05	-1.97E-06	6.33849586841119e-05	0.3038588	-0.019444	0.62716162	0.06470445	Infected
Clostridium_sensu_stricto_1	insulin	0.00028397	5.01524003539626e-05	0.0005178	0.40044949	0.07072306	0.73017591	0.01858356	Infected
Clostridium_sensu_stricto_1	glucose	4.60434347078268e-06	-1.71E-05	2.63499596877442e-05	0.07451816	-0.2774198	0.42645611	0.67080499	Infected
Succinivibrio	BP_Sys	0.00023305	4.54822095198633e-06	0.00046156	0.26717624	0.00521417	0.52913831	0.04582035	Infected
Succinivibrio	BP_Dia	9.74781291220984e-05	-8.04E-05	0.00027534	0.15267365	-0.1259002	0.43124748	0.27441616	Infected
Succinivibrio	Chol_T	-1.11E-05	-3.29E-05	1.07740477829518e-05	-0.1529551	-0.4547067	0.1487966	0.31154734	Infected
Succinivibrio	Chol_L	-1.76E-06	-2.00E-05	1.65150092851524e-05	-0.0291379	-0.3317484	0.27347259	0.84659057	Infected
Succinivibrio	insulin	-2.43E-05	-0.0001586	0.00010994	-0.0574207	-0.3742969	0.25945557	0.71595155	Infected

Succinivibrio	glucose	7.0230848629326e-06	-4.42E-06	1.84670529172172e-05	0.19022344	-0.1197416	0.5001885	0.22190513	Infected
Prevotella	BP_Sys	0.00011036	-0.0003936	0.00061434	0.06390684	-0.2279334	0.35574709	0.66026604	Infected
Prevotella	BP_Dia	-5.84E-05	-0.0004372	0.00032034	-0.0462353	-0.3459045	0.25343378	0.75664454	Infected
Prevotella	Chol_T	-5.34E-06	-5.18E-05	4.11111515399503e-05	-0.0372725	-0.3613364	0.2867914	0.81725623	Infected
Prevotella	Chol_L	-6.60E-06	-4.49E-05	3.17290212261272e-05	-0.0551839	-0.3757575	0.26538967	0.72957125	Infected
Prevotella	insulin	-0.0001205	-0.0004002	0.00015919	-0.143626	-0.4770187	0.18976659	0.38888032	Infected
Prevotella	glucose	-9.60E-06	-3.39E-05	1.46985745109535e-05	-0.1313388	-0.4637738	0.20109612	0.4290568	Infected
Intestinibacter	BP_Sys	-0.000156	-0.0005525	0.0002405	-0.1080733	-0.3827551	0.16660845	0.43095062	Infected
Intestinibacter	BP_Dia	0.00013613	-0.0001607	0.00043291	0.12883728	-0.1520527	0.40972731	0.35923954	Infected
Intestinibacter	Chol_T	1.75392663810489e-05	-1.88E-05	5.38746843293974e-05	0.14637342	-0.1568627	0.44960956	0.33490286	Infected
Intestinibacter	Chol_L	1.07205347139947e-05	-1.94E-05	4.08898593159824e-05	0.10727243	-0.1946096	0.40915446	0.47657693	Infected
Intestinibacter	insulin	-3.88E-05	-0.0002619	0.00018424	-0.0553389	-0.3734273	0.26274944	0.72681178	Infected
Intestinibacter	glucose	6.69697427671684e-07	-1.87E-05	2.00484857966271e-05	0.01096103	-0.3062143	0.32813637	0.94462989	Infected
Bifidobacterium	BP_Sys	7.89830473888248e-05	-0.0003208	0.00047876	0.05633231	-0.2287933	0.34145796	0.69161424	Infected
Bifidobacterium	BP_Dia	-0.0001299	-0.0004276	0.00016787	-0.1265507	-0.4166729	0.16357141	0.38302376	Infected
Bifidobacterium	Chol_T	-1.38E-05	-5.04E-05	2.27854273435182e-05	-0.1185704	-0.4329132	0.19577242	0.45007731	Infected
Bifidobacterium	Chol_L	-8.41E-06	-3.87E-05	2.19078719526782e-05	-0.0865899	-0.3988713	0.2256916	0.57810494	Infected
Bifidobacterium	insulin	-6.52E-05	-0.0002881	0.00015769	-0.0957363	-0.4229949	0.23152242	0.55745417	Infected
Bifidobacterium	glucose	-1.19E-05	-3.09E-05	7.14295106680614e-06	-0.200417	-0.5211972	0.12036328	0.21382663	Infected
Blautia	BP_Sys	-0.0001089	-0.0010822	0.00086447	-0.0310372	-0.3085234	0.246449	0.82219587	Infected
Blautia	BP_Dia	-7.06E-05	-0.0008014	0.00066016	-0.027499	-0.312117	0.25711911	0.84607377	Infected
Blautia	Chol_T	-1.42E-05	-0.0001037	7.52633922443236e-05	-0.0488791	-0.3562394	0.25848132	0.74942385	Infected
Blautia	Chol_L	-1.06E-05	-8.45E-05	6.33625560849645e-05	-0.0434741	-0.347863	0.26091493	0.7741965	Infected
Blautia	insulin	-9.83E-05	-0.0006417	0.00044518	-0.0576632	-0.37659	0.26126368	0.71655761	Infected
Blautia	glucose	-2.52E-05	-7.17E-05	2.13324621912609e-05	-0.1696079	-0.4828998	0.14368398	0.28021973	Infected
Faecalibacterium	BP_Sys	-0.000305	-0.0012997	0.00068975	-0.0862793	-0.3676853	0.19512676	0.53876054	Infected

Faecalibacterium	BP_Dia	-0.0001313	-0.0008805	0.00061781	-0.0507647	-0.3403084	0.23877892	0.72477608	Infected
Faecalibacterium	Chol_T	1.23945188500605 e-05	-7.95E-05	0.00010428	0.04224017	-0.2709061	0.35538647	0.7864158	Infected
Faecalibacterium	Chol_L	-3.57E-05	-0.0001108	3.93673475725873 e-05	-0.1458567	-0.4525753	0.16086184	0.34204157	Infected
Faecalibacterium	insulin	2.87590604470919 e-05	-0.0005299	0.00058738	0.01674818	-0.3085729	0.34206929	0.91759814	Infected
Faecalibacterium	glucose	-8.39E-06	-5.68E-05	4.00020614677739 e-05	-0.0560671	-0.3794966	0.26736243	0.72774765	Infected
Dialister	BP_Sys	0.00048116	-0.0002529	0.00121522	0.18069452	-0.0949739	0.45636295	0.19260999	Infected
Dialister	BP_Dia	0.00014642	-0.0004149	0.00070772	0.07512138	-0.21286	0.36310282	0.60074606	Infected
Dialister	Chol_T	-2.15E-05	-9.02E-05	4.72042285135155 e-05	-0.097217	-0.4079872	0.21355319	0.53058937	Infected
Dialister	Chol_L	-8.34E-06	-6.53E-05	4.86078381942436 e-05	-0.04525	-0.354165	0.26366491	0.76858457	Infected
Dialister	insulin	-2.26E-05	-0.0004419	0.0003968	-0.0174387	-0.3416309	0.30675354	0.91391662	Infected
Dialister	glucose	2.34414560415414 e-05	-1.21E-05	5.90245794659671 e-05	0.20798487	-0.1077272	0.52369696	0.19042675	Infected
Subdoligranulum	BP_Sys	-0.0002702	-0.0013462	0.00080591	-0.069858	-0.3481072	0.20839126	0.61443843	Infected
Subdoligranulum	BP_Dia	0.00011605	-0.0006935	0.00092559	0.04099755	-0.2449894	0.32698453	0.77338141	Infected
Subdoligranulum	Chol_T	3.68248549354159 e-05	-6.18E-05	0.00013544	0.11471101	-0.1924801	0.42190215	0.45459988	Infected
Subdoligranulum	Chol_L	2.50908166959145 e-05	-5.65E-05	0.00010671	0.09371297	-0.2111397	0.39856563	0.53770227	Infected
Subdoligranulum	insulin	-8.69E-05	-0.0006897	0.00051582	-0.0462732	-0.3671218	0.27457531	0.77205035	Infected
Subdoligranulum	glucose	5.74088440462542 e-06	-4.66E-05	5.80531617691588 e-05	0.03507235	-0.2845151	0.35465977	0.82549139	Infected
Terrisporobacter	BP_Sys	-0.0011042	-0.0020166	-0.0001918	-0.3238722	-0.5914869	-0.0562574	0.01897281	Infected
Terrisporobacter	BP_Dia	-0.0005876	-0.0012982	0.00012298	-0.23546	-0.5202021	0.04928213	0.10240874	Infected
Terrisporobacter	Chol_T	9.02324892146569 e-08	-9.01E-05	9.02566599373761 e-05	0.00031883	-0.3182794	0.31891711	0.99839526	Infected
Terrisporobacter	Chol_L	4.051284198235e- 06	-7.04E-05	7.84947274842155 e-05	0.01716375	-0.2982248	0.33255227	0.91291297	Infected
Terrisporobacter	insulin	-9.60E-05	-0.0006429	0.00045081	-0.0579808	-0.3881628	0.27220124	0.72436148	Infected
Terrisporobacter	glucose	-1.02E-05	-5.76E-05	3.71913206342202 e-05	-0.0707383	-0.3992049	0.25772833	0.66552493	Infected
Collinsella	BP_Sys	-0.0003382	-0.0015428	0.00086654	-0.0783905	-0.3576617	0.20088066	0.5734504	Infected
Collinsella	BP_Dia	-0.0004197	-0.0013175	0.00047813	-0.132913	-0.4172551	0.15142905	0.35023117	Infected
Collinsella	Chol_T	-9.66E-05	-0.0002034	1.02505957092977 e-05	-0.2696701	-0.5679668	0.02862662	0.07511806	Infected

Collinsella	Chol_L	-7.29E-05	-0.0001617	1.58977163585844e-05	-0.2441698	-0.541572	0.05323246	0.10480462	Infected
Collinsella	insulin	-0.0001665	-0.0008405	0.00050739	-0.0794767	-0.4010909	0.24213748	0.61999383	Infected
Collinsella	glucose	-2.86E-05	-8.65E-05	2.93100321969521e-05	-0.1566631	-0.4738574	0.16053112	0.32394689	Infected
Escherichia.Shigella	BP_Sys	-0.0004428	-0.0030005	0.00211497	-0.0491223	-0.3328911	0.23464638	0.72811623	Infected
Escherichia.Shigella	BP_Dia	-0.0002446	-0.0021659	0.00167672	-0.0370767	-0.3282999	0.25414651	0.7981343	Infected
Escherichia.Shigella	Chol_T	2.84014179749021e-05	-0.0002071	0.00026392	0.03795951	-0.2768241	0.35274311	0.80857546	Infected
Escherichia.Shigella	Chol_L	3.21552528396692e-05	-0.0001622	0.00022651	0.0515292	-0.2599222	0.36298063	0.73968131	Infected
Escherichia.Shigella	insulin	0.00028433	-0.0011445	0.00171315	0.06493877	-0.2613891	0.39126662	0.68950467	Infected
Escherichia.Shigella	glucose	-1.54E-05	-0.0001395	0.00010871	-0.0403547	-0.3656626	0.28495316	0.80319599	Infected
Sarcina	BP_Sys	0.00050227	-0.0008359	0.00184045	0.10432732	-0.1736304	0.38228506	0.4523051	Infected
Sarcina	BP_Dia	0.00064111	-0.0003493	0.00163147	0.18193133	-0.0991106	0.46297325	0.19806741	Infected
Sarcina	Chol_T	0.00013624	2.03200457056076e-05	0.00025215	0.34089844	0.05084623	0.63095065	0.02244205	Infected
Sarcina	Chol_L	0.00011162	1.57961533653314e-05	0.00020745	0.33488924	0.04739213	0.62238634	0.02358717	Infected
Sarcina	insulin	0.00039515	-0.0003473	0.00113763	0.16896395	-0.1485147	0.48644259	0.28832344	Infected
Sarcina	glucose	4.72053024311509e-06	-6.06E-05	7.00619082643423e-05	0.02316577	-0.2974939	0.34382543	0.88457343	Infected
Holdemanella	BP_Sys	-8.49E-05	-0.0007794	0.00060956	-0.0349731	-0.3210496	0.25110338	0.80599194	Infected
Holdemanella	BP_Dia	4.52987233744679e-05	-0.0004762	0.00056679	0.02549401	-0.2680001	0.3189881	0.86143982	Infected
Holdemanella	Chol_T	-1.59E-05	-7.97E-05	4.77948780121929e-05	-0.0791129	-0.3954127	0.23718696	0.61576408	Infected
Holdemanella	Chol_L	-1.59E-05	-6.85E-05	3.66236945417188e-05	-0.0947672	-0.4074521	0.21791768	0.54341619	Infected
Holdemanella	insulin	-4.91E-05	-0.0004373	0.00033897	-0.0416718	-0.3708006	0.28745686	0.79922106	Infected
Holdemanella	glucose	-4.73E-06	-3.84E-05	2.89319218679663e-05	-0.0460366	-0.3736576	0.28158449	0.77774249	Infected
Bacteroides	BP_Sys	-0.002384	-0.0060962	0.00132824	-0.1891444	-0.4836721	0.10538336	0.20158423	Infected
Bacteroides	BP_Dia	-0.0012229	-0.0040414	0.00159561	-0.1325547	-0.4380648	0.17295547	0.38553281	Infected
Bacteroides	Chol_T	0.0002176	-0.0001241	0.00055927	0.20798464	-0.1185819	0.53455115	0.20526107	Infected
Bacteroides	Chol_L	0.00015158	-0.0001323	0.00043544	0.17371103	-0.1515933	0.49901538	0.28672869	Infected
Bacteroides	insulin	0.00176631	-0.0002742	0.00380682	0.28848943	-0.0447837	0.62176256	0.08782769	Infected

Bacteroides	glucose	7.00371450719916e-05	-0.0001124	0.00025247	0.13128522	-0.2106867	0.47325713	0.44212391	Infected
Streptococcus	BP_Sys	-8.74E-05	-0.0008461	0.00067127	-0.0328391	-0.3178842	0.25220598	0.81695872	Infected
Streptococcus	BP_Dia	-0.0001418	-0.0007098	0.00042627	-0.0727706	-0.3643455	0.2188044	0.61652728	Infected
Streptococcus	Chol_T	-3.63E-05	-0.0001052	3.25164372129177e-05	-0.1644709	-0.4761151	0.14717325	0.29231877	Infected
Streptococcus	Chol_L	-3.91E-05	-9.54E-05	1.71383570540937e-05	-0.2123697	-0.5177465	0.09300708	0.16745015	Infected
Streptococcus	insulin	-9.34E-05	-0.0005167	0.00032984	-0.0722475	-0.3996012	0.25510614	0.65776899	Infected
Streptococcus	glucose	2.56773868697126e-06	-3.42E-05	3.93684031887491e-05	0.02279286	-0.3038729	0.3494586	0.88849235	Infected
Clostridia_UCG.014	BP_Sys	-0.0010913	-0.0031041	0.00092148	-0.161251	-0.4586618	0.13615982	0.27951218	Infected
Clostridia_UCG.014	BP_Dia	-0.0012558	-0.0027349	0.00022324	-0.2535206	-0.5521069	0.04506561	0.0938373	Infected
Clostridia_UCG.014	Chol_T	3.39371602947981e-05	-0.0001538	0.00022164	0.06040992	-0.2737125	0.39453235	0.71656061	Infected
Clostridia_UCG.014	Chol_L	8.42955484011251e-05	-6.85E-05	0.00023714	0.17991123	-0.1462994	0.50612191	0.27144094	Infected
Clostridia_UCG.014	insulin	-0.0001142	-0.0012558	0.00102733	-0.0347495	-0.3819915	0.31249253	0.84064355	Infected
Clostridia_UCG.014	glucose	-3.24E-05	-0.000131	6.60762817573297e-05	-0.113274	-0.457223	0.23067491	0.50924265	Infected
Agathobacter	BP_Sys	0.00039282	-0.0024645	0.00325009	0.03882511	-0.2435805	0.32123074	0.78242041	Infected
Agathobacter	BP_Dia	-0.0002851	-0.00243	0.00185986	-0.0384947	-0.3281292	0.2511398	0.78947641	Infected
Agathobacter	Chol_T	0.000136	-0.0001234	0.00039544	0.16192618	-0.1469823	0.47083463	0.29554379	Infected
Agathobacter	Chol_L	8.32648015004274e-05	-0.0001324	0.00029888	0.11887069	-0.1889513	0.42669272	0.43946171	Infected
Agathobacter	insulin	-0.0002616	-0.0018579	0.00133468	-0.0532277	-0.3780163	0.27156098	0.74205167	Infected
Agathobacter	glucose	-5.93E-05	-0.0001966	7.80843827561721e-05	-0.1383643	-0.4590672	0.18233861	0.3881837	Infected
Pseudomonas	BP_Sys	-0.0001228	-0.0003574	0.00011185	-0.1514852	-0.4409851	0.1380147	0.29638316	Infected
Pseudomonas	BP_Dia	-5.35E-05	-0.0002313	0.00012427	-0.0902096	-0.3899136	0.20949443	0.54617331	Infected
Pseudomonas	Chol_T	1.54231450890372e-06	-2.03E-05	2.34302403140871e-05	0.02292715	-0.302446	0.34830032	0.88739737	Infected
Pseudomonas	Chol_L	2.9342729717676e-08	-1.80E-05	1.81080197750454e-05	0.000523	-0.321706	0.32275201	0.99739733	Infected
Pseudomonas	insulin	-4.18E-05	-0.0001741	9.04532135664226e-05	-0.1063045	-0.4423815	0.22977251	0.52604534	Infected
Pseudomonas	glucose	-5.21E-06	-1.66E-05	6.20470960793188e-06	-0.1518464	-0.4845853	0.18089251	0.36165122	Infected
Turicibacter	BP_Sys	-0.0015932	-0.0034554	0.00026889	-0.2353384	-0.5103939	0.03971709	0.09142586	Infected

Turicibacter	BP_Dia	-0.001194	-0.002592	0.00020393	-0.2409601	-0.5230727	0.04115256	0.09196614	Infected
Turicibacter	Chol_T	-2.87E-05	-0.0002062	0.00014885	-0.0510548	-0.3669842	0.26487452	0.74551331	Infected
Turicibacter	Chol_L	-4.34E-06	-0.0001511	0.00014247	-0.009251	-0.3224599	0.30395789	0.95266567	Infected
Turicibacter	insulin	-0.0002553	-0.0013321	0.0008215	-0.0776347	-0.4050647	0.24979525	0.63419989	Infected
Turicibacter	glucose	-1.80E-05	-0.0001115	7.55329292782869e-05	-0.0627252	-0.3890468	0.26359634	0.69953895	Infected
Roseburia	BP_Sys	0.00202789	-0.0012726	0.00532842	0.17479005	-0.1096916	0.45927173	0.22137169	Infected
Roseburia	BP_Dia	0.00147208	-0.0010086	0.00395279	0.17334754	-0.1187714	0.46546644	0.23726562	Infected
Roseburia	Chol_T	0.00024185	-5.77E-05	0.00054141	0.2511225	-0.0599219	0.56216685	0.11051262	Infected
Roseburia	Chol_L	0.00020941	-3.71E-05	0.00045593	0.26071334	-0.0461952	0.56762189	0.09368363	Infected
Roseburia	insulin	0.00205375	0.00029444	0.00381306	0.36440717	0.05224432	0.67657002	0.02330664	Infected
Roseburia	glucose	-5.82E-06	-0.000169	0.00015733	-0.0118577	-0.3441021	0.32038668	0.94282024	Infected
uncultured	BP_Sys	-0.0007433	-0.0054962	0.00400973	-0.0447773	-0.33112	0.24156534	0.75346028	Infected
uncultured	BP_Dia	-0.001928	-0.0054454	0.00158937	-0.1586876	-0.4481903	0.13081502	0.27434352	Infected
uncultured	Chol_T	0.00022096	-0.000211	0.00065295	0.16036494	-0.1531511	0.47388095	0.30722131	Infected
uncultured	Chol_L	0.00036401	2.22018398623898e-05	0.00070583	0.3167595	0.01931966	0.61419933	0.03747282	Infected
uncultured	insulin	-0.0005523	-0.0032062	0.00210153	-0.0685002	-0.3976292	0.26062883	0.67608513	Infected
uncultured	glucose	-0.000155	-0.0003802	7.02502288137924e-05	-0.2205737	-0.5411381	0.09999067	0.17187886	Infected
Lactobacillus	BP_Sys	0.00043415	-0.0011487	0.00201701	0.07569387	-0.200273	0.35166068	0.58220229	Infected
Lactobacillus	BP_Dia	-5.79E-05	-0.0012506	0.00113475	-0.013802	-0.297894	0.27028997	0.92222265	Infected
Lactobacillus	Chol_T	-0.0001119	-0.0002535	2.9771006110809e-05	-0.2349774	-0.5324837	0.06252885	0.11821091	Infected
Lactobacillus	Chol_L	-0.000113	-0.0002281	2.01986820244738e-06	-0.2846392	-0.574365	0.00508664	0.05395843	Infected
Lactobacillus	insulin	-0.0003803	-0.0012599	0.00049927	-0.1364925	-0.4521782	0.17919324	0.38717427	Infected
Lactobacillus	glucose	-2.38E-05	-0.0001005	5.2814383495236e-05	-0.0982183	-0.4139876	0.21755102	0.53292124	Infected
Lachnospiraceae_NK4A136_group	BP_Sys	-0.0006199	-0.0036006	0.00236077	-0.0606151	-0.3520685	0.23083819	0.67630509	Infected
Lachnospiraceae_NK4A136_group	BP_Dia	-5.08E-05	-0.0022933	0.0021916	-0.0067926	-0.3063563	0.2927711	0.96365223	Infected
Lachnospiraceae_NK4A136_group	Chol_T	-3.57E-05	-0.0003104	0.00023889	-0.0420933	-0.3655855	0.2813989	0.79378546	Infected

Lachnospiraceae_ NK4A136_group	Chol_L	-5.65E-06	-0.0002326	0.00022132	-0.0079785	-0.3285352	0.31257827	0.96010552	Infected
Lachnospiraceae_ NK4A136_group	insulin	-0.0003007	-0.0019676	0.00136616	-0.0605275	-0.396049	0.27499398	0.71716488	Infected
Lachnospiraceae_ NK4A136_group	glucose	0.00011227	-2.79E-05	0.00025248	0.2593713	-0.064531	0.58327363	0.11335388	Infected
Comamonas	BP_Sys	-0.000303	-0.0023135	0.00170759	-0.0417536	-0.3188326	0.23532543	0.76213673	Infected
Comamonas	BP_Dia	0.00023804	-0.001271	0.00174709	0.04481683	-0.239304	0.32893764	0.75138635	Infected
Comamonas	Chol_T	0.00022802	5.81719419341172 e-05	0.00039787	0.37855295	0.09657587	0.66053004	0.0098111	Infected
Comamonas	Chol_L	0.00017342	3.11733671196575 e-05	0.00031567	0.34520673	0.06205255	0.62836091	0.01816751	Infected
Comamonas	insulin	0.00052804	-0.0005839	0.00164003	0.14980281	-0.1656625	0.46526817	0.34272056	Infected
Comamonas	glucose	-7.85E-05	-0.0001727	1.57267412362372 e-05	-0.2555963	-0.562398	0.0512054	0.09995579	Infected
Klebsiella	BP_Sys	-0.0031044	-0.0113381	0.00512923	-0.1070915	-0.3911224	0.17693935	0.45026702	Infected
Klebsiella	BP_Dia	-0.0008878	-0.0071076	0.00533206	-0.0418401	-0.3349734	0.25129325	0.77433483	Infected
Klebsiella	Chol_T	-0.0001499	-0.0009116	0.00061172	-0.0623056	-0.3788219	0.25421075	0.69268177	Infected
Klebsiella	Chol_L	-0.0003614	-0.0009806	0.00025787	-0.1800592	-0.4886085	0.12849016	0.24500032	Infected
Klebsiella	insulin	0.00016648	-0.0044693	0.00480222	0.01182198	-0.3173773	0.34102128	0.94246586	Infected
Klebsiella	glucose	0.0002111	-0.0001852	0.00060742	0.17204907	-0.1509566	0.4950547	0.28792666	Infected
Senegalimassilia	BP_Sys	-8.03E-06	-0.0042695	0.00425346	-0.0005255	-0.2794559	0.27840494	0.99697912	Infected
Senegalimassilia	BP_Dia	-0.0005375	-0.0037316	0.00265662	-0.048066	-0.3336939	0.23756188	0.73539532	Infected
Senegalimassilia	Chol_T	9.66106216379131 e-05	-0.0002942	0.00048745	0.07617808	-0.2320027	0.38435886	0.61989756	Infected
Senegalimassilia	Chol_L	0.00015897	-0.0001607	0.00047862	0.15029554	-0.1519086	0.45249969	0.32064178	Infected
Senegalimassilia	insulin	0.00078638	-0.0015817	0.0031545	0.10595763	-0.2131276	0.4250429	0.5057542	Infected
Senegalimassilia	glucose	1.28228745276256 e-06	-0.0002053	0.0002079	0.00198295	-0.3175339	0.32149985	0.9900484	Infected
Christensenellacea e_R.7_group	BP_Sys	-0.0022815	-0.0052636	0.00070056	-0.2091703	-0.4825685	0.0642278	0.12981711	Infected
Christensenellacea e_R.7_group	BP_Dia	-0.0020134	-0.0042254	0.0001987	-0.2521819	-0.5292517	0.02488792	0.07323781	Infected
Christensenellacea e_R.7_group	Chol_T	-7.32E-05	-0.0003549	0.0002085	-0.080819	-0.3919198	0.2302819	0.60223684	Infected
Christensenellacea e_R.7_group	Chol_L	-4.22E-06	-0.0002376	0.00022919	-0.0055893	-0.3146869	0.30350827	0.97100999	Infected
Christensenellacea e_R.7_group	insulin	4.0759751100326e -05	-0.0016763	0.00175781	0.0076927	-0.3163719	0.33175728	0.96194935	Infected

Christensenellaceae_R.7_group	glucose	6.96846854594678e-05	-7.76E-05	0.00021692	0.15094186	-0.1679852	0.46986893	0.34431311	Infected
Gastranaerophilales	BP_Sys	-0.0004107	-0.003771	0.00294963	-0.0340381	-0.3125579	0.24448166	0.80605418	Infected
Gastranaerophilales	BP_Dia	7.92148883540472e-05	-0.002445	0.00260346	0.00897012	-0.2768695	0.29480974	0.94971217	Infected
Gastranaerophilales	Chol_T	-0.0001004	-0.0004081	0.00020728	-0.1002692	-0.407511	0.20697268	0.513063	Infected
Gastranaerophilales	Chol_L	3.28061276979622e-06	-0.0002522	0.00025878	0.00392758	-0.3019582	0.3098133	0.97941261	Infected
Gastranaerophilales	insulin	0.00057635	-0.0012939	0.00244662	0.09833985	-0.2207785	0.41745822	0.53670632	Infected
Gastranaerophilales	glucose	0.00014761	-8.28E-06	0.00030349	0.28905207	-0.0162112	0.59431535	0.06281142	Infected
Coprococcus	BP_Sys	0.0012894	-0.0030308	0.00560963	0.08469571	-0.1990833	0.36847474	0.5495483	Infected
Coprococcus	BP_Dia	-0.0006948	-0.0039451	0.00255547	-0.0623536	-0.3540361	0.22932883	0.66783571	Infected
Coprococcus	Chol_T	5.00502076073632e-07	-0.0003988	0.00039985	0.00039605	-0.3156112	0.31640328	0.99799027	Infected
Coprococcus	Chol_L	8.22942803633855e-05	-0.0002464	0.00041098	0.07807911	-0.2337694	0.38992758	0.61540489	Infected
Coprococcus	insulin	0.00034534	-0.0020779	0.00276863	0.0466971	-0.2809801	0.37437426	0.7746798	Infected
Coprococcus	glucose	8.77332588190523e-05	-0.0001208	0.00029625	0.13615406	-0.1874418	0.45974996	0.3999347	Infected
Dorea	BP_Sys	0.00142556	-0.0047455	0.00759665	0.06468531	-0.2153292	0.34469979	0.64291714	Infected
Dorea	BP_Dia	-0.0005449	-0.0051868	0.004097	-0.033779	-0.3215374	0.25397927	0.81356045	Infected
Dorea	Chol_T	-0.0002411	-0.0008051	0.00032293	-0.1317738	-0.4400695	0.17652191	0.39257149	Infected
Dorea	Chol_L	-0.000219	-0.0006838	0.00024579	-0.1435301	-0.4481515	0.16109135	0.34643872	Infected
Dorea	insulin	0.00077042	-0.0026793	0.00422012	0.07196372	-0.2502677	0.3941951	0.6539685	Infected
Dorea	glucose	-3.83E-05	-0.0003381	0.00026143	-0.041111	-0.3624911	0.2802692	0.79719451	Infected
Treponema	BP_Sys	0.00030189	-0.0004687	0.00107243	0.10731707	-0.1665999	0.38123409	0.4328847	Infected
Treponema	BP_Dia	-1.53E-06	-0.0005846	0.00058152	-0.0007421	-0.283905	0.28242088	0.99579762	Infected
Treponema	Chol_T	-2.47E-05	-9.57E-05	4.63290066540914e-05	-0.1057218	-0.4098448	0.19840127	0.48614226	Infected
Treponema	Chol_L	-3.03E-05	-8.85E-05	2.7852965264128e-05	-0.1557633	-0.4545423	0.14301571	0.29814552	Infected
Treponema	insulin	-0.0001323	-0.0005643	0.00029971	-0.0968056	-0.4129391	0.21932786	0.53926561	Infected
Treponema	glucose	-7.00E-06	-4.46E-05	3.05946780264191e-05	-0.0587613	-0.374479	0.25695634	0.7086132	Infected
UCG.002	BP_Sys	-0.0032176	-0.0123187	0.00588345	-0.1067138	-0.4085554	0.19512785	0.47880235	Infected

UCG.002	BP_Dia	-0.0040972	-0.0108442	0.00264988	-0.1856457	-0.4913594	0.12006809	0.22670089	Infected
UCG.002	Chol_T	0.00055673	-0.0002666	0.00138006	0.22243437	-0.1065167	0.55138545	0.17922714	Infected
UCG.002	Chol_L	0.00049311	-0.0001843	0.00117051	0.23622279	-0.0882868	0.56073241	0.14893965	Infected
UCG.002	insulin	0.0011126	-0.0039946	0.0062198	0.07596136	-0.2727273	0.42465002	0.66190614	Infected
UCG.002	glucose	-0.0001416	-0.0005834	0.00030017	-0.110954	-0.4571156	0.23520761	0.52057321	Infected
Ruminococcus_torques_group	BP_Sys	-0.000287	-0.0043836	0.00380966	-0.0198039	-0.3025107	0.26290289	0.88805307	Infected
Ruminococcus_torques_group	BP_Dia	-0.0006989	-0.0037665	0.00236865	-0.0658948	-0.3551076	0.22331793	0.64746299	Infected
Ruminococcus_torques_group	Chol_T	-0.0002861	-0.0006515	7.94016422472155e-05	-0.2378116	-0.5416332	0.06600999	0.12144584	Infected
Ruminococcus_torques_group	Chol_L	-0.000216	-0.0005193	8.7395995119272e-05	-0.2152742	-0.5176634	0.08711493	0.15785372	Infected
Ruminococcus_torques_group	insulin	3.2509002035609e-05	-0.0022577	0.00232272	0.00461829	-0.3207324	0.32996895	0.97724097	Infected
Ruminococcus_torques_group	glucose	-7.95E-05	-0.0002765	0.00011746	-0.1296777	-0.4508698	0.19151451	0.4190957	Infected
Ruminococcus	BP_Sys	-0.001578	-0.0069985	0.00384247	-0.0808024	-0.3583566	0.19675184	0.55935631	Infected
Ruminococcus	BP_Dia	-0.0004528	-0.0045371	0.00363152	-0.031674	-0.3173929	0.2540449	0.82374895	Infected
Ruminococcus	Chol_T	0.00054815	7.97270001020492e-05	0.00101658	0.33812763	0.0491796	0.62707567	0.02299394	Infected
Ruminococcus	Chol_L	0.00050502	0.00012509	0.00088496	0.37351878	0.09251406	0.65452349	0.01049894	Infected
Ruminococcus	insulin	0.00324168	0.00038555	0.00609781	0.3417013	0.04064083	0.64276177	0.02715157	Infected
Ruminococcus	glucose	-0.0001154	-0.0003767	0.00014591	-0.1396213	-0.4557546	0.17651194	0.37716099	Infected
Sutterella	BP_Sys	0.00439013	-0.002321	0.01110122	0.17879688	-0.094526	0.45211976	0.19348652	Infected
Sutterella	BP_Dia	0.00128419	-0.0038485	0.00641685	0.07145383	-0.2141336	0.35704122	0.61565197	Infected
Sutterella	Chol_T	0.00034506	-0.0002761	0.00096627	0.1692963	-0.1354872	0.47407981	0.26808014	Infected
Sutterella	Chol_L	0.000272	-0.0002417	0.0007857	0.16000958	-0.1421831	0.46220227	0.29075056	Infected
Sutterella	insulin	0.00069733	-0.0031302	0.00452482	0.05846408	-0.2624325	0.37936061	0.71448318	Infected
Sutterella	glucose	2.25851581468453e-05	-0.0003099	0.00035511	0.02173195	-0.2982351	0.341699	0.89143739	Infected
Ruminobacter	BP_Sys	0.00234875	-0.0014404	0.00613795	0.17176445	-0.1053392	0.44886814	0.2173866	Infected
Ruminobacter	BP_Dia	0.0023487	-0.0004509	0.00514829	0.23465916	-0.0450476	0.51436594	0.0976782	Infected
Ruminobacter	Chol_T	0.00042145	9.30989190956287e-05	0.0007498	0.37128816	0.08201833	0.660558	0.01322233	Infected

Ruminobacter	Chol_L	0.0003123	3.66352844227396e-05	0.00058795	0.32987725	0.03869784	0.62105665	0.0274186	Infected
Ruminobacter	insulin	-0.000119	-0.0022787	0.00204073	-0.0179151	-0.3430489	0.30721875	0.91183001	Infected
Ruminobacter	glucose	-8.69E-05	-0.0002722	9.83249018139453e-05	-0.1501994	-0.4702825	0.16988373	0.3483891	Infected
Eubacterium_hallii_group	BP_Sys	-0.0058584	-0.0145434	0.00282661	-0.1829503	-0.4541722	0.08827167	0.18026737	Infected
Eubacterium_hallii_group	BP_Dia	-0.0045548	-0.0110628	0.00195321	-0.1943298	-0.4719926	0.08333314	0.16481942	Infected
Eubacterium_hallii_group	Chol_T	0.00019643	-0.000619	0.0010119	0.07390008	-0.2328814	0.38068156	0.62881398	Infected
Eubacterium_hallii_group	Chol_L	0.00019113	-0.0004814	0.0008637	0.08621176	-0.2171669	0.38959044	0.56873298	Infected
Eubacterium_hallii_group	insulin	-0.0011929	-0.0061465	0.00376064	-0.0766897	-0.3951396	0.24176015	0.62890816	Infected
Eubacterium_hallii_group	glucose	0.00011852	-0.0003108	0.00054783	0.08744718	-0.2293048	0.40419921	0.57975412	Infected
Methanobrevibacter	BP_Sys	0.00195092	-0.0011524	0.00505419	0.18083926	-0.1068169	0.46849544	0.2110485	Infected
Methanobrevibacter	BP_Dia	-0.001071	-0.0034228	0.00128081	-0.1356308	-0.4334621	0.16220058	0.36264966	Infected
Methanobrevibacter	Chol_T	-0.0001628	-0.0004494	0.0001238	-0.1817648	-0.5017756	0.13824593	0.25760294	Infected
Methanobrevibacter	Chol_L	-2.94E-05	-0.0002698	0.00021106	-0.0393199	-0.3612209	0.28258119	0.80615051	Infected
Methanobrevibacter	insulin	-0.0001541	-0.0019234	0.00161523	-0.029406	-0.3670279	0.30821589	0.86107099	Infected
Methanobrevibacter	glucose	1.07276377448439e-05	-0.0001428	0.00016424	0.02349366	-0.3126947	0.35968199	0.8883206	Infected
Haemophilus	BP_Sys	-0.0002016	-0.0043918	0.00398853	-0.0136252	-0.2967782	0.26952783	0.92296198	Infected
Haemophilus	BP_Dia	0.00024731	-0.0028974	0.00339199	0.02283194	-0.2674914	0.31315528	0.87443404	Infected
Haemophilus	Chol_T	-8.32E-05	-0.0004678	0.00030147	-0.0676987	-0.3808172	0.24541984	0.6642903	Infected
Haemophilus	Chol_L	5.72609323830657e-06	-0.0003127	0.00032411	0.00558909	-0.3051769	0.31635511	0.97116664	Infected
Haemophilus	insulin	0.00015216	-0.0021895	0.00249384	0.02116674	-0.3045845	0.346918	0.89611013	Infected
Haemophilus	glucose	8.01946190852834e-05	-0.0001213	0.00028171	0.12803511	-0.1936973	0.4497675	0.42573418	Infected
Akkermansia	BP_Sys	0.00137988	-0.012556	0.0153158	0.02787175	-0.2536155	0.30935901	0.84230326	Infected
Akkermansia	BP_Dia	-0.0004432	-0.0109086	0.01002211	-0.012231	-0.3010262	0.27656419	0.93217138	Infected
Akkermansia	Chol_T	9.10308712272898e-05	-0.0011915	0.00137357	0.02215045	-0.2899284	0.33422926	0.88658342	Infected
Akkermansia	Chol_L	0.00020689	-0.0008503	0.0012641	0.06035938	-0.248086	0.36880479	0.69439615	Infected
Akkermansia	insulin	0.00051701	-0.0072741	0.00830812	0.02149777	-0.3024612	0.34545671	0.89391507	Infected

Akkermansia	glucose	0.00020498	-0.0004678	0.00087774	0.09782101	-0.2232316	0.41887363	0.5412836	Infected
Catenibacterium	BP_Sys	0.00196883	-0.0041573	0.00809501	0.08971161	-0.1894328	0.36885605	0.51946861	Infected
Catenibacterium	BP_Dia	-0.0001911	-0.0048142	0.00443189	-0.0118983	-0.2996905	0.27589388	0.93378232	Infected
Catenibacterium	Chol_T	0.00017526	-0.0003886	0.00073912	0.09620527	-0.213306	0.40571649	0.53320264	Infected
Catenibacterium	Chol_L	0.00014633	-0.0003192	0.00061188	0.09630636	-0.2100996	0.40271234	0.52865174	Infected
Catenibacterium	insulin	-0.0007517	-0.0041856	0.00268211	-0.0705144	-0.3926126	0.25158392	0.66034868	Infected
Catenibacterium	glucose	4.52210991388741e-05	-0.0002531	0.00034349	0.04868253	-0.2724209	0.36978595	0.76073321	Infected
Desulfovibrio	BP_Sys	7.2652679908335e-06	-0.0054527	0.00546723	0.000388	-0.2911978	0.29197379	0.99786623	Infected
Desulfovibrio	BP_Dia	-0.0002813	-0.0043788	0.0038162	-0.0205232	-0.3194796	0.27843323	0.89027799	Infected
Desulfovibrio	Chol_T	-0.0003683	-0.0008563	0.00011968	-0.2369558	-0.5509053	0.0769936	0.13492012	Infected
Desulfovibrio	Chol_L	-0.0002091	-0.0006183	0.00020017	-0.161285	-0.4769799	0.15440992	0.30779644	Infected
Desulfovibrio	insulin	-0.0002701	-0.0033204	0.00278028	-0.0296921	-0.3650404	0.30565629	0.85879343	Infected
Desulfovibrio	glucose	6.11994807807504e-05	-0.0002028	0.00032518	0.07721771	-0.255859	0.41029443	0.641735	Infected
CAG.352	BP_Sys	0.0038535	-0.0072726	0.01497959	0.09705841	-0.1831754	0.37729222	0.48773954	Infected
CAG.352	BP_Dia	0.00022026	-0.0081836	0.0086241	0.00757912	-0.2816015	0.29675978	0.95799249	Infected
CAG.352	Chol_T	0.0001992	-0.0008289	0.00122729	0.06044165	-0.2515045	0.37238778	0.69725823	Infected
CAG.352	Chol_L	0.00054789	-0.000284	0.00137979	0.19932575	-0.103324	0.50197552	0.19054403	Infected
CAG.352	insulin	-0.0004846	-0.0067401	0.00577086	-0.0251285	-0.3494738	0.29921688	0.87628436	Infected
CAG.352	glucose	0.00011942	-0.000422	0.00066087	0.07106472	-0.2511382	0.39326767	0.65797594	Infected
Eubacterium_eligens_group	BP_Sys	0.0026706	-0.0056543	0.01099549	0.08793478	-0.1861793	0.36204886	0.52022421	Infected
Eubacterium_eligens_group	BP_Dia	-0.0022694	-0.0085089	0.0039702	-0.1020863	-0.3827713	0.17859873	0.46633857	Infected
Eubacterium_eligens_group	Chol_T	-0.0001107	-0.00088	0.00065854	-0.0439153	-0.349048	0.26121741	0.77251014	Infected
Eubacterium_eligens_group	Chol_L	7.78656530804049e-05	-0.0005575	0.00071326	0.0370331	-0.2651625	0.3392287	0.80553218	Infected
Eubacterium_eligens_group	insulin	0.00149642	-0.0031563	0.00614915	0.10143165	-0.2139437	0.41680695	0.51915655	Infected
Eubacterium_eligens_group	glucose	0.00010644	-0.0002979	0.00051077	0.08280153	-0.231748	0.39735104	0.5974351	Infected
Asteroleplasma	BP_Sys	0.00067876	-0.0018748	0.00323234	0.07481285	-0.2066417	0.35626742	0.59387552	Infected

Asteroleplasma	BP_Dia	0.00035909	-0.0015613	0.00227949	0.05407164	-0.2351066	0.34324993	0.70732525	Infected
Asteroleplasma	Chol_T	-2.15E-05	-0.0002572	0.00021421	-0.0285526	-0.3415346	0.28442944	0.85455769	Infected
Asteroleplasma	Chol_L	8.51099459611226e-06	-0.0001862	0.00020322	0.01354973	-0.2964314	0.32353086	0.92999948	Infected
Asteroleplasma	insulin	0.00025518	-0.0011749	0.00168529	0.05789861	-0.2665875	0.38238471	0.72011278	Infected
Asteroleplasma	glucose	1.691671796127e-05	-0.0001072	0.00014106	0.04405219	-0.2792347	0.36733906	0.78429595	Infected
Enterobacter	BP_Sys	-0.0034085	-0.0089685	0.00215142	-0.1704995	-0.4486159	0.107617	0.22238021	Infected
Enterobacter	BP_Dia	-0.0021523	-0.0063499	0.00204523	-0.1470871	-0.4339434	0.13976911	0.30605586	Infected
Enterobacter	Chol_T	8.76970434069238e-05	-0.0004331	0.00060847	0.05284588	-0.2609705	0.36666225	0.73522071	Infected
Enterobacter	Chol_L	-1.18E-05	-0.0004425	0.00041884	-0.0085319	-0.3196844	0.30262068	0.95605305	Infected
Enterobacter	insulin	0.00128017	-0.0018608	0.00442109	0.13182301	-0.191607	0.455253	0.41472337	Infected
Enterobacter	glucose	6.9662646679815e-05	-0.0002042	0.00034357	0.08232811	-0.2413802	0.40603643	0.60985647	Infected
Megamonas	BP_Sys	0.00080904	-0.0020223	0.00364037	0.08083608	-0.2020575	0.36372964	0.56660117	Infected
Megamonas	BP_Dia	0.00139099	-0.0006953	0.00347731	0.18987589	-0.0949156	0.47466741	0.18525592	Infected
Megamonas	Chol_T	0.00027693	3.11689916030601e-05	0.00052269	0.33333081	0.03751684	0.62914479	0.02820738	Infected
Megamonas	Chol_L	0.00023294	3.05084938845035e-05	0.00043537	0.33617416	0.04402964	0.62831867	0.02521846	Infected
Megamonas	insulin	0.00255811	0.00120197	0.00391425	0.52616173	0.24722502	0.80509845	0.00047352	Infected
Megamonas	glucose	-3.25E-05	-0.00017	0.00010495	-0.0767495	-0.401241	0.24774193	0.63502815	Infected
Methanosphaera	BP_Sys	0.00503739	3.61976026784939e-06	0.01007115	0.27081394	0.0001946	0.54143328	0.0498434	Infected
Methanosphaera	BP_Dia	-0.0009347	-0.0048953	0.00302584	-0.0686521	-0.359546	0.22224182	0.63576824	Infected
Methanosphaera	Chol_T	-0.0003341	-0.0008088	0.00014061	-0.2163642	-0.5237957	0.09106725	0.16253403	Infected
Methanosphaera	Chol_L	-0.000105	-0.0005056	0.00029562	-0.0815168	-0.3925875	0.22955391	0.59908176	Infected
Methanosphaera	insulin	-0.0003345	-0.00329	0.00262099	-0.0370225	-0.3641127	0.29006769	0.82010938	Infected
Methanosphaera	glucose	6.14800999895237e-05	-0.0001943	0.00031727	0.07808958	-0.2468014	0.40298052	0.62956983	Infected
Romboutsia	BP_Sys	0.00029408	-0.0004441	0.00103222	0.13121708	-0.1981404	0.4605746	0.42342717	UnInfected
Romboutsia	BP_Dia	0.00033795	-0.0002052	0.00088109	0.21904351	-0.1329932	0.57108021	0.21440903	UnInfected
Romboutsia	Chol_T	-4.17E-05	-9.33E-05	9.94247800906712e-06	-0.2471808	-0.5533232	0.05896154	0.10994618	UnInfected

Romboutsia	Chol_L	-2.69E-05	-7.30E-05	1.91785555510464e-05	-0.1808231	-0.4904581	0.12881201	0.24326241	UnInfected
Romboutsia	insulin	3.95078273086109e-06	-0.0003087	0.00031663	0.00458001	-0.3578969	0.36705691	0.97964607	UnInfected
Romboutsia	glucose	2.58089696149119e-06	-3.19E-05	3.70212962814196e-05	0.02808225	-0.3466572	0.40282169	0.87975043	UnInfected
Clostridium_sensu_stricto_1	BP_Sys	2.25447622562552e-05	-0.0004188	0.00046393	0.01617919	-0.3005826	0.33294101	0.91786451	UnInfected
Clostridium_sensu_stricto_1	BP_Dia	0.00011691	-0.0002098	0.00044366	0.12187168	-0.2187572	0.4625006	0.47179771	UnInfected
Clostridium_sensu_stricto_1	Chol_T	-2.82E-05	-5.84E-05	1.96853569773626e-06	-0.2691376	-0.5570511	0.01877591	0.06595202	UnInfected
Clostridium_sensu_stricto_1	Chol_L	-1.51E-05	-4.25E-05	1.22399753620176e-05	-0.163333	-0.4588882	0.13222222	0.26898256	UnInfected
Clostridium_sensu_stricto_1	insulin	2.12538904341579e-05	-0.0001638	0.00020627	0.03962829	-0.3053415	0.38459809	0.81665065	UnInfected
Clostridium_sensu_stricto_1	glucose	1.09998673348188e-05	-9.03E-06	3.10274448203186e-05	0.19250061	-0.1579873	0.54298857	0.27188047	UnInfected
Succinivibrio	BP_Sys	0.00025693	-0.0001309	0.00064476	0.19639098	-0.1000665	0.49284843	0.18690843	UnInfected
Succinivibrio	BP_Dia	-0.0001105	-0.0004052	0.0001841	-0.1227271	-0.449868	0.20441372	0.45073662	UnInfected
Succinivibrio	Chol_T	-1.60E-07	-2.89E-05	2.85329001346761e-05	-0.0016291	-0.2931299	0.28987162	0.99099631	UnInfected
Succinivibrio	Chol_L	9.7021823169723e-06	-1.52E-05	3.46240397825267e-05	0.11163374	-0.1751183	0.39838574	0.43399124	UnInfected
Succinivibrio	insulin	9.42405954285783e-05	-6.95E-05	0.00025797	0.18715741	-0.1379996	0.51231438	0.24996599	UnInfected
Succinivibrio	glucose	-2.07E-07	-1.86E-05	1.82041964482388e-05	-0.0038583	-0.3470436	0.33932693	0.98188906	UnInfected
Prevotella	BP_Sys	7.60023474197086e-06	-0.0003234	0.0003386	0.00739088	-0.3144927	0.32927446	0.96302177	UnInfected
Prevotella	BP_Dia	-7.59E-05	-0.0003214	0.00016962	-0.1071848	-0.4539722	0.23960251	0.53379128	UnInfected
Prevotella	Chol_T	9.36387150878037e-06	-1.42E-05	3.29739514305462e-05	0.12102388	-0.1841259	0.42617368	0.42550249	UnInfected
Prevotella	Chol_L	3.59968026278635e-06	-1.73E-05	2.44647460404483e-05	0.05269214	-0.2527308	0.35811507	0.72782056	UnInfected
Prevotella	insulin	8.46231598050317e-05	-5.09E-05	0.00022019	0.21380314	-0.1287187	0.556325	0.21298441	UnInfected
Prevotella	glucose	-4.15E-06	-1.94E-05	1.1080825826223e-05	-0.0983382	-0.4594457	0.2627694	0.58328211	UnInfected
Intestinibacter	BP_Sys	0.00015161	-0.0006608	0.00096405	0.05609687	-0.2445206	0.35671435	0.70663631	UnInfected
Intestinibacter	BP_Dia	0.00020789	-0.0003951	0.00081089	0.11173992	-0.2123635	0.43584331	0.48795073	UnInfected
Intestinibacter	Chol_T	-3.95E-05	-9.65E-05	1.74307231058089e-05	-0.1943508	-0.4744218	0.08572005	0.16736327	UnInfected
Intestinibacter	Chol_L	-5.99E-05	-0.0001068	-1.31E-05	-0.3338292	-0.5946759	-0.0729825	0.01371368	UnInfected

Intestinibacter	insulin	0.00028514	-4.11E-05	0.00061139	0.27411361	-0.0395249	0.5877521	0.08459949	UnInfected
Intestinibacter	glucose	4.07432310287656e-05	5.98924371160837e-06	7.54972183459228e-05	0.36762881	0.05404133	0.68121628	0.02297254	UnInfected
Bifidobacterium	BP_Sys	1.20132113803676e-05	-0.0003724	0.00039647	0.00955376	-0.2961922	0.3152997	0.94969356	UnInfected
Bifidobacterium	BP_Dia	-2.15E-05	-0.0003083	0.00026522	-0.0248708	-0.3561319	0.30639025	0.87952576	UnInfected
Bifidobacterium	Chol_T	1.97803306582847e-05	-7.01E-06	4.65724879478594e-05	0.20907157	-0.0741127	0.49225582	0.14259151	UnInfected
Bifidobacterium	Chol_L	1.35286659110485e-05	-1.03E-05	3.73317013676659e-05	0.16195064	-0.1229937	0.44689498	0.25584712	UnInfected
Bifidobacterium	insulin	-4.60E-05	-0.0002065	0.00011443	-0.0950727	-0.4265814	0.23643596	0.56354068	UnInfected
Bifidobacterium	glucose	-8.23E-06	-2.58E-05	9.30120477224136e-06	-0.15956	-0.4994997	0.1803797	0.34654375	UnInfected
Blautia	BP_Sys	0.00023508	-0.0008773	0.00134744	0.06300611	-0.2351313	0.36114357	0.6700202	UnInfected
Blautia	BP_Dia	-0.0001664	-0.0009966	0.00066373	-0.0647933	-0.3879955	0.25840886	0.68600666	UnInfected
Blautia	Chol_T	7.95885356524348e-05	4.35113330787066e-06	0.00015483	0.2835097	0.01549958	0.55151982	0.03879355	UnInfected
Blautia	Chol_L	6.74011282833175e-05	1.12503169613443e-06	0.00013368	0.27192606	0.00453888	0.53931325	0.04644746	UnInfected
Blautia	insulin	-0.0001934	-0.0006562	0.00026947	-0.1346544	-0.4569565	0.18764761	0.40145054	UnInfected
Blautia	glucose	-1.04E-05	-6.18E-05	4.1057189402662e-05	-0.0677481	-0.4038412	0.26834509	0.68437716	UnInfected
Faecalibacterium	BP_Sys	-0.0001391	-0.0014477	0.00116943	-0.0324665	-0.3378317	0.27289869	0.8300776	UnInfected
Faecalibacterium	BP_Dia	-0.0003595	-0.0013281	0.00060918	-0.1218551	-0.4502109	0.20650068	0.45559043	UnInfected
Faecalibacterium	Chol_T	0.0001149	2.98092839252749e-05	0.00019999	0.35635715	0.09245403	0.62026027	0.00966034	UnInfected
Faecalibacterium	Chol_L	3.12130292544793e-05	-5.07E-05	0.00011317	0.10964189	-0.1782368	0.39752053	0.44393442	UnInfected
Faecalibacterium	insulin	0.00024907	-0.0002931	0.0007912	0.15100741	-0.1776849	0.47969968	0.35673534	UnInfected
Faecalibacterium	glucose	5.62445045030829e-06	-5.49E-05	6.61117844863797e-05	0.03200674	-0.3122051	0.37621857	0.85111002	UnInfected
Dialister	BP_Sys	-3.81E-05	-0.0012025	0.00112638	-0.0100191	-0.3164694	0.29643124	0.94736811	UnInfected
Dialister	BP_Dia	-0.0004214	-0.0012773	0.00043453	-0.161092	-0.4882988	0.16611471	0.32380473	UnInfected
Dialister	Chol_T	2.03188717629219e-05	-6.32E-05	0.00010389	0.07106953	-0.2212295	0.36336857	0.62410935	UnInfected
Dialister	Chol_L	4.01580693153229e-05	-3.20E-05	0.00011231	0.15908269	-0.1267485	0.44491385	0.26565517	UnInfected
Dialister	insulin	0.00040913	-5.74E-05	0.0008756	0.27973646	-0.0392148	0.59868768	0.08356033	UnInfected
Dialister	glucose	2.46408231112286e-05	-2.85E-05	7.77496573584905e-05	0.1581343	-0.1826956	0.49896416	0.35205498	UnInfected

Subdoligranulum	BP_Sys	0.00035606	-0.001511	0.00222309	0.05647027	-0.2396387	0.35257928	0.70050939	UnInfected
Subdoligranulum	BP_Dia	2.17497620929325e-05	-0.0013744	0.00141787	0.00501075	-0.3166292	0.32665067	0.97490602	UnInfected
Subdoligranulum	Chol_T	2.73948516300099e-05	-0.000107	0.00016183	0.05774518	-0.2256251	0.34111547	0.68112243	UnInfected
Subdoligranulum	Chol_L	-1.24E-05	-0.0001305	0.00010566	-0.0296852	-0.3116117	0.25224125	0.83169167	UnInfected
Subdoligranulum	insulin	-0.0003067	-0.0010841	0.00047063	-0.1263902	-0.4467047	0.19392419	0.42784452	UnInfected
Subdoligranulum	glucose	-4.08E-06	-9.06E-05	8.2388966527308e-05	-0.0157943	-0.3502304	0.31864189	0.92403575	UnInfected
Terrisporobacter	BP_Sys	6.03949165184771e-05	-0.0005803	0.00070105	0.02880205	-0.2767236	0.33432766	0.8490783	UnInfected
Terrisporobacter	BP_Dia	0.00030878	-0.0001568	0.00077436	0.21390609	-0.1086196	0.53643179	0.18641484	UnInfected
Terrisporobacter	Chol_T	-5.52E-05	-9.70E-05	-1.34E-05	-0.3499056	-0.6150101	-0.0848012	0.01125008	UnInfected
Terrisporobacter	Chol_L	-4.21E-05	-7.97E-05	-4.46E-06	-0.3022087	-0.5723741	-0.0320432	0.02947698	UnInfected
Terrisporobacter	insulin	-0.0001784	-0.0004398	8.29144085432141e-05	-0.221075	-0.5448827	0.10273267	0.17412512	UnInfected
Terrisporobacter	glucose	2.45114948816654e-06	-2.72E-05	3.20640253190737e-05	0.02850534	-0.3158739	0.3728846	0.86729407	UnInfected
Collinsella	BP_Sys	0.00065143	-0.0002535	0.00155635	0.20895976	-0.0813083	0.49922784	0.15248782	UnInfected
Collinsella	BP_Dia	0.00045157	-0.0002266	0.00112971	0.21041087	-0.1055676	0.52638939	0.1846879	UnInfected
Collinsella	Chol_T	-1.88E-06	-6.91E-05	6.53791600004293e-05	-0.0080189	-0.2947639	0.27872613	0.95497133	UnInfected
Collinsella	Chol_L	1.46537678636604e-05	-4.41E-05	7.33999443561072e-05	0.07075448	-0.2128965	0.35440541	0.61518359	UnInfected
Collinsella	insulin	0.00011128	-0.0002784	0.000501	0.09273929	-0.23205	0.41752857	0.56523505	UnInfected
Collinsella	glucose	-3.50E-05	-7.63E-05	6.3434465718413e-06	-0.2737634	-0.5971461	0.0496193	0.09436903	UnInfected
Escherichia.Shigella	BP_Sys	-0.0006856	-0.0011551	-0.0002162	-0.3956186	-0.6664978	-0.1247395	0.00549576	UnInfected
Escherichia.Shigella	BP_Dia	-0.0003723	-0.0007439	-7.09E-07	-0.3120836	-0.623573	-0.0005942	0.04958922	UnInfected
Escherichia.Shigella	Chol_T	-4.27E-06	-4.23E-05	3.37701511139188e-05	-0.0327286	-0.3244435	0.25898628	0.82085269	UnInfected
Escherichia.Shigella	Chol_L	-7.36E-07	-3.41E-05	3.26395465583925e-05	-0.0063959	-0.2962924	0.2835007	0.96446821	UnInfected
Escherichia.Shigella	insulin	-7.71E-05	-0.0002971	0.00014294	-0.1155348	-0.4453558	0.21428621	0.48105218	UnInfected
Escherichia.Shigella	glucose	1.9448373756681e-05	-3.99E-06	4.28836537086969e-05	0.27366173	-0.0561005	0.60342398	0.10076526	UnInfected
Sarcina	BP_Sys	-0.0002392	-0.001185	0.00070657	-0.0874951	-0.4334123	0.25842201	0.61025969	UnInfected
Sarcina	BP_Dia	-0.0001222	-0.0008293	0.00058497	-0.0649129	-0.4406055	0.31077969	0.72742723	UnInfected

Sarcina	Chol_T	-6.51E-05	-0.0001295	-6.55E-07	-0.3162228	-0.6292601	-0.0031856	0.04784271	UnInfected
Sarcina	Chol_L	-2.99E-05	-8.89E-05	2.91440048685605e-05	-0.1645147	-0.4894781	0.16044863	0.31050688	UnInfected
Sarcina	insulin	-0.0002549	-0.0006428	0.00013308	-0.2421774	-0.6108117	0.12645695	0.19049872	UnInfected
Sarcina	glucose	2.20428633301114e-05	-2.11E-05	6.52297491087637e-05	0.1965964	-0.1885798	0.58177259	0.30661934	UnInfected
Holdemanella	BP_Sys	0.00069064	-0.0006185	0.00199982	0.16369982	-0.1466127	0.47401233	0.29093489	UnInfected
Holdemanella	BP_Dia	0.00036203	-0.0006233	0.00134738	0.12464925	-0.2146168	0.46391535	0.46005652	UnInfected
Holdemanella	Chol_T	9.77686417951162e-06	-8.61E-05	0.00010564	0.03079963	-0.2712019	0.33280119	0.83690229	UnInfected
Holdemanella	Chol_L	3.0604708559607e-05	-5.28E-05	0.00011401	0.10919443	-0.1883904	0.40677928	0.46062964	UnInfected
Holdemanella	insulin	-0.000435	-0.000972	0.00010199	-0.2678763	-0.5985581	0.06280538	0.10882108	UnInfected
Holdemanella	glucose	-3.87E-05	-9.87E-05	2.12999158108235e-05	-0.223731	-0.5705771	0.12311497	0.19845637	UnInfected
Bacteroides	BP_Sys	7.17193010288671e-05	-0.0005048	0.00064823	0.03705875	-0.2608338	0.33495135	0.80176108	UnInfected
Bacteroides	BP_Dia	-9.80E-05	-0.0005271	0.00033117	-0.0735343	-0.395643	0.24857449	0.64536816	UnInfected
Bacteroides	Chol_T	3.57070859855734e-05	-3.89E-06	7.53010148992054e-05	0.24521981	-0.026693	0.51713267	0.07556513	UnInfected
Bacteroides	Chol_L	2.71040712851824e-05	-8.05E-06	6.22598299112647e-05	0.21081521	-0.0626259	0.48425636	0.1262925	UnInfected
Bacteroides	insulin	-9.47E-05	-0.0003344	0.00014503	-0.1271221	-0.4489386	0.19469434	0.42734313	UnInfected
Bacteroides	glucose	-5.31E-06	-3.19E-05	2.12904933398718e-05	-0.0669498	-0.4021707	0.26827111	0.68712312	UnInfected
Streptococcus	BP_Sys	-0.0006973	-0.0013508	-4.39E-05	-0.3048072	-0.5904401	-0.0191743	0.03721677	UnInfected
Streptococcus	BP_Dia	-6.58E-05	-0.0005865	0.00045489	-0.0417748	-0.3723746	0.28882494	0.79871101	UnInfected
Streptococcus	Chol_T	2.12352976152269e-07	-5.01E-05	5.05293138020476e-05	0.00123363	-0.2910755	0.29354279	0.99320092	UnInfected
Streptococcus	Chol_L	-2.71E-06	-4.68E-05	4.13978733878033e-05	-0.0178205	-0.3080191	0.27237818	0.90133285	UnInfected
Streptococcus	insulin	-0.0001292	-0.0004186	0.00016025	-0.1467012	-0.4753844	0.18198201	0.37042769	UnInfected
Streptococcus	glucose	5.1172059533126e-06	-2.71E-05	3.73526803366979e-05	0.05454412	-0.2890527	0.39814093	0.74875692	UnInfected
Clostridia_UCG.014	BP_Sys	0.00038568	-0.0006614	0.00143277	0.10856914	-0.186189	0.40332727	0.45893779	UnInfected
Clostridia_UCG.014	BP_Dia	0.00046247	-0.0003081	0.0012331	0.18911121	-0.1260059	0.50422834	0.23074826	UnInfected
Clostridia_UCG.014	Chol_T	3.52643391008379e-05	-3.98E-05	0.00011029	0.13193602	-0.1487604	0.41263238	0.34588163	UnInfected
Clostridia_UCG.014	Chol_L	4.25534177901751e-05	-2.24E-05	0.00010751	0.18031353	-0.0949467	0.45557379	0.1917462	UnInfected

Clostridia_UCG.014	insulin	7.60528888001193e-05	-0.0003661	0.00051816	0.05562276	-0.267719	0.3789645	0.72857254	UnInfected
Clostridia_UCG.014	glucose	-7.40E-06	-5.61E-05	4.13319512096736e-05	-0.0508043	-0.3853355	0.28372693	0.75928219	UnInfected
Agathobacter	BP_Sys	0.00056295	-0.0010963	0.00222219	0.10004801	-0.1948372	0.39493318	0.49485335	UnInfected
Agathobacter	BP_Dia	-0.0001265	-0.0013726	0.00111952	-0.0326613	-0.3543396	0.28901694	0.83761276	UnInfected
Agathobacter	Chol_T	7.31412687825511e-05	-4.44E-05	0.00019069	0.17276293	-0.1048959	0.45042172	0.21441242	UnInfected
Agathobacter	Chol_L	4.79195078540215e-05	-5.62E-05	0.00015208	0.1281937	-0.1504661	0.40685348	0.35609738	UnInfected
Agathobacter	insulin	-0.0004155	-0.0011009	0.00026985	-0.1918562	-0.5083133	0.12460093	0.22612123	UnInfected
Agathobacter	glucose	-4.93E-05	-0.0001245	2.59543772879298e-05	-0.2135916	-0.5396658	0.1124827	0.19176338	UnInfected
Pseudomonas	BP_Sys	0.00245111	-0.0018339	0.00673614	0.17467587	-0.1306932	0.48004489	0.25286093	UnInfected
Pseudomonas	BP_Dia	0.0018232	-0.0013742	0.0050206	0.18873531	-0.1422551	0.51972572	0.25432913	UnInfected
Pseudomonas	Chol_T	6.44618774065281e-05	-0.0002496	0.00037854	0.06105484	-0.2364247	0.35853442	0.67896975	UnInfected
Pseudomonas	Chol_L	0.00010536	-0.0001682	0.00037893	0.11301687	-0.1804467	0.4064804	0.43890937	UnInfected
Pseudomonas	insulin	-0.0011769	-0.0029627	0.00060903	-0.2178956	-0.5485531	0.11276203	0.18917543	UnInfected
Pseudomonas	glucose	-0.0001236	-0.0003209	7.36526711667102e-05	-0.2148147	-0.5576242	0.1279949	0.21125247	UnInfected
Turicibacter	BP_Sys	-8.58E-05	-0.0024565	0.0022848	-0.0126749	-0.3627085	0.33735871	0.94171714	UnInfected
Turicibacter	BP_Dia	0.00049585	-0.0012643	0.00225596	0.10635028	-0.271162	0.48386257	0.57042827	UnInfected
Turicibacter	Chol_T	-0.0001409	-0.0003042	2.24169227186304e-05	-0.2764927	-0.5969766	0.0439912	0.08849758	UnInfected
Turicibacter	Chol_L	-0.0001099	-0.0002545	3.47018185768804e-05	-0.2442058	-0.565539	0.07712733	0.13159725	UnInfected
Turicibacter	insulin	-0.0002295	-0.0012206	0.00076161	-0.0880432	-0.4682555	0.29216913	0.64065553	UnInfected
Turicibacter	glucose	2.10823589681719e-05	-8.82E-05	0.0001304	0.07590948	-0.3177029	0.46952185	0.69731271	UnInfected
Roseburia	BP_Sys	0.0017932	-0.0026447	0.0062311	0.12296555	-0.1813567	0.42728777	0.41693537	UnInfected
Roseburia	BP_Dia	3.58854717091383e-05	-0.0033089	0.00338065	0.00357456	-0.3295979	0.33674705	0.9827166	UnInfected
Roseburia	Chol_T	0.00011076	-0.0002097	0.00043128	0.10094911	-0.1911629	0.39306111	0.48692751	UnInfected
Roseburia	Chol_L	-7.12E-05	-0.0003532	0.0002108	-0.0734795	-0.3645543	0.21759517	0.6109553	UnInfected
Roseburia	insulin	0.00134772	-0.0004711	0.00316658	0.24011059	-0.0839381	0.56415926	0.14119288	UnInfected
Roseburia	glucose	-4.31E-05	-0.0002497	0.00016354	-0.0720646	-0.4175965	0.27346727	0.67408504	UnInfected

uncultured	BP_Sys	0.00302008	-0.0026397	0.00867988	0.15976512	-0.1396434	0.45917365	0.28551327	UnInfected
uncultured	BP_Dia	-0.0002505	-0.004547	0.00404604	-0.0192477	-0.3494106	0.31091513	0.90630539	UnInfected
uncultured	Chol_T	-0.0003029	-0.0007036	9.79121028201465e-05	-0.2129353	-0.4947115	0.06884081	0.13371433	UnInfected
uncultured	Chol_L	-0.00012	-0.0004813	0.00024125	-0.0955618	-0.3832333	0.19210966	0.5038468	UnInfected
uncultured	insulin	0.00103813	-0.0013498	0.00342601	0.14268207	-0.1855134	0.47087758	0.38282578	UnInfected
uncultured	glucose	-2.81E-05	-0.0002941	0.00023793	-0.0362388	-0.379413	0.30693541	0.83121239	UnInfected
Lactobacillus	BP_Sys	-0.0001627	-0.0019138	0.00158835	-0.0289662	-0.3407009	0.28276853	0.85121479	UnInfected
Lactobacillus	BP_Dia	-0.0007783	-0.002056	0.00049945	-0.2012696	-0.5316995	0.12916027	0.22399358	UnInfected
Lactobacillus	Chol_T	3.15932683652194e-05	-9.41E-05	0.00015729	0.07475272	-0.2226526	0.37215802	0.61249356	UnInfected
Lactobacillus	Chol_L	1.72661365363513e-05	-9.32E-05	0.00012774	0.0462694	-0.2497727	0.34231154	0.75250284	UnInfected
Lactobacillus	insulin	-1.20E-05	-0.0007469	0.00072285	-0.0055586	-0.3454571	0.33433996	0.97365838	UnInfected
Lactobacillus	glucose	-3.03E-05	-0.0001106	4.9924703101249e-05	-0.1316782	-0.4800943	0.21673794	0.44741418	UnInfected
Lachnospiraceae_NK4A136_group	BP_Sys	0.00050879	-0.0035292	0.00454677	0.03800772	-0.2636387	0.33965417	0.79927144	UnInfected
Lachnospiraceae_NK4A136_group	BP_Dia	0.00030663	-0.0027071	0.00332035	0.0332733	-0.2937548	0.36030135	0.83728135	UnInfected
Lachnospiraceae_NK4A136_group	Chol_T	7.31066842266437e-05	-0.0002169	0.00036309	0.0725835	-0.2153209	0.36048794	0.61142327	UnInfected
Lachnospiraceae_NK4A136_group	Chol_L	4.47369671536396e-05	-0.00021	0.0002995	0.05030528	-0.2361716	0.33678218	0.72317151	UnInfected
Lachnospiraceae_NK4A136_group	insulin	0.00010242	-0.0015926	0.00179745	0.01987759	-0.3091006	0.34885581	0.90290823	UnInfected
Lachnospiraceae_NK4A136_group	glucose	5.98854339558966e-05	-0.0001257	0.00024549	0.10909103	-0.2290103	0.44719239	0.51608504	UnInfected
Comamonas	BP_Sys	0.00030924	-0.0010797	0.00169816	0.06593607	-0.2302076	0.36207971	0.6535256	UnInfected
Comamonas	BP_Dia	-0.0005464	-0.0015677	0.00047494	-0.1692202	-0.4855403	0.14709982	0.28430598	UnInfected
Comamonas	Chol_T	-0.0001034	-0.0001968	-9.96E-06	-0.2929911	-0.5577578	-0.0282243	0.03113996	UnInfected
Comamonas	Chol_L	-7.17E-05	-0.0001559	1.25829631524792e-05	-0.2299991	-0.5003834	0.04038509	0.09285916	UnInfected
Comamonas	insulin	-1.46E-05	-0.0005989	0.00056981	-0.0080627	-0.3317814	0.31565613	0.9598921	UnInfected
Comamonas	glucose	-3.94E-05	-0.0001023	2.34555131613555e-05	-0.2048835	-0.5317232	0.12195618	0.21108698	UnInfected
Klebsiella	BP_Sys	-0.0002606	-0.0013561	0.00083485	-0.0851746	-0.443192	0.27284284	0.63156571	UnInfected
Klebsiella	BP_Dia	8.68523379355206e-05	-0.0007328	0.00090649	0.04123169	-0.3478773	0.43034069	0.83063733	UnInfected

Klebsiella	Chol_T	-7.36E-05	-0.0001484	1.14359505510565e-06	-0.3197901	-0.6445476	0.0049673	0.05340284	UnInfected
Klebsiella	Chol_L	-3.53E-05	-0.0001036	3.29564598651801e-05	-0.1738167	-0.5097602	0.16212678	0.30014605	UnInfected
Klebsiella	insulin	-0.0004472	-0.0008802	-1.41E-05	-0.379674	-0.7474003	-0.0119477	0.04339661	UnInfected
Klebsiella	glucose	8.61735837711273e-06	-4.21E-05	5.93344583345072e-05	0.06867665	-0.3355169	0.4728702	0.73177344	UnInfected
Senegalimassilia	BP_Sys	-0.0003031	-0.0053179	0.00471181	-0.0194405	-0.341132	0.30225101	0.90289239	UnInfected
Senegalimassilia	BP_Dia	0.00061686	-0.0031191	0.00435285	0.05747922	-0.2906451	0.40560353	0.73905683	UnInfected
Senegalimassilia	Chol_T	-5.54E-05	-0.0004162	0.00030532	-0.0472649	-0.3548315	0.26030179	0.75651297	UnInfected
Senegalimassilia	Chol_L	5.84478709977807e-05	-0.0002576	0.00037454	0.05643661	-0.2487795	0.3616527	0.70917885	UnInfected
Senegalimassilia	insulin	-0.0009787	-0.0030539	0.00109653	-0.1631105	-0.5089705	0.18274959	0.34428749	UnInfected
Senegalimassilia	glucose	-8.66E-05	-0.0003164	0.00014315	-0.1355235	-0.4949709	0.22392379	0.44848774	UnInfected
Christensenellaceae_R.7_group	BP_Sys	0.00208928	-0.0037509	0.00792944	0.10832513	-0.1944759	0.41112617	0.47184775	UnInfected
Christensenellaceae_R.7_group	BP_Dia	0.00036055	-0.0040297	0.00475078	0.02715463	-0.3034953	0.35780459	0.86832316	UnInfected
Christensenellaceae_R.7_group	Chol_T	7.98839416479345e-05	-0.0003432	0.00050295	0.05504762	-0.2364863	0.34658152	0.7033253	UnInfected
Christensenellaceae_R.7_group	Chol_L	3.92274467645637e-05	-0.0003323	0.00041073	0.03061508	-0.259326	0.32055612	0.83122515	UnInfected
Christensenellaceae_R.7_group	insulin	1.59438930945692e-05	-0.0024533	0.00248518	0.00214774	-0.3304741	0.33476956	0.98959767	UnInfected
Christensenellaceae_R.7_group	glucose	1.09494120444049e-06	-0.000271	0.00027317	0.00138439	-0.3426082	0.34537694	0.99351643	UnInfected
Gastranaerophilales	BP_Sys	0.00102835	-0.0021758	0.00423245	0.09719445	-0.2056418	0.40003069	0.51829794	UnInfected
Gastranaerophilales	BP_Dia	0.00259921	0.00037635	0.00482206	0.35685422	0.05167102	0.66203742	0.02330052	UnInfected
Gastranaerophilales	Chol_T	-2.37E-06	-0.0002346	0.00022989	-0.0029834	-0.2947497	0.2887829	0.98352766	UnInfected
Gastranaerophilales	Chol_L	-7.47E-05	-0.0002767	0.00012719	-0.1063232	-0.3935955	0.18094914	0.45678836	UnInfected
Gastranaerophilales	insulin	-0.0002013	-0.0015521	0.00114942	-0.0494361	-0.3811225	0.28225024	0.76361504	UnInfected
Gastranaerophilales	glucose	-6.73E-05	-0.0002145	7.97824746162181e-05	-0.1551929	-0.4942692	0.18388338	0.35852123	UnInfected
Coprococcus	BP_Sys	0.00074478	-0.0085652	0.01005475	0.02440738	-0.2806927	0.32950744	0.87170232	UnInfected
Coprococcus	BP_Dia	-0.000633	-0.0075782	0.00631225	-0.0301311	-0.3607494	0.30048733	0.85403733	UnInfected
Coprococcus	Chol_T	0.00073375	0.00011528	0.00135221	0.31958515	0.05021263	0.58895767	0.02149925	UnInfected
Coprococcus	Chol_L	0.00048957	-7.25E-05	0.00105161	0.24150457	-0.0357463	0.51875539	0.08559431	UnInfected

Coprococcus	insulin	-0.0011367	-0.0050225	0.00274917	-0.0967802	-0.4276329	0.23407256	0.55581531	UnInfected
Coprococcus	glucose	-0.0002174	-0.0006409	0.00020614	-0.1737113	-0.5121587	0.1647362	0.30396671	UnInfected
Dorea	BP_Sys	0.00383735	-0.004796	0.01247068	0.13473731	-0.1683969	0.43787152	0.37238956	UnInfected
Dorea	BP_Dia	0.00011365	-0.0064067	0.00663404	0.00579653	-0.3267701	0.33836321	0.97192582	UnInfected
Dorea	Chol_T	0.00047294	-0.0001339	0.00107974	0.22070247	-0.0624676	0.50387255	0.12234491	UnInfected
Dorea	Chol_L	0.00023361	-0.0003121	0.0007793	0.1234705	-0.1649411	0.41188209	0.39005388	UnInfected
Dorea	insulin	-0.0007265	-0.0043833	0.00293031	-0.0662746	-0.3998657	0.26731641	0.68867503	UnInfected
Dorea	glucose	-0.0002239	-0.00062	0.00017213	-0.1917301	-0.5308458	0.14738555	0.25829637	UnInfected
Treponema	BP_Sys	0.00687949	-0.0275894	0.04134836	0.06064958	-0.2432278	0.36452692	0.68731954	UnInfected
Treponema	BP_Dia	0.01138264	-0.0140811	0.03684639	0.14576807	-0.1803251	0.47186124	0.36970435	UnInfected
Treponema	Chol_T	-0.0023486	-0.0046944	-2.78E-06	-0.2751823	-0.5500389	-0.0003258	0.0497444	UnInfected
Treponema	Chol_L	-0.0013615	-0.0034897	0.00076679	-0.180673	-0.4631015	0.10175556	0.20209916	UnInfected
Treponema	insulin	-0.0082698	-0.0224648	0.00592529	-0.1894174	-0.5145526	0.13571778	0.24437653	UnInfected
Treponema	glucose	-0.0001054	-0.001702	0.00149117	-0.0226626	-0.365901	0.32057576	0.89395699	UnInfected
UCG.002	BP_Sys	0.00097519	-0.0019717	0.00392207	0.10055676	-0.2033101	0.40442359	0.50546405	UnInfected
UCG.002	BP_Dia	0.00060583	-0.0015974	0.00280908	0.0907449	-0.2392693	0.4207591	0.57964158	UnInfected
UCG.002	Chol_T	1.65140692515043e-05	-0.0001971	0.00023014	0.02263194	-0.2701374	0.31540122	0.8759872	UnInfected
UCG.002	Chol_L	-0.000157	-0.0003359	2.1922306341591e-05	-0.2437036	-0.5214339	0.03402672	0.08341506	UnInfected
UCG.002	insulin	0.00040674	-0.0008294	0.00164291	0.10896677	-0.2222049	0.44013842	0.50788141	UnInfected
UCG.002	glucose	0.00010711	-2.47E-05	0.00023889	0.26932664	-0.0620273	0.60068058	0.10767832	UnInfected
Ruminococcus_torques_group	BP_Sys	2.90222078102281e-05	-0.0040974	0.00415544	0.00216346	-0.3054401	0.30976705	0.98866938	UnInfected
Ruminococcus_torques_group	BP_Dia	0.00047464	-0.0025994	0.00354872	0.05139653	-0.2814783	0.38427136	0.75539724	UnInfected
Ruminococcus_torques_group	Chol_T	7.9795425660653e-05	-0.0002161	0.00037566	0.07905748	-0.2140744	0.37218933	0.58689979	UnInfected
Ruminococcus_torques_group	Chol_L	0.00010504	-0.0001529	0.00036297	0.11786983	-0.1715437	0.40728341	0.41328409	UnInfected
Ruminococcus_torques_group	insulin	-0.0014064	-0.003064	0.00025121	-0.2723861	-0.5934246	0.04865232	0.09366586	UnInfected
Ruminococcus_torques_group	glucose	-8.90E-05	-0.0002771	9.90708231940597e-05	-0.1618153	-0.503724	0.18009338	0.34261278	UnInfected

Ruminococcus	BP_Sys	0.00537566	-0.001809	0.01256029	0.21818724	-0.0734226	0.50979706	0.13747046	UnInfected
Ruminococcus	BP_Dia	0.00136967	-0.0041544	0.00689377	0.08075358	-0.2449386	0.40644572	0.61729924	UnInfected
Ruminococcus	Chol_T	0.00016057	-0.0003717	0.00069289	0.08661841	-0.200537	0.37377379	0.5436229	UnInfected
Ruminococcus	Chol_L	-0.000443	-0.0008853	-5.72E-07	-0.2706294	-0.5409095	-0.0003492	0.04972149	UnInfected
Ruminococcus	insulin	0.0014379	-0.0016378	0.00451363	0.15162846	-0.172713	0.4759699	0.34845376	UnInfected
Ruminococcus	glucose	8.76033926170287 e-05	-0.0002545	0.00042971	0.0867066	-0.2518989	0.42531211	0.60586094	UnInfected
Sutterella	BP_Sys	0.00517551	-0.0035477	0.01389873	0.18196285	-0.1247324	0.48865807	0.23598203	UnInfected
Sutterella	BP_Dia	-0.0044405	-0.0109023	0.00202124	-0.2267842	-0.556796	0.10322775	0.17140439	UnInfected
Sutterella	Chol_T	-4.42E-05	-0.0006861	0.00059759	-0.0206748	-0.3205925	0.27924295	0.88931587	UnInfected
Sutterella	Chol_L	0.00014867	-0.0004118	0.00070912	0.07868258	-0.2179224	0.37528751	0.59301996	UnInfected
Sutterella	insulin	0.00218326	-0.0014748	0.0058413	0.19943058	-0.1347133	0.53357448	0.23325881	UnInfected
Sutterella	glucose	-0.0002014	-0.0006072	0.00020432	-0.172692	-0.5205582	0.17517415	0.31984429	UnInfected
Ruminobacter	BP_Sys	0.00040826	-0.0026543	0.00347078	0.04121925	-0.2679822	0.35042068	0.7879123	UnInfected
Ruminobacter	BP_Dia	-0.0019777	-0.0041552	0.00019992	-0.2900419	-0.6094043	0.02932048	0.07362225	UnInfected
Ruminobacter	Chol_T	-0.0001195	-0.0003362	9.72561522522081 e-05	-0.1603286	-0.4511617	0.13050456	0.27013874	UnInfected
Ruminobacter	Chol_L	-5.49E-05	-0.0002475	0.00013778	-0.0833754	-0.3761442	0.20939346	0.56625804	UnInfected
Ruminobacter	insulin	0.00087246	-0.0003759	0.0021208	0.22885774	-0.098595	0.55631044	0.16443	UnInfected
Ruminobacter	glucose	-2.99E-05	-0.0001712	0.00011143	-0.0735536	-0.4214459	0.27433873	0.66988265	UnInfected
Eubacterium_hallii_ group	BP_Sys	0.00148757	-0.0060959	0.00907106	0.05892931	-0.241487	0.35934559	0.69239925	UnInfected
Eubacterium_hallii_ group	BP_Dia	-0.0022319	-0.0078481	0.00338432	-0.1284327	-0.4516139	0.19474853	0.4245824	UnInfected
Eubacterium_hallii_ group	Chol_T	0.00032328	-0.0002121	0.00085871	0.17021022	-0.1116944	0.45211487	0.22798113	UnInfected
Eubacterium_hallii_ group	Chol_L	8.75005164148568 e-05	-0.0003916	0.00056656	0.05217683	-0.23349	0.33784369	0.71256413	UnInfected
Eubacterium_hallii_ group	insulin	-0.0016996	-0.0048309	0.00143159	-0.1749312	-0.497205	0.14734265	0.27743282	UnInfected
Eubacterium_hallii_ group	glucose	-5.22E-05	-0.000403	0.00029865	-0.0504159	-0.3893389	0.28850717	0.76406327	UnInfected
Methanobrevibacte r	BP_Sys	-0.0011058	-0.0053076	0.00309597	-0.080817	-0.3878998	0.22626583	0.59593824	UnInfected
Methanobrevibacte r	BP_Dia	-0.000211	-0.0033586	0.00293661	-0.0223979	-0.356553	0.31175726	0.89235714	UnInfected

Methanobrevibacter	Chol_T	8.37711015659648e-05	-0.0002187	0.00038627	0.08136969	-0.2124613	0.37520066	0.57696308	UnInfected
Methanobrevibacter	Chol_L	0.00015699	-0.0001036	0.00041763	0.17270831	-0.114018	0.45943466	0.22906867	UnInfected
Methanobrevibacter	insulin	-0.0007033	-0.0024558	0.00104912	-0.1335501	-0.4663067	0.1992065	0.42004592	UnInfected
Methanobrevibacter	glucose	-7.54E-05	-0.0002686	0.00011781	-0.1343506	-0.4786689	0.20996771	0.43295234	UnInfected
Haemophilus	BP_Sys	0.00096249	-0.0032432	0.00516818	0.06772294	-0.2282006	0.36364644	0.64455692	UnInfected
Haemophilus	BP_Dia	0.00118068	-0.0019395	0.00430088	0.12067622	-0.198237	0.43958944	0.44686085	UnInfected
Haemophilus	Chol_T	0.00031282	2.98189426137349e-05	0.00059583	0.29254101	0.02788562	0.5571964	0.03131574	UnInfected
Haemophilus	Chol_L	0.0002399	-1.27E-05	0.00049246	0.25408821	-0.013408	0.52158444	0.06191415	UnInfected
Haemophilus	insulin	-0.0009506	-0.0026882	0.00078694	-0.1737807	-0.4914201	0.14385873	0.27371476	UnInfected
Haemophilus	glucose	-6.26E-05	-0.0002563	0.00013118	-0.1073571	-0.439797	0.22508279	0.51572438	UnInfected
Akkermansia	BP_Sys	0.000113	-0.0018565	0.00208247	0.01721844	-0.2828767	0.31731356	0.90777872	UnInfected
Akkermansia	BP_Dia	0.00035391	-0.0011104	0.00181825	0.07833463	-0.245781	0.40245025	0.62617474	UnInfected
Akkermansia	Chol_T	6.78516821925609e-05	-7.20E-05	0.00020769	0.13740957	-0.1457759	0.42059508	0.33072603	UnInfected
Akkermansia	Chol_L	3.39339624844573e-05	-8.99E-05	0.00015775	0.07783182	-0.2061627	0.36182639	0.58089335	UnInfected
Akkermansia	insulin	-0.0002014	-0.0010246	0.00062179	-0.0797266	-0.405609	0.24615567	0.62196717	UnInfected
Akkermansia	glucose	-4.79E-05	-0.0001373	4.15842756930093e-05	-0.1778531	-0.5102219	0.15451567	0.28417993	UnInfected
Catenibacterium	BP_Sys	0.0008887	-0.0094051	0.01118245	0.02597991	-0.2749449	0.32690469	0.8616447	UnInfected
Catenibacterium	BP_Dia	-0.0053372	-0.0127847	0.00211024	-0.2266459	-0.5429034	0.0896117	0.15428318	UnInfected
Catenibacterium	Chol_T	0.00030416	-0.0004298	0.00103807	0.11817757	-0.1669733	0.40332849	0.40519713	UnInfected
Catenibacterium	Chol_L	9.19713585350427e-05	-0.0005576	0.00074153	0.04047151	-0.245362	0.32630502	0.77509515	UnInfected
Catenibacterium	insulin	0.00395554	-0.0001307	0.00804181	0.30043188	-0.0099288	0.61079255	0.05734646	UnInfected
Catenibacterium	glucose	-0.0002812	-0.0007466	0.00018428	-0.2004336	-0.5322336	0.13136644	0.22776048	UnInfected
Desulfovibrio	BP_Sys	-0.0046105	-0.0108442	0.00162322	-0.2265372	-0.5328315	0.07975708	0.14190285	UnInfected
Desulfovibrio	BP_Dia	-0.0005209	-0.0053252	0.00428334	-0.03718	-0.3800787	0.30571865	0.82676339	UnInfected
Desulfovibrio	Chol_T	-9.12E-05	-0.0005542	0.00037179	-0.0595729	-0.3619417	0.24279587	0.69112032	UnInfected
Desulfovibrio	Chol_L	0.00010996	-0.0002951	0.00051504	0.08132833	-0.2182767	0.38093332	0.5844828	UnInfected

Desulfovibrio	insulin	-0.0003057	-0.0030064	0.00239513	-0.0390194	-0.3837954	0.30575653	0.81931681	UnInfected
Desulfovibrio	glucose	-7.75E-05	-0.0003741	0.00021902	-0.0929017	-0.4482295	0.26242612	0.59833686	UnInfected
CAG.352	BP_Sys	0.00237317	-0.0021911	0.00693748	0.15667343	-0.1446564	0.45800325	0.29781715	UnInfected
CAG.352	BP_Dia	0.00246327	-0.0008876	0.00581419	0.23622586	-0.0851245	0.55757625	0.14426613	UnInfected
CAG.352	Chol_T	-0.0002516	-0.0005738	7.05529190361786e-05	-0.2207885	-0.5034823	0.06190535	0.12159903	UnInfected
CAG.352	Chol_L	-0.0001634	-0.0004507	0.0001239	-0.1624009	-0.4479254	0.12312366	0.25550124	UnInfected
CAG.352	insulin	-0.0021939	-0.0039788	-0.000409	-0.3763047	-0.6824567	-0.0701527	0.01753728	UnInfected
CAG.352	glucose	-9.69E-05	-0.0003086	0.00011482	-0.1560108	-0.496878	0.18485651	0.35852692	UnInfected
Eubacterium_eligens_group	BP_Sys	0.00335356	-0.0043739	0.01108097	0.13770825	-0.1796052	0.45502168	0.38365431	UnInfected
Eubacterium_eligens_group	BP_Dia	0.00185855	-0.0039372	0.00765431	0.11086072	-0.2348495	0.45657098	0.51865029	UnInfected
Eubacterium_eligens_group	Chol_T	0.00048645	-4.97E-05	0.00102257	0.26548499	-0.0271057	0.55807572	0.07387373	UnInfected
Eubacterium_eligens_group	Chol_L	0.0002124	-0.0002756	0.00070036	0.13128384	-0.1703336	0.43290123	0.38226469	UnInfected
Eubacterium_eligens_group	insulin	0.00083019	-0.0024359	0.00409631	0.08856998	-0.2598815	0.43702148	0.60850997	UnInfected
Eubacterium_eligens_group	glucose	-0.0001728	-0.0005289	0.00018329	-0.1730504	-0.5296419	0.18354116	0.33066508	UnInfected
Asteroleplasma	BP_Sys	0.00106931	-0.000633	0.00277158	0.18220433	-0.1078529	0.47226152	0.21016098	UnInfected
Asteroleplasma	BP_Dia	0.00118403	-4.76E-05	0.00241569	0.29306701	-0.0117885	0.59792251	0.0589934	UnInfected
Asteroleplasma	Chol_T	8.93347369582864e-07	-0.0001247	0.0001265	0.00202312	-0.2824327	0.28647895	0.98854216	UnInfected
Asteroleplasma	Chol_L	4.23997323888692e-05	-6.67E-05	0.0001515	0.10875036	-0.1710809	0.38858159	0.4347821	UnInfected
Asteroleplasma	insulin	0.00030159	-0.000422	0.00102522	0.13351236	-0.1868415	0.45386623	0.40259353	UnInfected
Asteroleplasma	glucose	-2.74E-06	-8.33E-05	7.78464373713779e-05	-0.0114051	-0.346275	0.32346486	0.94517551	UnInfected
Enterobacter	BP_Sys	-0.0006053	-0.0054175	0.00420697	-0.0403934	-0.3615428	0.28075614	0.79961952	UnInfected
Enterobacter	BP_Dia	-0.0007868	-0.0043699	0.00279629	-0.0762725	-0.4236206	0.2710756	0.65797423	UnInfected
Enterobacter	Chol_T	-0.0005403	-0.0008297	-0.0002508	-0.4792021	-0.7359096	-0.2224945	0.00059487	UnInfected
Enterobacter	Chol_L	-0.0003597	-0.0006359	-8.34E-05	-0.361298	-0.6388051	-0.0837908	0.01229677	UnInfected
Enterobacter	insulin	-0.0015427	-0.0034879	0.00040257	-0.2674757	-0.6047522	0.06980082	0.1161672	UnInfected
Enterobacter	glucose	-3.56E-06	-0.0002262	0.00021907	-0.0057886	-0.3680856	0.35650838	0.97426398	UnInfected

Megamonas	BP_Sys	0.00588608	-0.0032953	0.0150675	0.18684496	-0.1046053	0.47829521	0.20115507	UnInfected
Megamonas	BP_Dia	-0.0010985	-0.0081121	0.00591522	-0.050651	-0.3740578	0.27275587	0.75200794	UnInfected
Megamonas	Chol_T	-0.000106	-0.0007831	0.00057108	-0.0447294	-0.3303933	0.24093453	0.75206358	UnInfected
Megamonas	Chol_L	-0.0005065	-0.0010734	6.03993796045094e-05	-0.2420067	-0.5128736	0.02886025	0.07819255	UnInfected
Megamonas	insulin	0.00187219	-0.0020209	0.00576529	0.15440461	-0.1666706	0.47547978	0.33499725	UnInfected
Megamonas	glucose	-6.54E-05	-0.0004999	0.00036916	-0.050597	-0.3869522	0.2857583	0.76149189	UnInfected
Methanosphaera	BP_Sys	-0.0006382	-0.0043487	0.00307233	-0.0537408	-0.3661929	0.2587114	0.72861479	UnInfected
Methanosphaera	BP_Dia	0.00110787	-0.0016377	0.00385348	0.13551385	-0.2003277	0.47135536	0.41757292	UnInfected
Methanosphaera	Chol_T	3.34871670671095e-05	-0.000234	0.00030098	0.03747767	-0.2618892	0.33684458	0.80053504	UnInfected
Methanosphaera	Chol_L	5.6616662853316e-05	-0.0001773	0.00029052	0.07176368	-0.2247164	0.36824371	0.62565541	UnInfected
Methanosphaera	insulin	-0.0012627	-0.0027565	0.00023106	-0.2762515	-0.6030536	0.05055059	0.09483396	UnInfected
Methanosphaera	glucose	-5.36E-05	-0.0002244	0.00011712	-0.110131	-0.4607627	0.24050065	0.52721606	UnInfected

Log2FoldChange from Differential Abundance analysis	Adjusted p. value for microbiota	*S. mansoni* Group where taxa is more abundant	Taxa associated with CVD risk	Metabolite associated with CVD	Microbiota-metabolome correlation value	Associated CVD risk
2.1867	2.5768e-12	Infected	*Altererythrobacter*	HMDB31050	-0.830	DIASTOLIC BP
2.1129	7.2807e-11	Infected	*Arthrobacter*	HMDB31050	-0.850	DIASTOLIC BP
1.3364	9.5039e-06	Infected	*Devosia*	HMDB31050	-0.819	DIASTOLIC BP
1.4811	6.2449e-07	Infected	*Domibacillus*	HMDB32627	-0.800	DIASTOLIC BP
2.1777	9.7252e-11	Infected	*Ellin6055*	HMDB31050	-0.821	DIASTOLIC BP
1.0694	0.0006	Infected	*Geodermatophilus*	HMDB31050	-0.813	DIASTOLIC BP
1.0117	0.0001	Infected	*Kapabacteriales*	HMDB32627	-0.767	DIASTOLIC BP
1.5948	1.8261e-07	Infected	*Kribbella*	HMDB31050	-0.797	DIASTOLIC BP
1.0321	0.0006	Infected	*Longimicrobiaceae*	HMDB31050	-0.798	DIASTOLIC BP
2.6592	2.4792e-16	Infected	*Lysobacter*	HMDB31050	-0.853	DIASTOLIC BP
1.0511	0.0034	Infected	*Nitrospira*	HMDB31050	-0.810	DIASTOLIC BP
1.8145	4.4458e-07	Infected	*Pseudarthrobacter*	HMDB31050	-0.826	DIASTOLIC BP
3.8699	8.8352e-22	Infected	*Vicinamibacteraceae*	HMDB32627	-0.777	DIASTOLIC BP
2.2985	2.79650e-11	Infected	*Gaiella*	0.95_764.7010n	-0.671	LDL CHOLESTEROL
2.2985	2.7965e-11	Infected	*Gaiella*	HMDB56087	-0.655	LDL CHOLESTEROL
1.6089	3.9925e-08	Infected	*Arenimonas*	0.95_764.7010n	-0.653	LDL CHOLESTEROL

Supplementary table 4: Differentially Abundant Gut Microbiota in *S. mansoni*-infected individuals and their correlation with cardiovascular risk-associated metabolites. This table shows gut microbial taxa that were significantly more abundant in *S. mansoni*–infected (n=128) compared with uninfected individuals (n=81), as identified by differential abundance analysis

using two-sided statistical testing, with $\log_2$ fold change > 1 and false discovery rate (FDR)–adjusted $P \leq 0.05$. For each taxon, associated metabolites and cardiovascular disease (CVD) risk factors are reported as depicted in Fig. 8. Associations between microbial taxa and metabolites were assessed using two-sided Spearman's rank correlation analysis; correlation coefficients (r) and exact P values were calculated for each pairwise association. No additional adjustment for multiple comparisons was applied at the correlation stage. Negative correlation coefficients indicate inverse relationships between microbial abundance and metabolite levels. CVD risk factors include diastolic blood pressure and low-density lipoprotein (LDL) cholesterol.
